# Dissipative Formation of Covalent Basket Cages

**DOI:** 10.1002/anie.202207418

**Published:** 2022-07-11

**Authors:** Vageesha W. Liyana Gunawardana, Tyler J. Finnegan, Carson E. Ward, Curtis E. Moore, Jovica D. Badjić

**Affiliations:** ^1^ Department of Chemistry & Biochemistry The Ohio State University 100 West 18th Avenue Columbus OH 43210 USA

**Keywords:** Cavitands, Dynamic Covalent Chemistry, Dynamic Self Assembly, Porous Organic Cages, Out of Equilibrium Systems

## Abstract

Living systems use chemical fuels to transiently assemble functional structures. As a step toward constructing abiotic mimics of such structures, we herein describe dissipative formation of covalent basket cage CBC **5** by reversible imine condensation of cup‐shaped aldehyde **2** (i.e., basket) with trivalent aromatic amine **4**. This nanosized [4+4] cage (*V*=5 nm^3^, *M*_w_=6150 Da) has shape of a truncated tetrahedron with four baskets at its vertices and four aromatic amines forming the faces. Importantly, *tris*‐aldehyde basket **2** and aliphatic *tris*‐amine **7** undergo condensation to give small [1+1] cage **6**. The imine metathesis of **6** and aromatic *tris*‐amine **4** into CBC **5** was optimized to bias the equilibrium favouring **6**. Addition of tribromoacetic acid (TBA) as a chemical fuel perturbs this equilibrium to result in the transient formation of CBC **5**, with subsequent consumption of TBA via decarboxylation driving the system back to the starting state.

## Graphical abstract

Dynamic covalent basket cages can now be made to exist for a tunable period of time as a function of the chemical fuel used to drive their formation. The stage is thus set to explore the functional characteristics of these fascinating molecules and mimic the action of natural systems capable of directing folding, catalysis, and transport in a spatiotemporal manner.

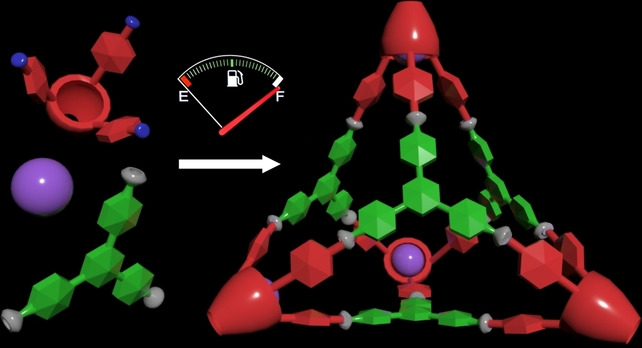

## Introduction

**Figure 1:**
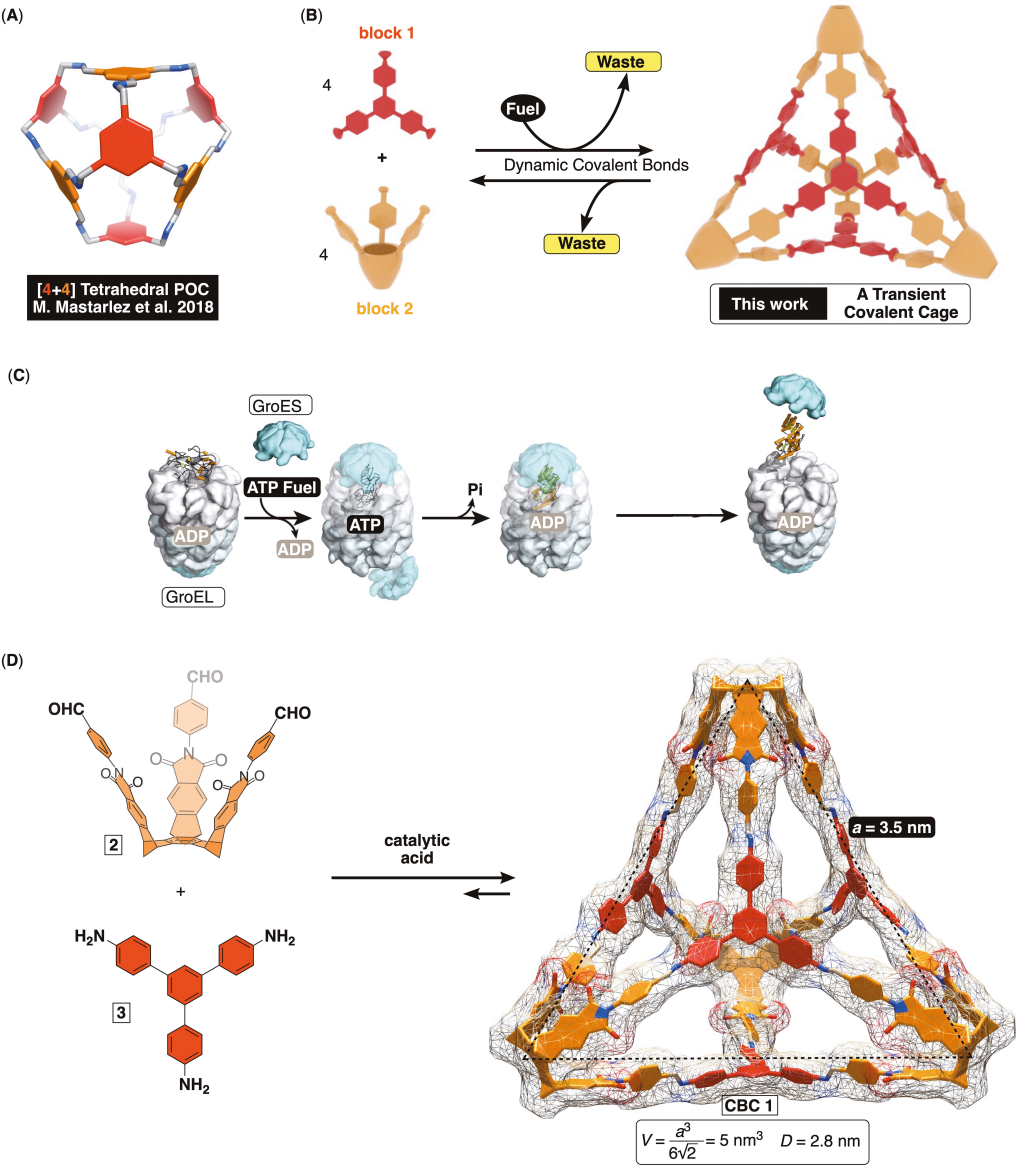
A) An energy‐minimized structure (PM3, Spartan) of a previously studied [4+4] porous organic cage (POC). B) A scheme showing the formation of a covalent cage driven by chemical fuel. C) GroEL is a biological molecular machine that uses ATP as a fuel for forming a transient intermediate capable folding a protein in its cavity. The hydrolysis of ATP drives the departure of the folded molecule. D) The proposed formation of covalent basket cage **1** (CBC **1**, PM3) from 1,3,5‐*tris*‐(4‐aminophenyl)benzene **3** and *tris*‐aldehyde basket **2**. CBC **1** is a truncated tetrahedron with a trigonal side panel having edges *a*=3.5 nm and volume 5 nm^3^. Diameter of a circumscribed sphere is *D*=2.8 nm.

Porous organic cages (POCs)^[1]^ are discrete molecular structures composed of two or more multivalent organic components often linked via labile covalent bonds (Figure 1A).^[2]^ These fascinating compounds are rigid enough^[3]^ to retain their shape in the solid state and give micro‐ and mesoporous materials comprising hollow interconnected network of channels.^[4]^ In contrast to conventional porous frameworks (MOFs, COFs, HOFs, etc.),^[5]^ solution processibility of POCs has facilitated their implementation in devices for gas separation^[6]^ and sensing^[7]^ of organic compounds. Thus far, the studies pertaining POCs have mostly focused on investigating their capacity for trapping gases under equilibrating conditions.[^1^, ^3^] However, one can envision larger POCs built from cavitands^[8]^ (Figure 1B) and acting as hosts for encapsulation of one or more pharmaceuticals, toxins or even biological macromolecules (proteins, nucleic acids, etc.),[^8d^, ^9^] promoting folding^[10]^ or catalysis.^[11]^

Emergence of complexity in natural systems arises from vast networks of dynamic assemblies^[12]^ operating out‐of‐equilibrium.^[13]^ For instance, the binding of ATP (i.e. chemical fuel, Figure 1C) to chaperonin produces a transient intermediate capable of encapsulating an unfolded protein (along with GroES lid) to direct its folding within the chamber.^[14]^ With GroEL acting as an ATPase, the hydrolysis of ATP triggers the departure of now folded protein (and the GroES lid) followed by the binding of subsequent ATP to repeat the process. To mimic the complex natural machinery,^[15]^ a variety of self‐assembled systems^[16]^ and autonomous molecular machines^[17]^ have, in recent years, been developed to operate “out of equilibrium”. A particularly intriguing system was recently described by Nitschke and co‐workers wherein a small metal–organic cage was in the presence of triphenylphosphine fuel disassembled and then assembled back by dissipation of such fuel.^[18]^ In this regard, we reasoned that learning about transient formation of large POCs with inner space composed of cavitands (Figure 1B) would set a stage for achieving fuel driven catalysis,^[19]^ folding^[10]^ and sequestration.^[20]^ Thus, if an equilibrating system is set to favor the building components of such cage (Figure 1B), then chemical fuel could be added to push the equilibrium out of balance giving rise to the cage which dissipates as the fuel is consumed.^[21]^ Several challenges in constructing an abiotic dissipative system of this kind include: a) configuring the equilibrium in Figure 1B to favor reactants, b) a chemical reaction cycle^[15a]^ that incorporates rapid formation and slow breakdown of the covalent cage and c) having the synthesis of the covalent cage proceed with high fidelity^[22]^ to allow continuous operation^[23]^ since an irreversible loss of the material would hamper its effectiveness. In regard to the last point, POCs are obtained via single‐pot syntheses^[24]^ using polyvalent components capable of, in most cases, forming imine and/or boronic ester bonds in a reversible fashion.^[25]^ Dynamic equilibria are important for correcting errors,^[26]^ albeit the formation of kinetic traps^[22]^ is possible necessitating optimization of the reaction conditions. Since the outcome of reversible‐bond condensations correlates well with the degree of preorganization of the reacting molecules,[^8b^, ^27^] we hypothesized that trivalent *tris*‐aldehyde basket **2**,^[28]^ with bite angle^[22]^ close to 60° (Figure 1D) and semi‐flexible bicyclic framework,^[29]^ could undergo imine condensation with trivalent 1,3,5‐*tris*‐(4‐aminophenyl)benzene **3** to give covalent basket cage **1** (CBC, Figure 1D); CBCs are herein introduced as a subclass of POCs. Nanosized **1** (*d*=3.0 nm and *V*=5 nm^3^) is a truncated [4+4] tetrahedron^[30]^ with four trigonal panels made of triphenyl benzenes and four vertices composed of abiotic cavitands, baskets.^[31]^ Baskets are known to act as allosteric hosts^[29]^ capable of trapping haloalkanes,^[32]^ cationic molecules,^[10]^ nerve agents,^[20c]^ pesticides,^[33]^ and anticancer drugs.^[20b]^ Accordingly, we reasoned that obtaining non‐collapsible CBCs of type **1** and developing a method for their transient formation via consumption of chemical fuel will set the stage for examining temporal control of their action,^[34]^ resembling GroEL chaperone in Figure 1C.^[18]^

## Results and Discussion

### Synthesis and Characterization of Covalent Basket Cages (CBCs)

**Figure 2:**
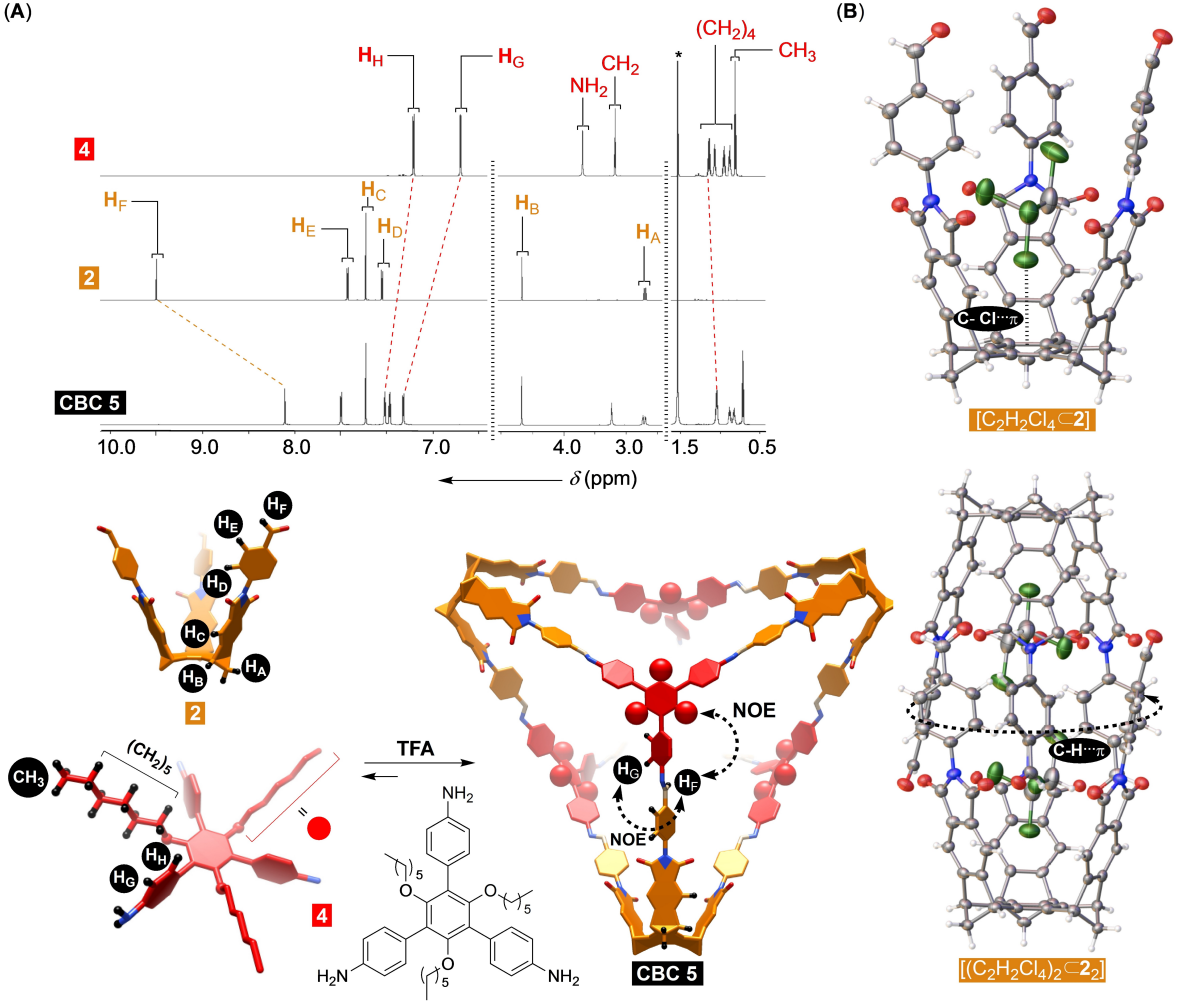
A) ^1^H NMR spectra (850 MHz, CD_2_Cl_2_) of *tris*‐amine **4** (top), *tris*‐aldehyde basket **2** (middle), and CBC **5** (bottom). Energy‐minimized structures (PM3) of **2**, **4** and **5** (hexoxide groups are shown as red spheres). B) ORTEP diagrams (50 % probability) of the solid‐state structures of complexes [C_2_H_2_Cl_4_⊂**2**] and [(C_2_H_2_Cl_4_)_2_⊂**2**_2_].

After adding *tris*‐aldehyde **2** to *tris*‐amine **3** (Figure 1D) in DMSO, the condensation took place giving oligomeric materials, although mass spectrometry (MALDI, Figure S1) showed that desired CBC **1** formed as a minor product. Encouraged by the result, we decided to probe the condensation in differently sized and shaped solvents since, we posited, they might template the formation of the [4+4] cage. From solvent screening (Figure S2), it appeared that 1,2‐dichloroethane (DCE) and chloroform would, in the presence of catalytic TFA,^[35]^ assist the formation of CBC **1**. Despite such optimization, cage **1** precipitated from the solution as a pale‐yellow solid being sparingly soluble in organic media. To address the issue, we prepared *tris*‐amine **4** (Figure 2A) to include solubilizing groups (hexoxide, OC_6_H_13_) appended to the benzene core. In DCE containing catalytic TFA, the reaction of *tris*‐aldehyde basket **2** and *tris*‐amine **4** resulted in the formation of CBC **5** as the sole product (Figure 2A; Figure S21), which remained soluble in chlorinated solvents after isolation, including dichloromethane and chloroform. ^1^H NMR spectrum of CBC **5** showed a set of signals corresponding to, on average, a *T*_d_ symmetric imine (Figure 2A; for 2D COSY, HMBC and HSQC of CBC **5** see Figures S8–10) with resonances arising from the basket and linker components in equal ratio. A greater magnetic deshielding of **H**_G/H_ signals from *tris*‐imine panels is in line with the conversion of the amine functional groups into more electron withdrawing imines. From the 2D NOESY spectrum of **5** (Figure S11), we noted cross peaks between **H**_F_ and **H**_G_ as well as **H**_F_ and **CH**_2_ from hexyl groups to corroborate the proximity of the two building blocks and *E* configuration of the imine double bond (Figure 2A). The DOSY NMR spectrum of CBC **5** had all proton resonances leveled (Figure S12) therefore corroborating that the amine and aldehyde components are within the same molecule.

### Molecular Encapsulation within CBC 5

**Figure 3:**
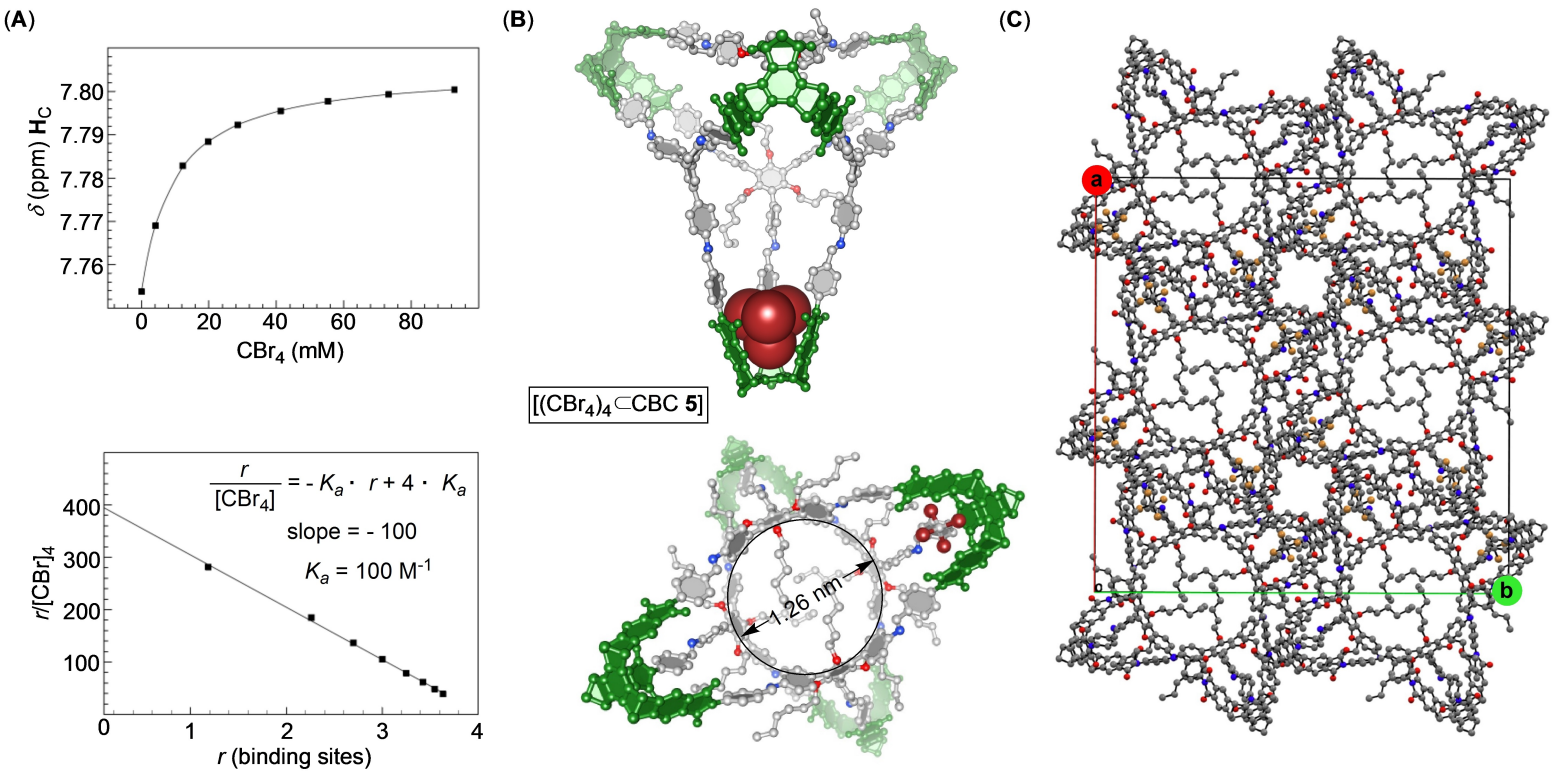
A) An incremental addition of a standard solution of CBr_4_ to CBC **5** (0.15 mM) was in CD_2_Cl_2_ monitored with ^1^H NMR spectroscopy (600 MHz, 300 K). A nonlinear least‐square analysis of the binding isotherm (SigmaPlot; Figure S23) fit well to the formation of a binary complex with *K*_a_=108±2 M^−1^ (mean ± standard deviation from two independent measurements); note that for the analysis, the concentration of CBC **5** was multiplied by a factor of four. A Scatchard plot for the supramolecular titration of CBr_4_ to CBC **5** was fit to a linear function (SigmaPlot); with one measurement completed, there is no standard deviation to report. The population of the four binding sites in CBC **5** (*r*=1–4) was calculated using the observed change in the chemical shift of **H**_C_ (top) while the equilibrium concentration of [CBr]_4_ was assumed to be equal to its overall concentration. B) A ball‐and‐stick representation of CBC **5** in the solid state (X‐ray diffraction) with four CBr_4_ molecules in its cavity (only one is shown for clarity). C) A ball‐and‐stick representation of the unit cell of CBC **5** in the solid state, showing cylindrical channels extending along the crystallographic c axis.

A slow vapor diffusion of methanol into 1,1,2,2‐tetrachloroethane (TCE) solution of *tris*‐aldehyde basket **2** gave single crystals. After being subjected to X‐ray diffraction analysis (Figure 2B), we found the unit cell of **2** includes two baskets entangled into a centrosymmetric dimer surrounded with six additional dimers as a part of a honeycomb array (Figure 2B). Each dimeric assembly incorporates two molecules of TCE, holding onto southern and northern benzenes via C−Cl⋅⋅⋅π halogen bond (*R*=3.334 Å and *α*=171.67°).^[36]^ Benzaldehyde groups employ C_sp2_−H groups to form a seam of edge‐to‐face C_sp2_−H⋅⋅⋅π hydrogen bonds at the equator (*d*_c−π_=3.705–4.418 Å and *α*=112.41–143.59°, Figure 2B).^[37]^ With tetrachloroethane (108 Å^3^) residing in the cavity of **2** and the known propensity of baskets to trap haloalkanes in solution,^[38]^ we wondered if CBC **5** could use its four compartments for complexing complementary and polarizable CBr_4_ (108 Å^3^).^[39]^ If so, could there be any homotopic cooperativity characterizing the four consecutive binding events?^[20a]^ An incremental addition of a standard solution of CBr_4_ to tetravalent CBC **5** caused a notable magnetic deshielding of its aromatic **H**_C_ protons (Figure 2A; Figure S23) resulting from the guest occupying the host's cavities.^[39]^ The titration isotherm fit well to 1 : 1 binding model (*K*_a_=108±2 M^−1^, Figure 3A) with the linear Scatchard plot^[40]^ corroborating the statistical population of all four compartments (*K*_a_=100 M^−1^, Figure 3A); for fitting to 1 : 1 binding model, the known concentration of CBC **5** was multiplied by a factor of four. Interestingly, the complexation of CBr_4_ by CBC **5** (*K*_a_=108±2 M^−1^) was more favorable than by basket **2** (*K*_a_=46±4 M^−1^, Figure S24). We posit that a more rigid and preorganized cavities of CBC **5** (Figure 2A) could be responsible for the stronger binding. As for the statistical complexation of CBr_4_ by tetravalent CBC **5**, the consecutive binding events must have caused insufficient change in the conformation of the cage and its solvation to result in measurable outcome.^[41]^

A slow diffusion of methanol into 1,1,2,2‐tetrachloroethane solution of CBC **5** containing CBr_4_ resulted in the formation of single crystals. X‐ray diffraction analysis of the sample revealed rigid CBC **5** with the shape of a truncated tetrahedron having four molecular baskets at its corners conjugated to four trivalent aromatic panels by imine bonds (Figure 3B). Fascinatingly, each CBC **5** would in the solid state encapsulate four molecules of CBr_4_: these guests are nested inside basket cavitands forming a C−Br⋅⋅⋅π halogen bond (*R*=3.229 Å and *α*=170.09°)^[36]^ with the benzene base and placing the remaining three bromides between the phthalimide sides.^[42]^ The unit cell has four [(CBr)_4_⊂**5**] complexes (Figure 3C) packed in an arrangement that forms nanosized channels (1.26 nm wide, Figure 3C) extending throughout the entire crystal. The channels are lined with four conformationally dynamic hexyl chains, two at the front and two at the back (Figure 3C). The solid material is thus expected to be porous^[4]^ (i.e. POC) with its channels providing access to basket cavitands. The uptake of potential guests (i.e., gas molecules or compounds from a liquid phase)^[43]^ remains to be studied in the future.

### Fuel‐Driven Formation of CBCs

**Figure 4:**
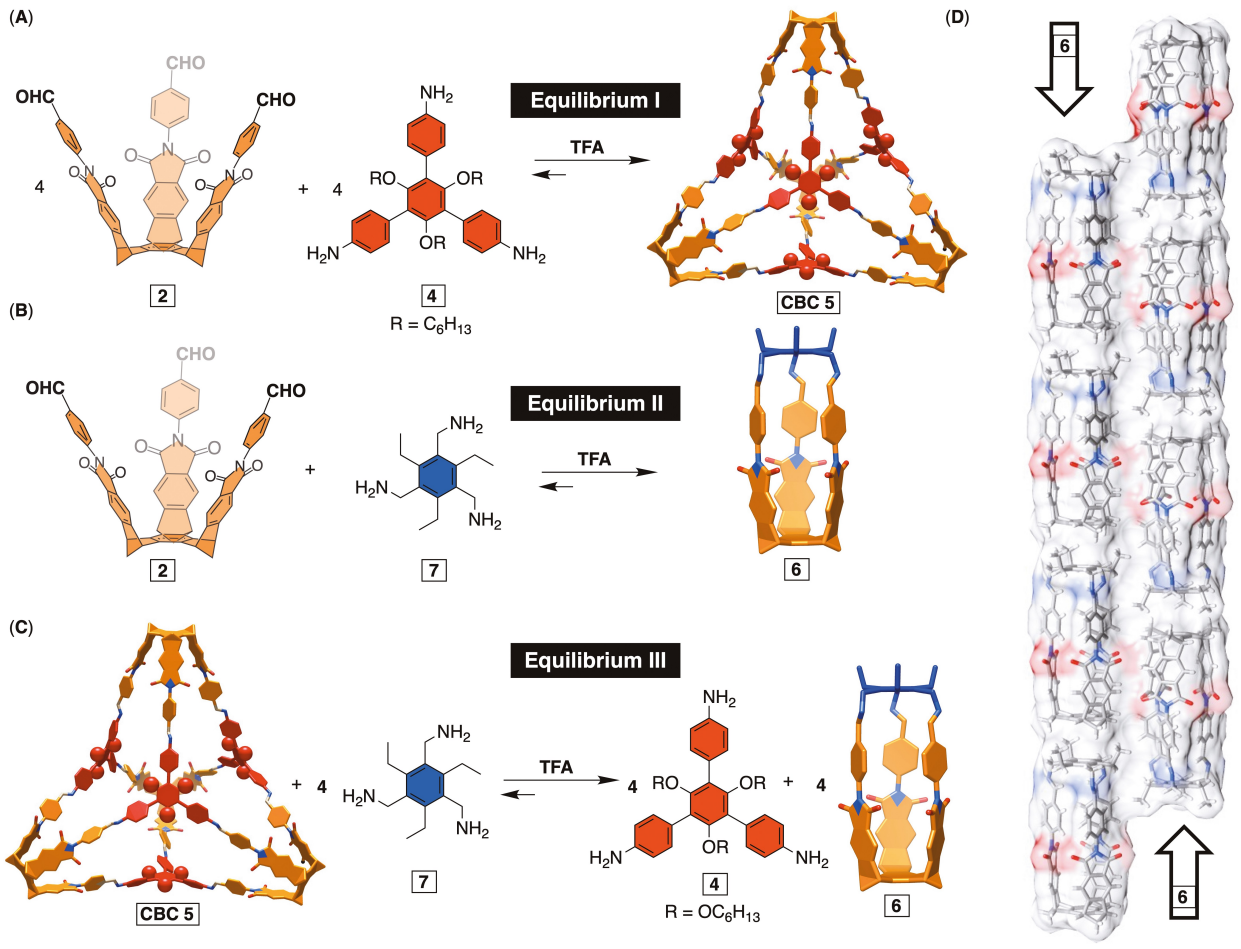
A) Equilibrium I is populated with *tris*‐amine **4**, *tris*‐aldehyde basket **2** and CBC **5** and shifted toward the cage formation at 298 K. B) Equilibrium II is populated with *tris*‐amine **7**, *tris*‐aldehyde basket **2** and small [1+1] cage **6** and shifted toward this cage formation at 298 K. C) Equilibrium III includes CBC **5** reacting with *tris*‐amine **7** to give *tris*‐amine **4** and [1+1] cage **6**. With an excess of **7**, equilibrium III is shifted to the right with predominant formation of [1+1] cage **6**. D) A stick representation of the solid‐state structure of [1+1] cage **6** packing into supramolecular nanotubes.

With a catalytic amount of TFA, the equilibrium containing *tris*‐aldehyde **2** and *tris*‐amine **4** favors the formation of CBC **5** (equilibrium I, Figure 4A). However, adding a large excess of TFA caused CBC **5** disassemble into **2** and [**4**‐H_3_]^3+^ driven by the favorable protonation of the *tris*‐amine (Figure S22). Subsequent addition of a base (Et_3_N) resulted in the deprotonation of [**4**‐H_3_]^3+^, followed by the exclusive reformation of CBC **5**. We recognized that the pH‐dependent manipulation of this equilibrium could allow us to transiently form CBC **5** using an acid that dissipates over time.[^16c^, ^44^] However, the addition of an excess of labile acid to equilibrium I would result in the disassembly of CBC **5**, while we desired the reverse.^[45]^

To address this issue, we turned to our discovery of a smaller [1+1] cage **6** (equilibrium II, Figure 4B) via the reaction between aliphatic *tris*‐amine **7** and basket **2** in the presence of catalytic TFA (Figures S14–S20).[^27a^, ^46^] Size‐shape complementarity^[47]^ of the basket and linker component **7** produced [1+1] cage **6** under thermodynamic control. The solid‐state structure of **6** (X‐ray diffraction, Figure 4D) showed these hollow cages with no guests occupying their interior. Interestingly, they packed with a tubular arrangement by stacking (head‐to‐tail, Figure 4D) on top of one another. The tubes also extend throughout the crystal along the crystallographic *a* axis in the opposite directions.

Since both [4+4] cage **5** and [1+1] cage **6** use *tris*‐aldehyde **2** as a subunit, we hypothesized that combining *tris*‐amine **4** and *tris*‐amine **7** in the same reaction would result in an equilibrium between the two cages under TFA catalysis (equilibrium III, Figure 4C). This equilibrium could be biased to favor cage **6** by adding an excess of *tris*‐amine **7**. On the contrary, the addition of an excess of TFA would preferentially protonate aliphatic *tris*‐amine **7** over aromatic *tris*‐amine **4** to shift equilibrium III back and in favor of forming CBC **5**. It follows that using an acid that decomposes over time could permit a transient conversion of cage **6** to CBC **5** followed by a reversion to **6** as the acid is consumed.

**Figure 5:**
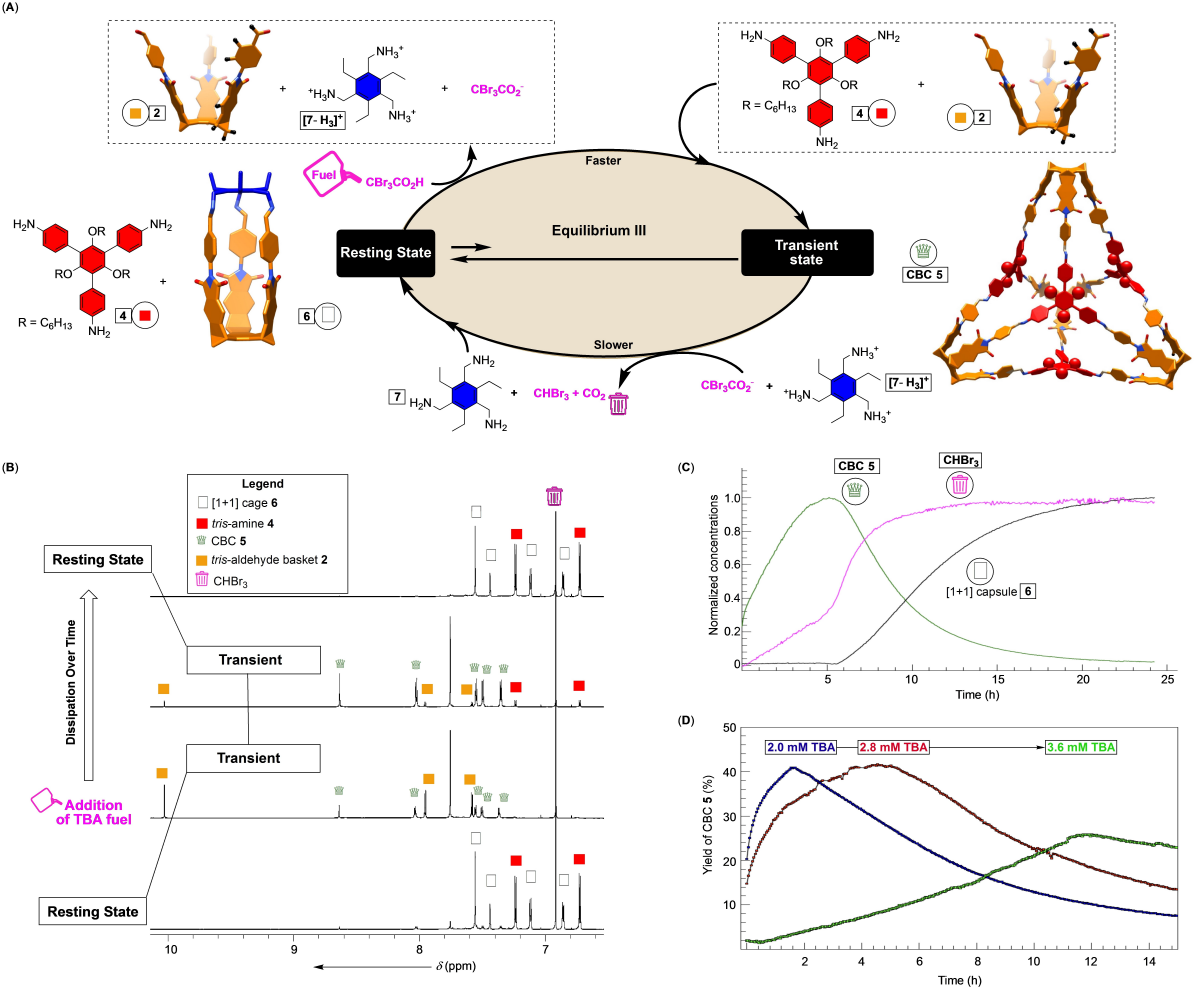
A) A chemical reaction cycle showing tribromoacetic acid (TBA) acting as a fuel and driving equilibrium III to the right for a transient formation of CBC **5**. Decarboxylation of CBr_3_CO_2_^−^ into CHBr_3_ and CO_2_ (waste) brings the system back to its original state. B) Partial ^1^H NMR spectra (850 MHz, CD_2_Cl_2_) of [1+1] cage **6** (0.44 mM), *tris*‐amine **4** (0.44 mM), *tris*‐amine **7** (0.62 mM) and TFA (1.7 mM); for the residual amount of CHBr_3_, see Figures S24, S25. After an addition of TBA fuel (2.5 mM), ^1^H NMR spectra were recorded after a few minutes, 5 h and 24 h (see also Figures S25–S28). C) A plot showing a normalized change in the concentration of [1+1] cage **6**, CBC **5** and CHBr_3_ over time for the experiment described in (B). D) A plot showing a change in the yield of CBC **5** over time for experiments similar to that described in (B) repeated with different concentrations of TBA: 2.0 mM (blue), 2.8 mM (red) and 3.6 mM (green) (see also Figures S32–S39).

After an excess of aliphatic *tris*‐amine **7** and catalytic TFA were added to CBC **5**, we noted that cage **6** dominated equilibrium III (Figure 4C). As implied above, an excess of **7** was needed to push the equilibrium to the right, while proper amount of TFA to maintain its concentration in solution at the level necessary to give [1+1] cage **6** (for details, see Supporting Information section on page S31 and Figures S25 and S26). Importantly, a greater quantity of TFA shifted equilibrium III to the left with the removal of **6** and the formation of CBC **5** (Figures S25 and S26). As the amount of TFA tunes the state of equilibrium III in solution, we turned to probe the action of labile tribromoacetic acid (TBA, chemical fuel, p*K*_a_=0.8) hoping that it would provide the same level of control. That is to say, an excess of TBA was expected to disturb equilibrium III (Figure 5A) by predominantly protonating aliphatic amine **7** (p*K*_a_=9) thereby triggering the removal of the smaller [1+1] cage **6**. Condensation of the released *tris*‐aldehyde basket **2** and aromatic amine **4** (p*K*_a_=4) should then result in the transient formation of CBC **5**. The tribromoacetate ion formed through protonation of **7** undergoes thermal decarboxylation to give CHBr_3_ and CO_2_ (waste) with the overall loss of acid in solution. The resulting deprotonation of aliphatic *tris*‐amine **7** restores the original equilibrium III dominated by aromatic *tris*‐amine **4** and [1+1] cage **6**. In order to build a larger quantity of CBC **5** in solution, a faster degradation of **6** followed by slower dissipation of **5** must take place during the proposed reaction cycle (Figure 5A).^[15a]^

^1^H NMR spectrum of [1+1] cage **6**, *tris*‐amines **4** and **7** along with TFA in CD_2_Cl_2_ showed the presence of **6** and **4** while [**7**‐H_*n*_]^*n*+^ (*n*=1–3) stayed as a precipitate (Figure 5B). The addition of TBA (fuel) prompted the immediate disintegration of [1+1] cage **6** (Figure 5B). At the same time, CBC **5** started to form with a steady increase in its concentration over time (Figure 5B/C). When the concentration of **5** peaked (ca. 5 h, Figure 5C), the decarboxylation of tribromoacetate, illustrated by the formation of CHBr_3_ (Figure 5C), became faster resulting in the release of *tris*‐amine **7** and concomitant formation of [1+1] cage **6**. After circa 20 h, the system returned to its resting state comprising **6**, **4**, and [**7**‐H_*n*_]^*n*+^ (*n*=1–3) with CO_2_ and CHBr_3_ formed as waste. The process was successively repeated three times using the same reaction mixture with the concentration of [1+1] cage **6** staying consistent at 0.24–0.28 mM at the end of each cycle (Figures S40–S42). Since only 60–80 % of the fuel was spent during each cycle, we had to adjust the amount of TBA to study its recyclability. As a result of this caveat and the kinetics of the reaction being highly responsive to the amount of acid, we noted that the quantity of CBC **5** dropped from cycle 1 to 3 at the 5 h mark (0.053 to 0.021 mM, Figure S40–S42). As recyclability is essential for the application of these systems in the future, the apparent, and still unclear, drop in the effectiveness needs to be further investigated; in this regard, the knowledge of the reactions’ kinetics is necessary for understanding and optimizing the outcome of coupled chemical processes, which we herein completed by using trial‐and‐error method. By using different quantities of TBA fuel at the beginning of the experiment, we found that the formation of CBC **5** could be adjusted to peak at 1.7, 4.5 and 12 h (Figure 5D). Additional fuel requires longer time to convert into waste (second step in Figure 5A) therefore slowing the release of **7** and prolonging the degradation of CBC **5**. Such fine tuning of the time‐dependent concentration of CBCs may come handy for optimizing catalysis, delivery, and sequestration in complex chemical environments.^[48]^

## Conclusion

Covalent basket cages (CBCs) can now be made to exist for a period of time,^[15a]^ using chemical fuel to tune the kinetics of their formation and degradation. By combining trivalent and planar amines with trivalent and cup‐shaped aldehydes (i.e., baskets) we obtained spacious [4+4] cages as rigid truncated tetrahedrons. These CBCs include four baskets at their vertices with four trivalent amines forming the face. In both solution and solid state, CBC **5** acted as an allosteric host holding up to four guest molecules in its four cavities. With the assistance of small [1+1] cage **6**, we established an equilibrium to favor its formation at the expense of CBC **5**. The addition of tribromoacetic acid as fuel shifted the equilibrium toward the formation of CBC **5** and after the fuel dissipated by decarboxylation, the system returned to the starting equilibrium state. This particular strategy for transient formation of CBCs can, perhaps, be applied toward designing the temporal preparation of other POCs. While the system remains a proof of concept, the long‐term goal is to exploit the functional characteristics^[34]^ of dynamic covalent cages for promoting chemical reactions,^[49]^ folding of molecules,^[10]^ delivery,^[50]^ and sequestration^[20c]^ in spatiotemporal fashion. After all, natural systems^[51]^ have evolved to use dynamic materials operating out‐of‐equilibrium and capable of doing work: converting chemical into mechanical energy (cytoskeleton),^[52]^ folding proteins (GroEL chaperone)^[14]^ and controlling membrane transport (rotary ATPases).^[53]^

## Conflict of interest

The authors declare no conflict of interest.

1

## Supporting information

As a service to our authors and readers, this journal provides supporting information supplied by the authors. Such materials are peer reviewed and may be re‐organized for online delivery, but are not copy‐edited or typeset. Technical support issues arising from supporting information (other than missing files) should be addressed to the authors.

Supporting Information

Supporting Information

Supporting Information

Supporting Information

## Data Availability

The data that support the findings of this study are available on request from the corresponding author. The data are not publicly available due to privacy or ethical restrictions.
