# Supplementary material for: Dissipative Formation of Covalent Basket Cages
Source: Angew Chem Int Ed Engl. 2022 Jul 11;61(33):e202207418. doi: 10.1002/anie.202207418 (PMC9544755; doi:10.1002/anie.202207418)
Supplement: Supplementary file 4 — Supporting Information [file ANIE-61-0-s001.pdf]

Angewandte Chemie
Eine Zeitschrift der Gesellschaft Deutscher Chemiker

Supporting Information

Dissipative Formation of Covalent Basket Cages

*V. W. Liyana Gunawardana, T. J. Finnegan, C. E. Ward, C. E. Moore, J. D. Badjić**

Fuel-Driven Formation of Covalent Basket Cages

Vageesha W. Liyana Gunawardana,[a] Tyler J. Finnegan, [a] Carson E. Ward,[a] Curtis E. Moore,[a] Jovica D. Badjić*[a]

[a]Department of Chemistry & Biochemistry, The Ohio State University, 100 West 18th Avenue, 43210 Columbus, Ohio

Supplementary Information

Table of Contents

General Information 3
Syntheses of Basket **2** and Linker **4** 4
Probing the Synthesis of [4+4] Cage **1** 6
Solvent Screen for Synthesis of [4+4] Cage **1** 7
Synthesis of CBC 5 and [1+1] Capsule **6**...9
Monitoring the Formation of CBC **5** with ^{1}H NMR Spectroscopy 27
Reversible Formation/Degradation of CBC **5**..28
Supramolecular Titrations with CBr_4 29
Switching Between [1+1] cage **6** and CBC **5** 31
Fuel-Driven Reaction Cycles 33
Dissipation Cycles with Different Amounts of TBA...37
Successive Repetitions of Dissipative Cycles 43
X-Ray Crystallography...45
References...126

General Information

All chemicals were purchased from commercial sources and used as received unless stated otherwise. Flash chromatography was performed with silica gel (SiO_2, 40-63 µm, 230-400 mesh, 60 Å) from SiliCycle (Quebec, Canada). Thin layer chromatography was performed on silica gel, glass-backed plates with fluorescent indicator (Sigma Aldrich, Burlington, MA). NMR experiments were performed on Bruker 400 MHz Avance NEO (5mm Prodigy Cryoprobe), Bruker 600 MHz Avance III HD (5 mm BBFO probe with Z gradient), Bruker 700 MHz Avance III HD Ascend (5mm TXO cryoprobe with Z-Gradients), Bruker 800 MHz Avance III HD Ascend (5mm TXI probe) and Bruker 850 MHz Avance III HD Ascend (5mm TCI cryoprobe with Z-Gradients). Matrix Assisted Laser Desorption Ionization (MALDI) mass spectra were obtained on a Bruker Microflex LRF instrument with 9-nitroanthacene or *trans*-2-[3-(4-*tert*-butylphenyl)-2-methyl-2-propenylidene]malononitrile (DCTB) as matrices. HRMS was performed on a Bruker Impact II QqTOF instrument in positive mode.

Syntheses of Basket 2 and Linker 4

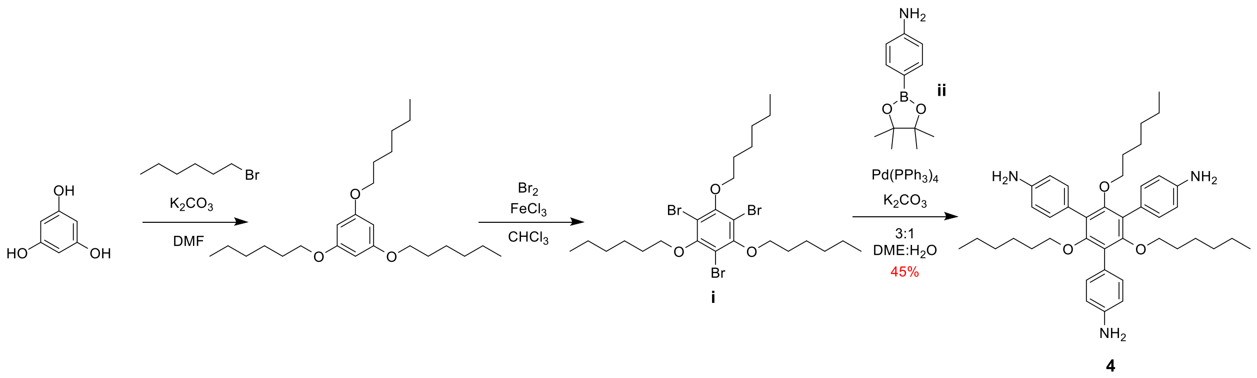

Scheme S1. Synthesis of *tris*-amine **4**. Compound **i** was prepared from phloroglucinol according to a procedure reported in the literature.[1]

***Tris*-amine 4:** To a flask equipped with a stirring bar, compound **i**[1] (500 mg, 0.813 mmol), compound **ii** (588, 2.68 mmol, 3.3eq) and potassium carbonate (1.35 g, 9.77 mmol, 12eq) were added. The flask was placed under an atmosphere of nitrogen and $Pd(PPh_3)_4$ (93.9 mg, 81.3 nmol, 0.10eq) was added in a glove box. 1,2-Dimethoxyethane (15 mL) and water (5 mL) were added. The flask was fitted with a reflux condenser and the reaction mixture was degassed by freeze-pump-thaw (5 times). The reaction was refluxed for 48h under an atmosphere of nitrogen. After cooling to r.t., the mixture was diluted with ethyl acetate (10 mL) and water (10 mL). Layers were separated and the aqueous layer was extracted with ethyl acetate (15 mL × 2). Combined organic layers were washed with brine (20 mL × 1) and dried over anhydrous sodium sulfate. After removing solvent under reduced pressure, the residue was purified by column chromatography (SiO_2, from neat CH_2Cl_2 to 20:1 = CH_2Cl_2:acetone), yielding *tris*-amine **4** as an orange solid (239 mg, 45%). ^1H NMR (400 MHz, CD_2Cl_2): δ (ppm) 7.20 (d, J = 8.6 Hz, 6H), 6.69 (d, J = 8.6 Hz, 6H), 3.69 (brs, 6H), 3.17 (t, J = 6.4 Hz, 6H), 1.57-1.03 (m, 12H), 0.98-0.84 (m, 12H), 0.80 (t, J = 7.3 Hz, 9H); ^{13}C NMR (100 MHz, CD_2Cl_2): δ (ppm) 155.4, 145.7, 132.1, 126.5, 125.2, 114.5, 73.1, 31.8, 30.1, 25.7, 22.9, 14.2. HRMS (ESI-MS): m/z calculated for $C_{42}H_{58}N_3O_3$: 652.4478 $[M+H]^+$; found 652.4472.

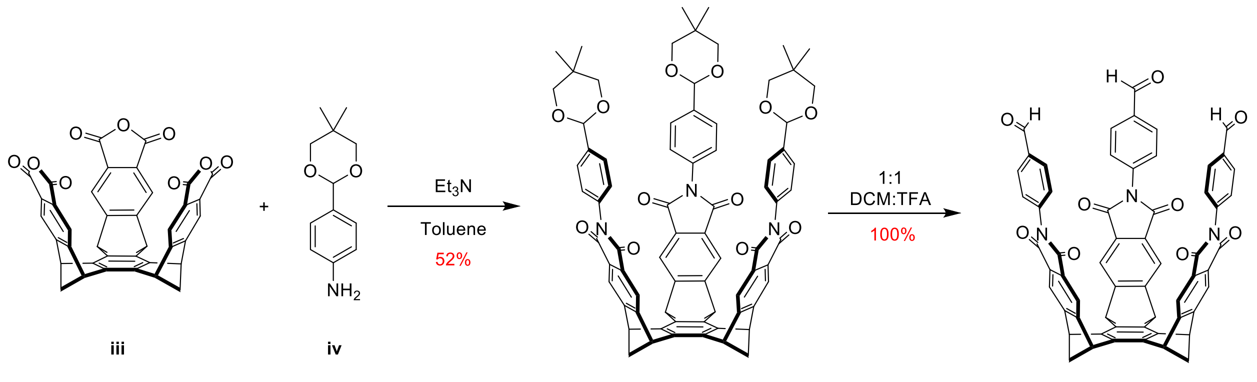

iii iv v 2

Scheme S2. Synthesis of basket **2** from *tris*-anhydride **iii**.[2–5]

Compound v: In a flask fitted with a Dean-Stark trap and reflux condenser, *tris*-anhydride **iii**[2–5] (150 mg, 0.238 mmol) and compound **iv**[6] (222 mg, 1.07 mmol, 4.5eq) were combined with toluene (75 mL). Triethylamine (667 µL, 481 mg, 4.76 mmol, 20eq) was added and the reaction was refluxed for 48h. After cooling to r.t., the solvent was removed under reduced pressure. The residue was purified by flash column chromatography by redissolving it in CH_2Cl_2 (20 mL) and then absorbing on to silica. This silica mixture was loaded on to a silica column packed with CH_2Cl_2. The product was eluted with 25:1 = CH_2Cl_2:acetone, gradually increasing polarity to 10:1 = CH_2Cl_2:acetone, affording compound **v** as a white solid (148 mg, 52%). ^{1}H NMR (850 MHz, $CDCl_3$): δ (ppm) 7.63 (s, 6H), 7.55 (d, J = 7.2 Hz, 6H), 7.29(d, J = 7.2 Hz, 6H), 5.38 (s, 3H), 4.58 (s, 6H), 3.74 and 3.62 (ABq, J = 10 Hz, 12H), 2.66 (s, 6H), 1.25 (s 9H), 0.79 (s, 9H); ^{13}C NMR (214 MHz, $CDCl_3$): δ (ppm) 167.0, 157.2, 138.1, 130.5, 126.9, 126.2, 116.5, 101.1, 77.7, 65.8, 49.3, 30.4, 23.2, 22.1. HRMS (ESI-MS): m/z calculated for $C_{75}H_{63}N_3NaO_{12}$: 1220.4309 $[M+Na]^+$; found 1220.4310.

***Tris*-aldehyde Basket 2:** Intermediate **v** (120 mg, 0.100 mmol) was dissolved in CH_2Cl_2 (30mL) and trifluoroacetic acid (30 mL). The mixture was stirred at r.t. and monitored by TLC. Once the reaction was complete (c.a. 12h), the solvent was removed under reduced pressure. The residue was suspended with diethyl ether (5 mL) and triethylamine (2 mL) was added to quench the residual acid. The mixture was centrifuged, and the supernatant was discarded. The resulting residue was triturated with methanol (5

mL × 3) and dried under vacuum to give *tris*-aldehyde basket **2** as a white solid (93.6 mg, 100%). ^{1}H NMR (850 MHz, CD_2Cl_2): δ (ppm) 9.99 (s, 3H), 7.92 (d, J = 4.0 Hz, 6H), 7.72 (s, 6H), 7.55 (d, J = 4.0, 6H), 4.66 (s, 6H), 2.69 (m, 6H); ^{13}C NMR (214 MHz, CD_2Cl_2): δ (ppm) 191.5, 166.9, 158.4, 138.5, 137.6, 135.5, 130.5, 130.4, 126.7, 116.9, 66.5, 49.7. HRMS (ESI-MS): m/z calculated for $C_{60}H_{33}N_3NaO_9$: 962.2114 $[M+Na]^+$; found: 962.2115.

Probing the Synthesis of [4+4] Cage 1

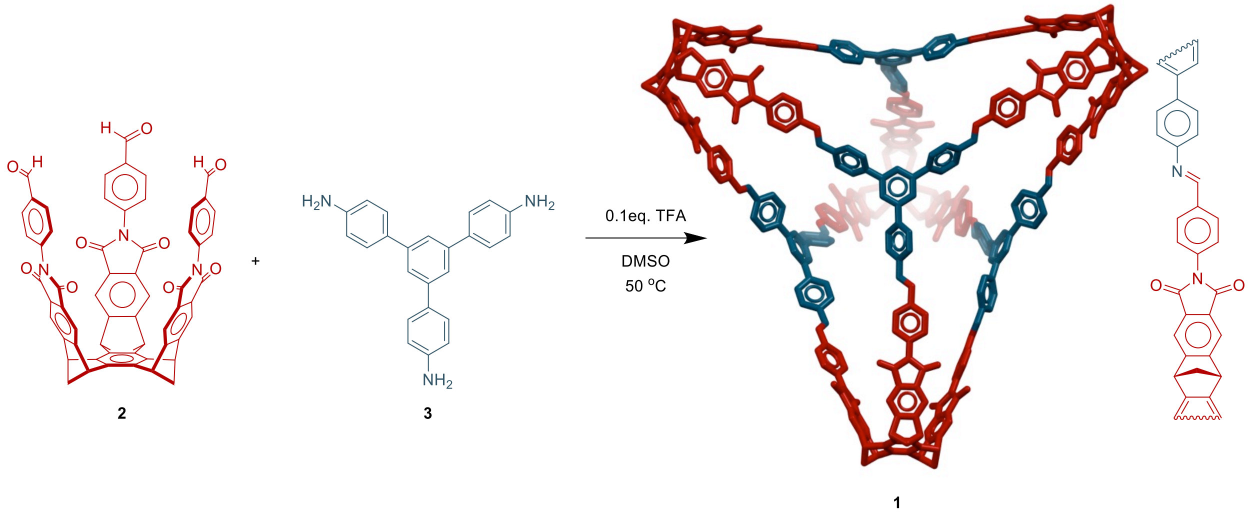

Scheme S3. Synthesis of [4+4] cage **1** with *tris*-aldehyde **2** and *tris*-amine **3**.

Tris-aldehyde **2** (2.02 mg, 2.15 µmol) and *tris*-amine **3**[7] (1.56 mg, 4.44 µmol, 2.1 eq) were dissolved in DMSO (1 mL). Trifluoroacetic acid solution in DMSO (29.8 mM, 7.2 µL, 0.21 µmol, 0.1 eq) was added, and the reaction was heated at 50 °C for 12h. The reaction mixture was analyzed with MALDI (Figure S1): 1 µL of sample mixed with 1 µL of saturated matrix solution (9-nitroanthracene) in acetonitrile and spotted on MALDI plate. Measurements were done in linear positive mode (Figure S1).

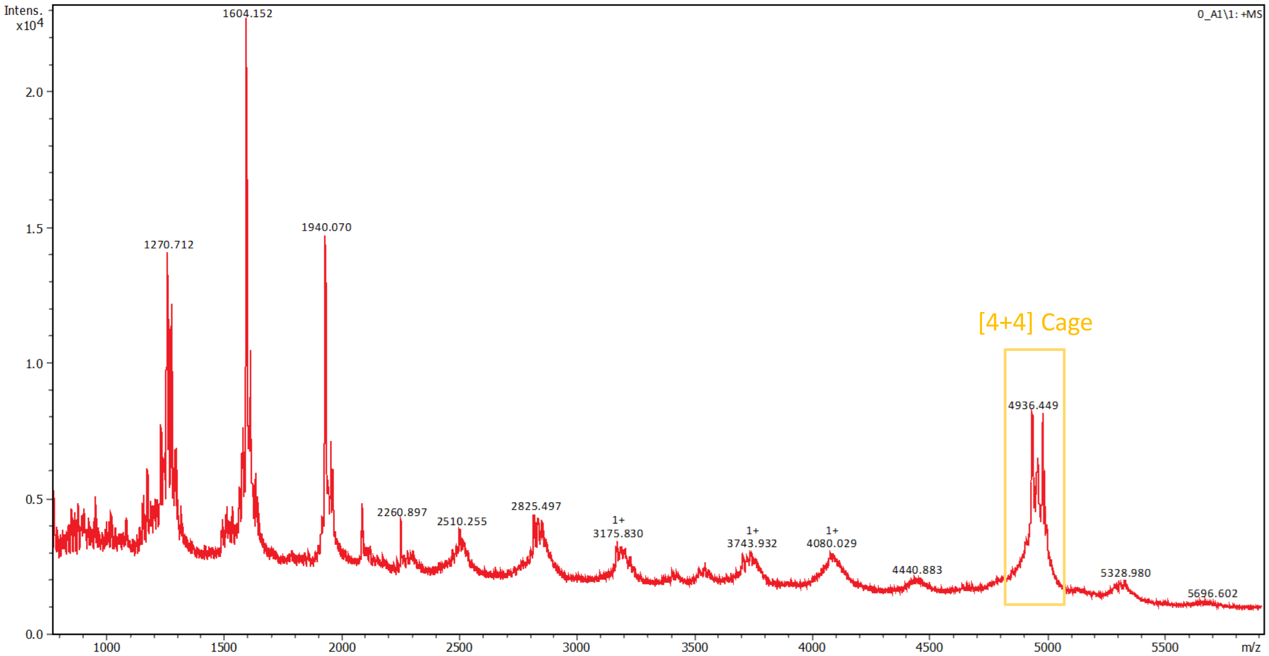

Figure S1. MALDI spectra (9-nitroanthracene as matrix; linear positive mode) obtained from the TFA catalyzed reaction between *tris*-aldehyde **2** and *tris*-amine **3** in DMSO. Yellow box shows a region corresponding to the formation of [4+4] cage **1**.

Solvent Screen for Synthesis of [4+4] Cage 1

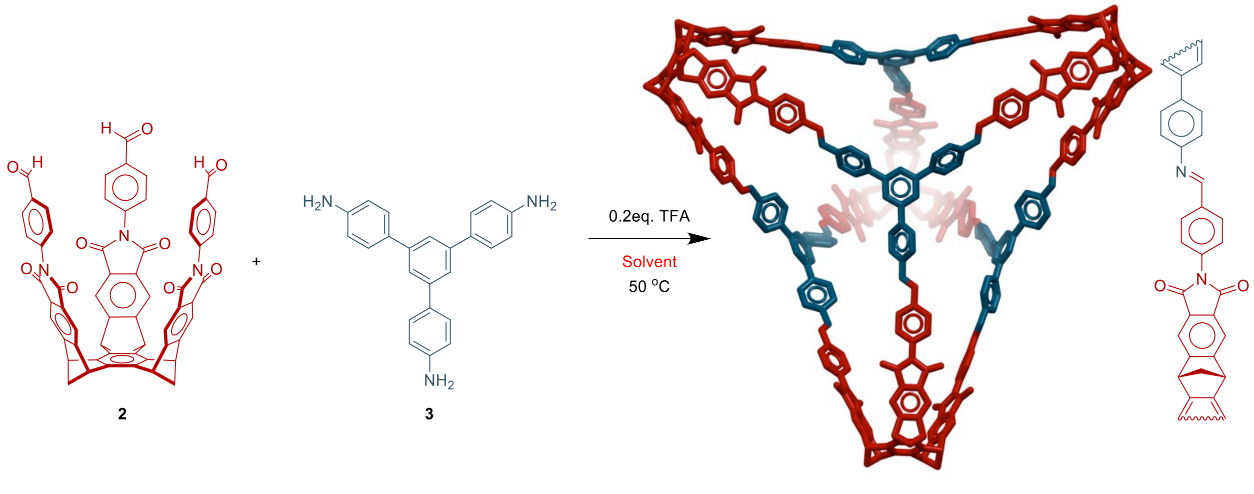

Scheme S4. Solvent screen for the synthesis [4+4] cage **1**.

General procedure: *Tris*-aldehyde **2** (0.53 µmol) was combined with *tris*-amine **3** (1.13 µmol, 2.1eq) in 1 mL of solvent listed in Figure S2. Trifluoroacetic acid (0.11 µmol, 0.20 eq) was added and the reaction was

stirred at 50 °C for 20h. The reaction mixtures were analyzed with MALDI (Figure S2): 1 µL of sample mixed with 1 uL of saturated matrix solution (9-nitroanthracene) in acetonitrile and spotted on MALDI plate. Measurements were done in linear positive mode.

Results and solubility of cage 1: MALDI measurements showed that reactions with chloroform, tetrahydrofuran (THF) and 1,2-dichloroethane (DCE) as solvent gave the cage as the primary species (Figure S2). We disregarded THF as a potential solvent for its propensity to polymerize under acidic conditions. Chloroform, on the other hand, tended to contain small amounts of acid which gave inconsistent results. Therefore, DCE was chosen as the best solvent to synthesize the cage compounds. While the cage **1** was detected by MALDI-MS, we were not able to characterize the cage by NMR due to its poor solubility in common organic solvents. As we outlined in the main text, we sought to improve the solubility of the cage by synthesizing linker **4**.

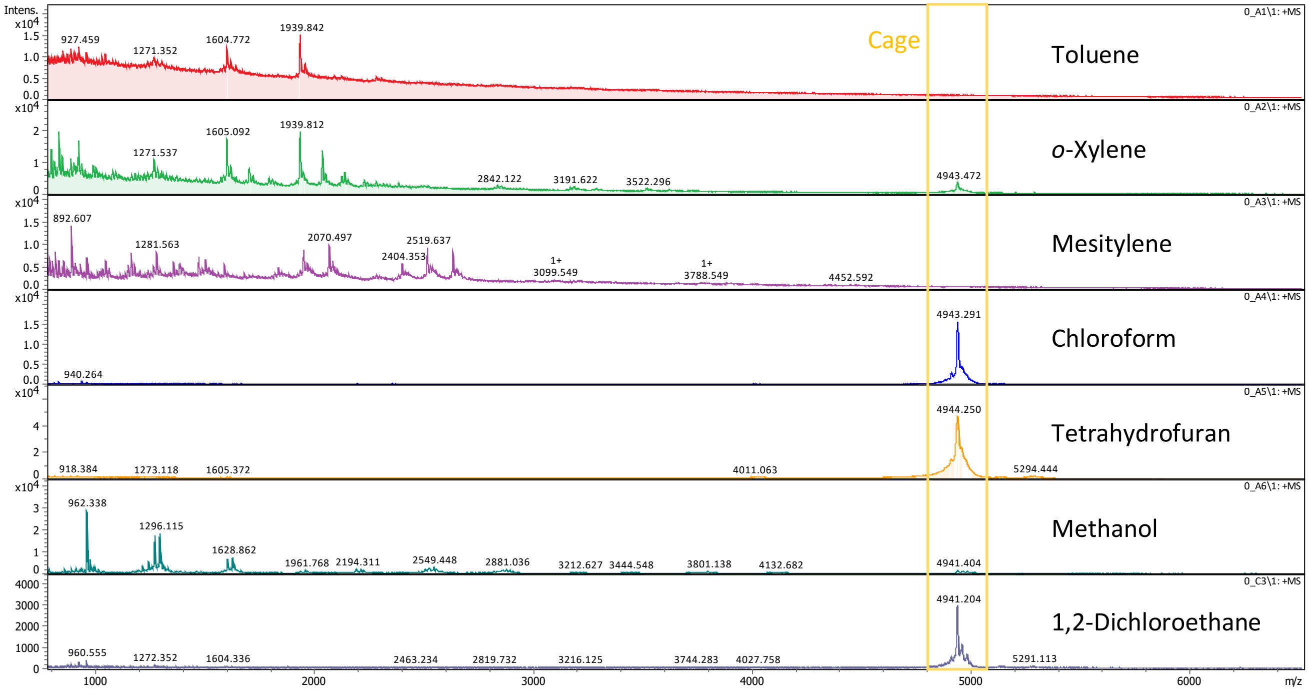

Figure S2. MALDI spectra (9-nitroanthracene as matrix; linear positive mode) obtained from screening different solvents in the synthesis of **1**. Yellow box shows the region corresponding to the formation of [4+4] cage **1**.

Synthesis of CBC 5 and [1+1] Capsule 6

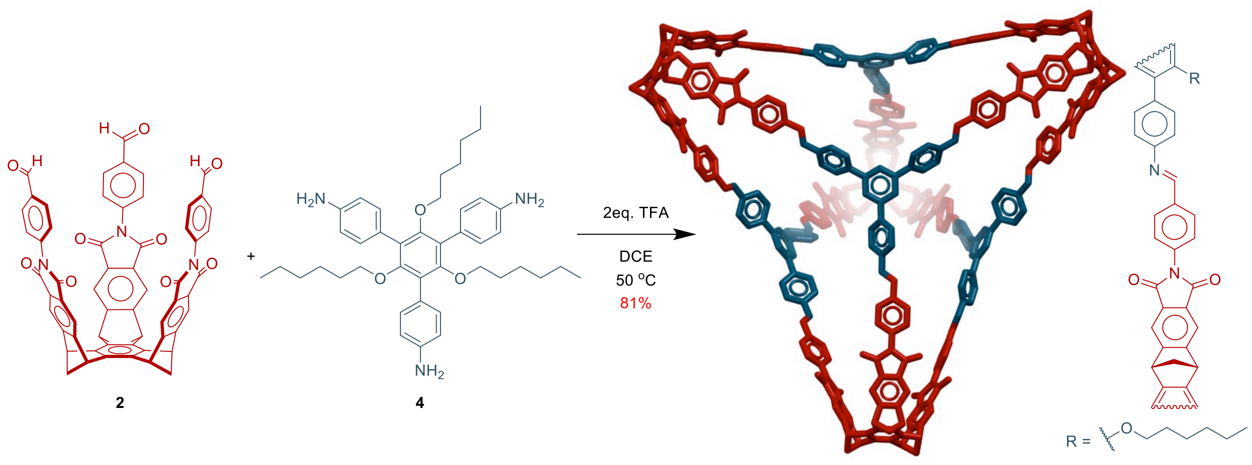

Scheme S5. Acid-catalyzed synthesis of CBC **5**.

CBC 5: In a round bottom flask, *tris*-aldehyde **2** (20.0 mg, 21.3 µmol) and *tris*-amine **4** (15.0 mg, 23.0 µmol, 1.1eq) were dissolved in 1,2-dichloroethane (43 mL, 0.50 mM with respect to **2**). Trifluoroacetic acid (4.85 mg, 42.6 µmol, 2.0 eq) in 1,2-dichloroethane (0.2 mL) was added. The reaction was sealed and heated at 50 ^{o}C with stirring for 20h. Triethylamine (50 µL) was added to quench the reaction and solvent was removed under reduced pressure. The residue was washed by suspending it in methanol (5 mL) followed by sonication. The solid was isolated by centrifugation. This washing procedure was repeated with 5 mL of diethyl ether followed by 5 mL of CH_2Cl_2:CH_3OH = 1:1. CBC **5** was isolated after drying under vacuum as a yellow solid (26.6 mg, 81%). ^{1}H NMR (850 MHz, CD_2Cl_2): δ (ppm) 8.59 (s, 12H), 7.99 (d, J = 8.5 Hz, 24H), 7.72 (s, 24H), 7.51 (d, J = 8.5 Hz, 24H), 7.46 (d, J = 8.5 Hz, 24H), 7.31 (d, J = 8.5 Hz, 24H), 4.66 (s, 24H), 3.22 (t, J = 6.1 Hz, 24H), 2.70 (m, 24H), 1.08-1.00 (m, 48H), 0.90-0.84 (m, 24H), 0.84-0.78 (m, 24H), 0.71 (t, J = 7.3 Hz, 36H); ^{13}C NMR (176 MHz, CD_2Cl_2): δ (ppm) 167.3, 158.5, 158.2, 155.8, 150.3, 138.6, 136.2, 134.6, 133.6, 132.1, 130.6, 129.4, 127.1, 126.4, 120.8, 116.8, 73.6, 66.2, 49.7, 31.7, 30.0, 25.6, 22.9, 14.2. MALDI-

TOF (DCTB, reflector mode): m/z calculated for $C_{408}H_{337}N_{24}O_{36}$ $[M+H]^+$: 6147.53; found: 6151.16 (no isotopic resolution observed; highest point of peak reported, see Figure S11).

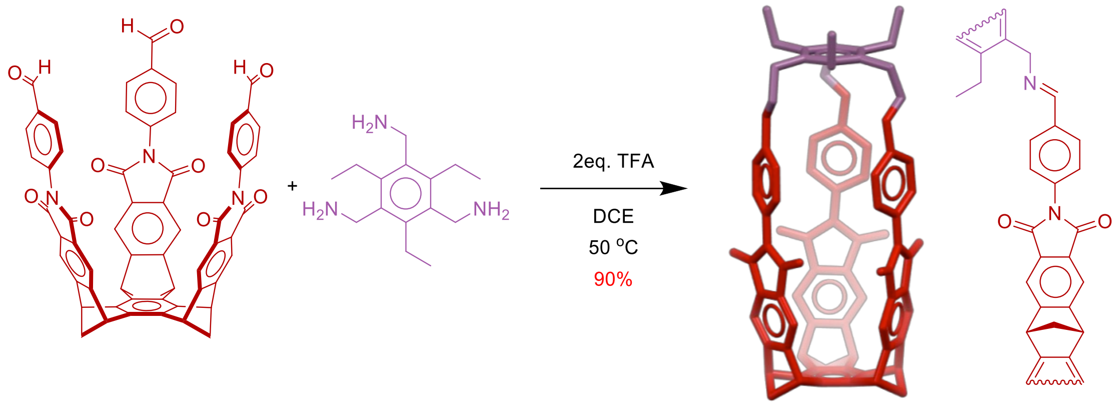

Scheme S6. Acid-catalyzed synthesis of [1+1] capsule **6**.

Capsule 6: In a round bottom flask, *tris*-aldehyde **2** (15.6 mg, 16.6 μmol) and *tris*-amine **7**[8] (8.5 mg, 34.1 μmol, 2.1 eq) were dissolved in 1,2-dichloroethane (33 mL, 0.50 mM with respect to **2**). Trifluoroacetic acid (3.6 mg, 32 μmol, 1.9 eq) dissolved in 1,2-dichloroethane (0.2 mL) was added. The reaction was sealed and stirred at 50 °C for 24h. Triethylamine (50 μL) was added to quench the reaction and solvent was removed under reduced pressure. The residue was washed by suspending it in 5 mL of methanol followed by sonication. The solid was isolated by centrifugation. This washing procedure was repeated with additional methanol (2 x 3mL). After drying under vacuum, [1+1] capsule **6** was isolated as a white solid (16.9 mg, 90%). ^1H NMR (850 MHz, CD_2Cl_2): δ (ppm) 7.52 (s, 6H), 7.40 (s, 3H), 7.08 (d, J = 7.8 Hz, 6H), 6.82 (d, J = 7.8 Hz, 6H), 5.04 (s, 6H), 4.57 (s, 6H), 2.71 (m, 6H), 2.56 (q, 7.4 Hz, 6H), 1.26 (t, J = 7.4 Hz, 9H); ^{13}C NMR (214 MHz, CD_2Cl_2): δ (ppm) 166.9, 160.6, 158.7, 144.4, 141.0, 136.3, 134.3, 133.4, 130.4, 129.4, 127.1, 117.0, 63.2, 55.4, 49.9, 24.2, 17.0. MALDI-TOF (DCTB, reflector mode): m/z calculated for $C_{75}H_{55}N_6O_6$ $[M+H]^+$: 1135.42; found: 1135.50 (see Figure S16).

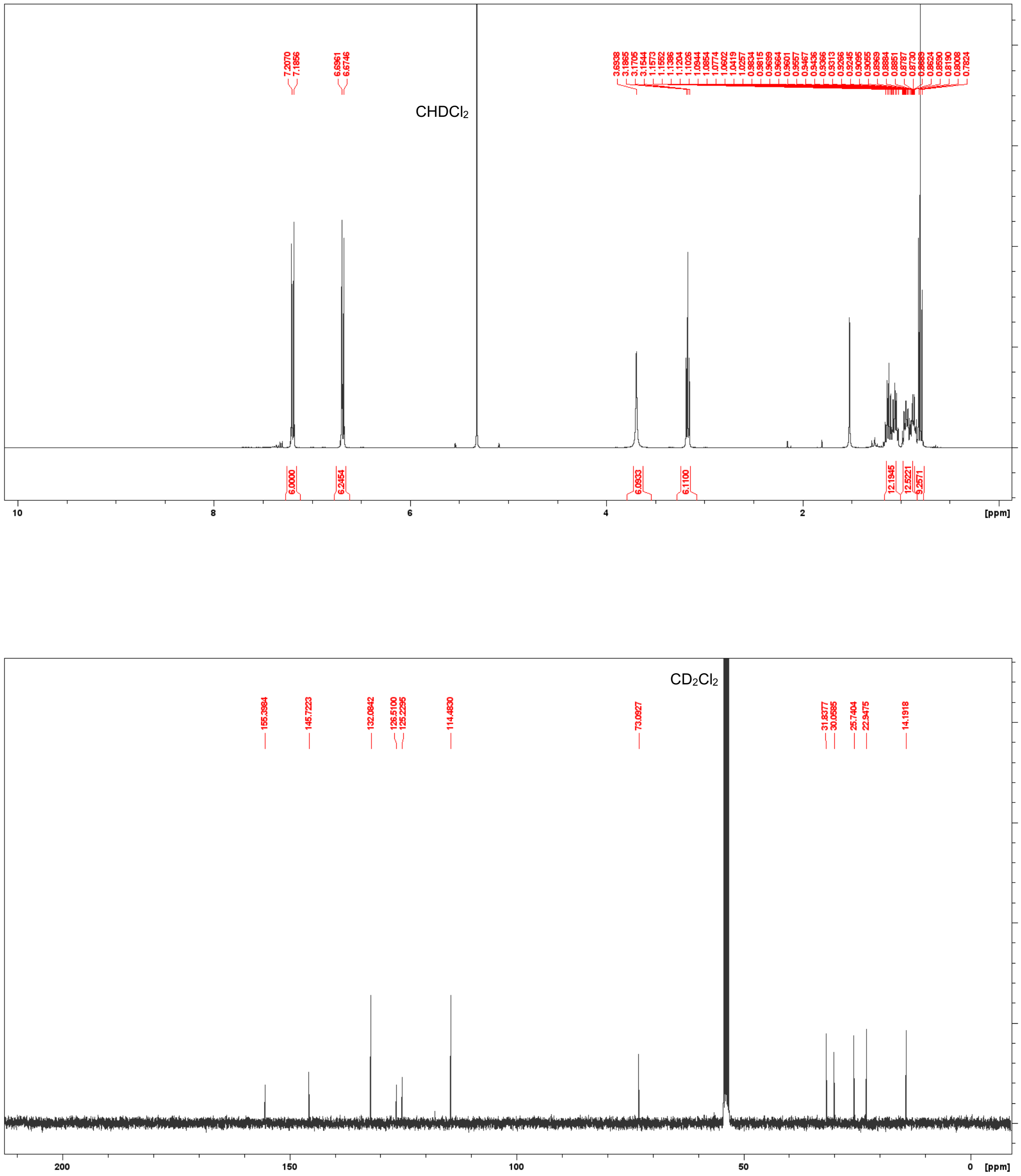

Figure S3. ^{1}H NMR (top: 400 MHz, 298K, CD_2Cl_2) and $^{13}C\{^{1}H\}$ NMR (bottom: 150 MHz, 298K, CD_2Cl_2) spectra of *tris*-amine **4**.

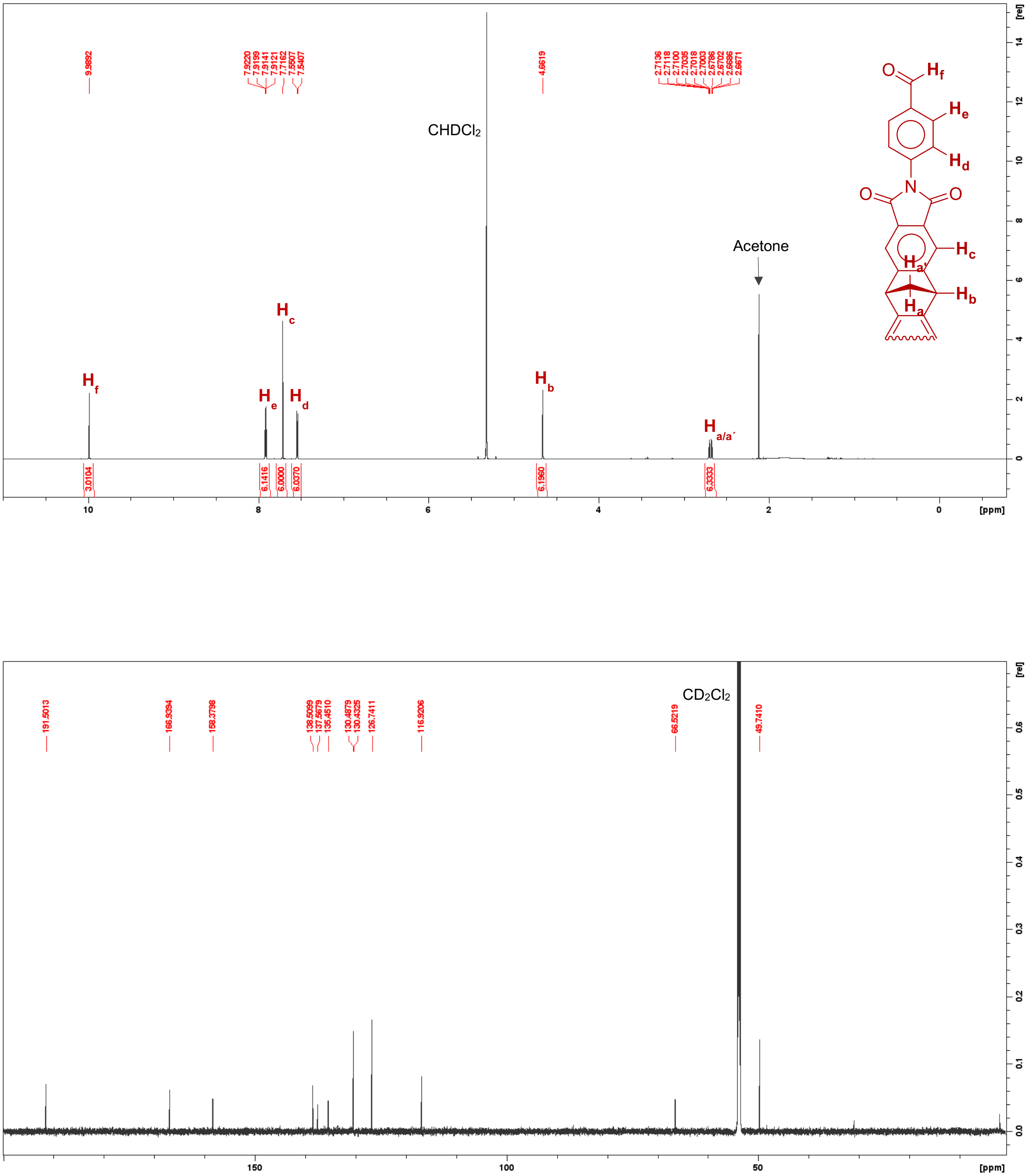

Figure S4. ^{1}H NMR (top: 850 MHz, 298K, CD_2Cl_2) and $^{13}C\{^{1}H\}$ NMR (bottom: 214 MHz, 298K, CD_2Cl_2) spectra of *tris*-aldehyde **2**.

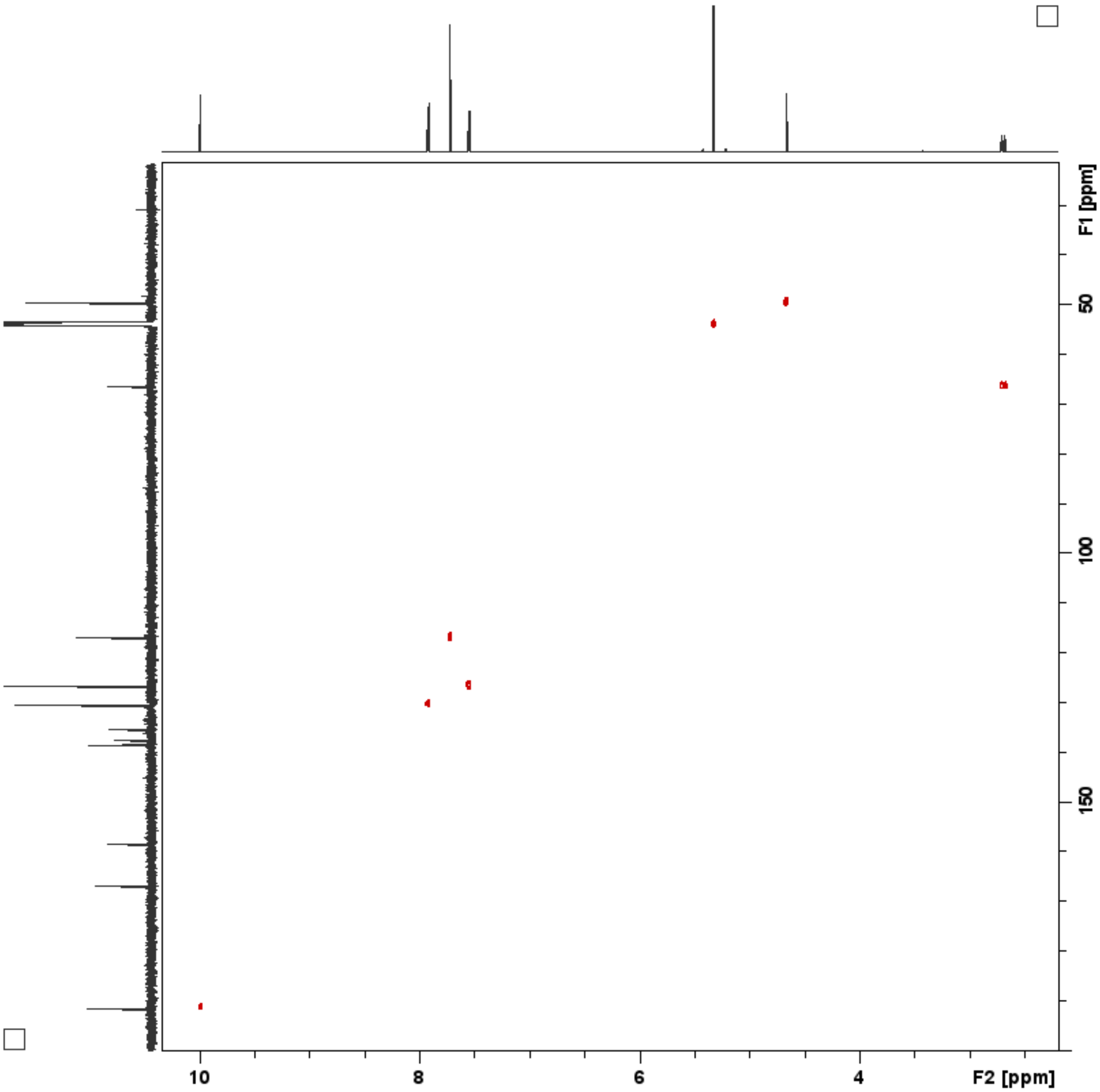

Figure S5. ^{1}H-^{13}C HSQC spectrum of *tris*-aldehyde **2** (850 MHz, 298K, CD_2Cl_2).

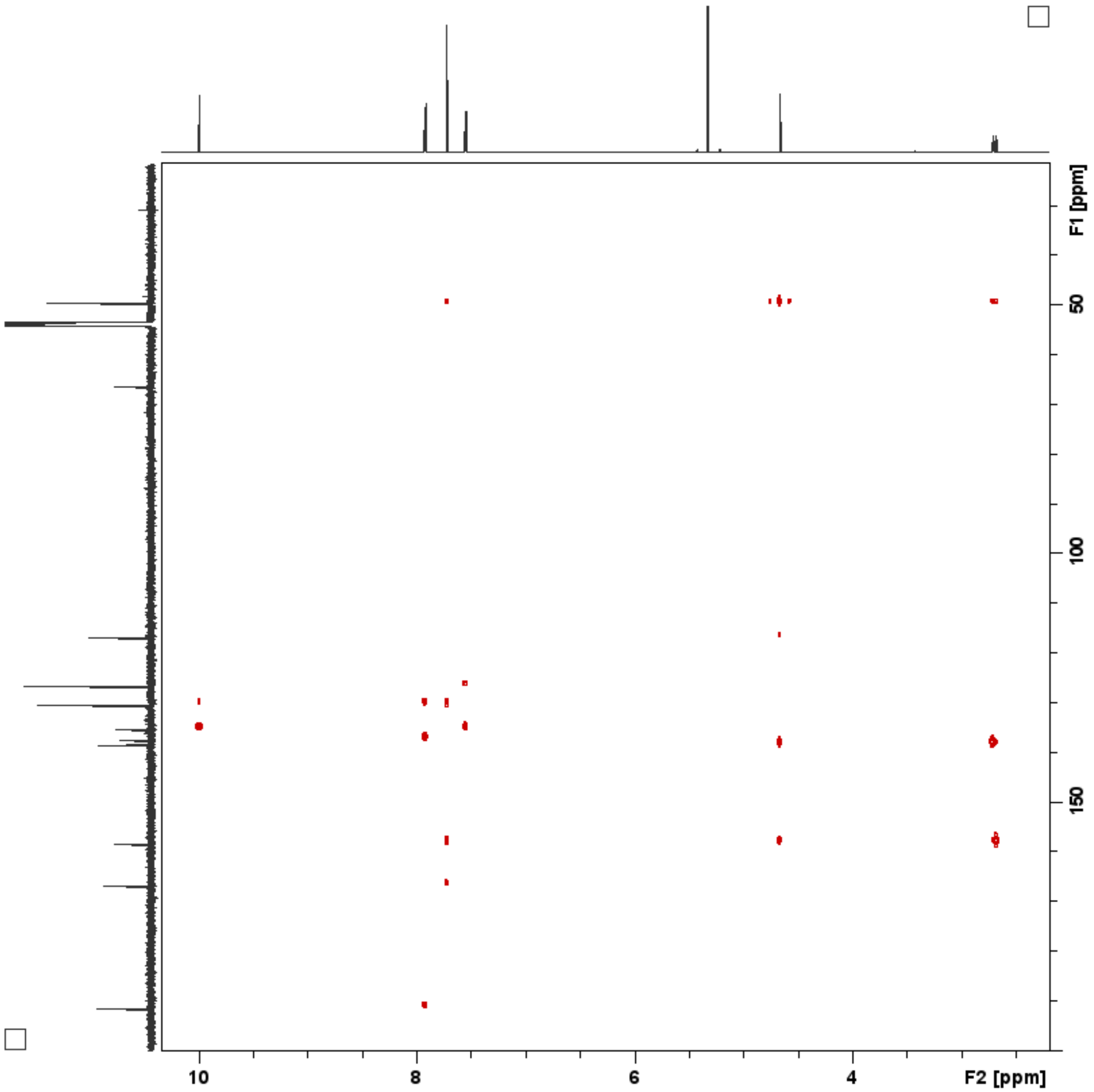

Figure S6. ^{1}H-^{13}C HMBC spectrum of *tris*-aldehyde **2** (850 MHz, 298K, CD_2Cl_2).

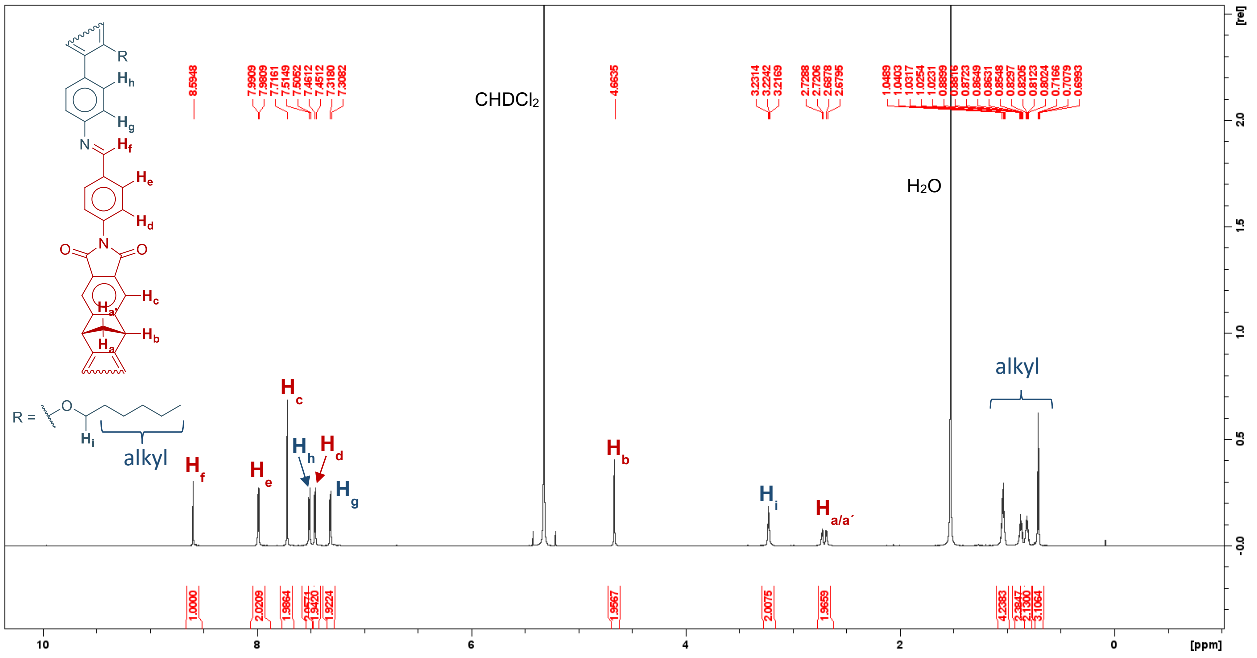

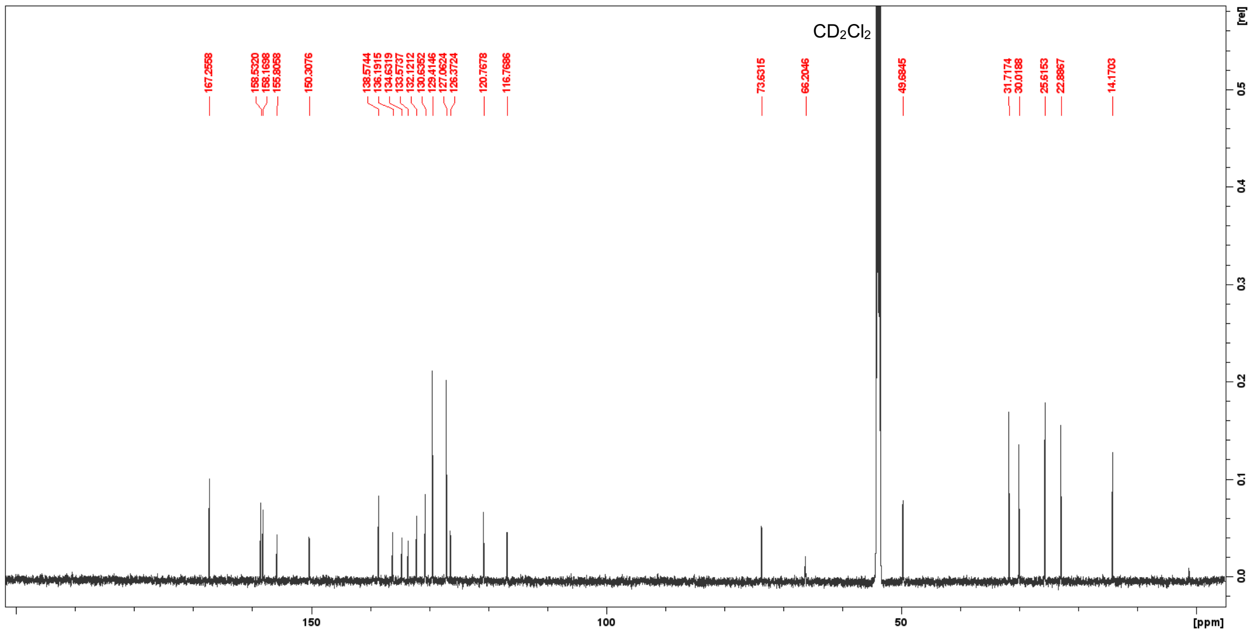

Figure S7. ^{1}H NMR (top: 850 MHz, 298K, CD_2Cl_2) and $^{13}C\{^{1}H\}$ NMR spectra of CBC **5** (bottom: 176 MHz, 298K, CD_2Cl_2).

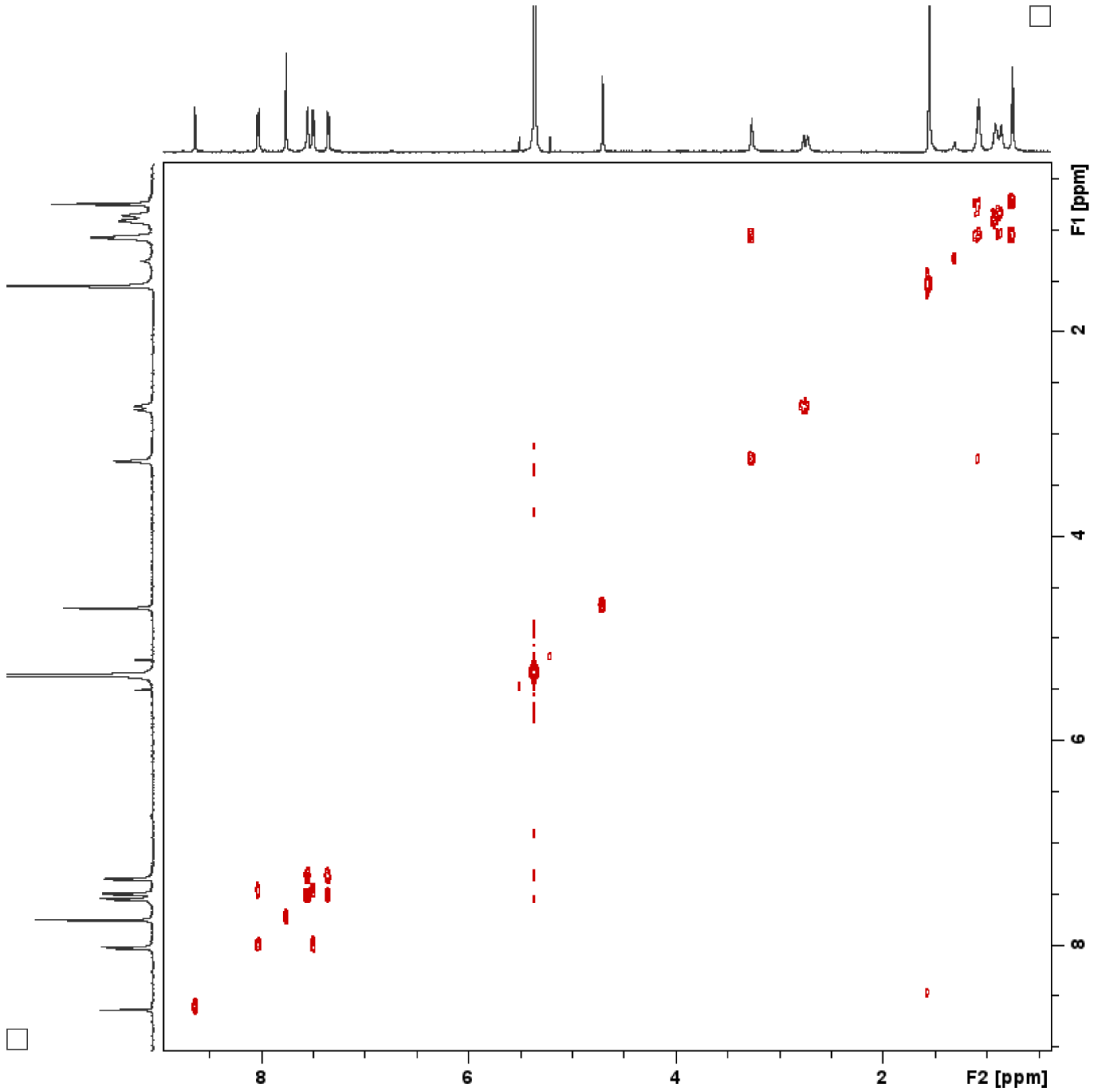

Figure S8. ^1H-^1H COSY spectrum of CBC **5** (850 MHz, 298K, CD_2Cl_2).

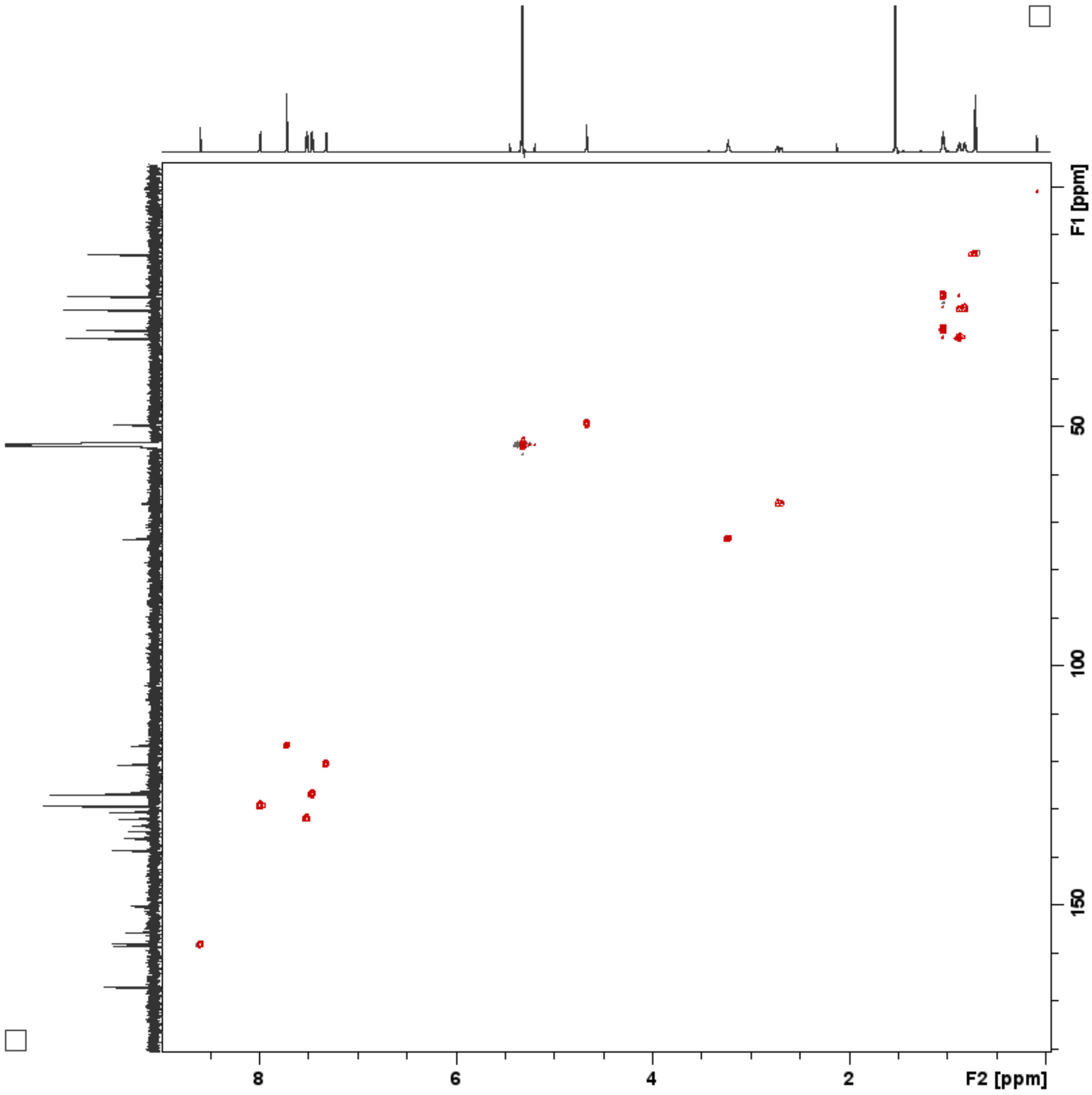

Figure S9. ^{1}H-^{13}C HSQC spectrum of CBC **5** (850 MHz, 298K, CD_2Cl_2).

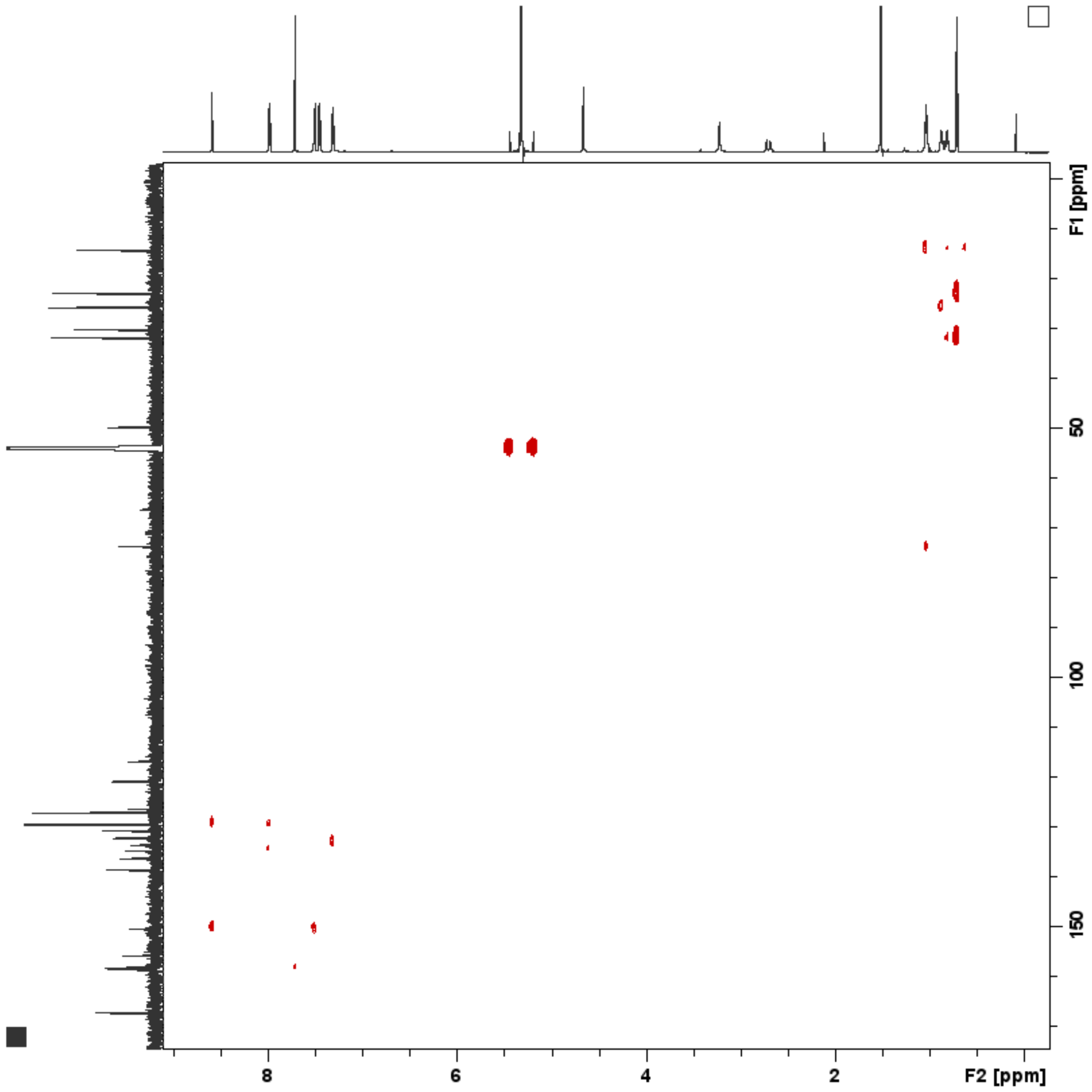

Figure S10. ^1H-^{13}C HMBC spectrum of CBC **5** (850 MHz, 298K, CD_2Cl_2).

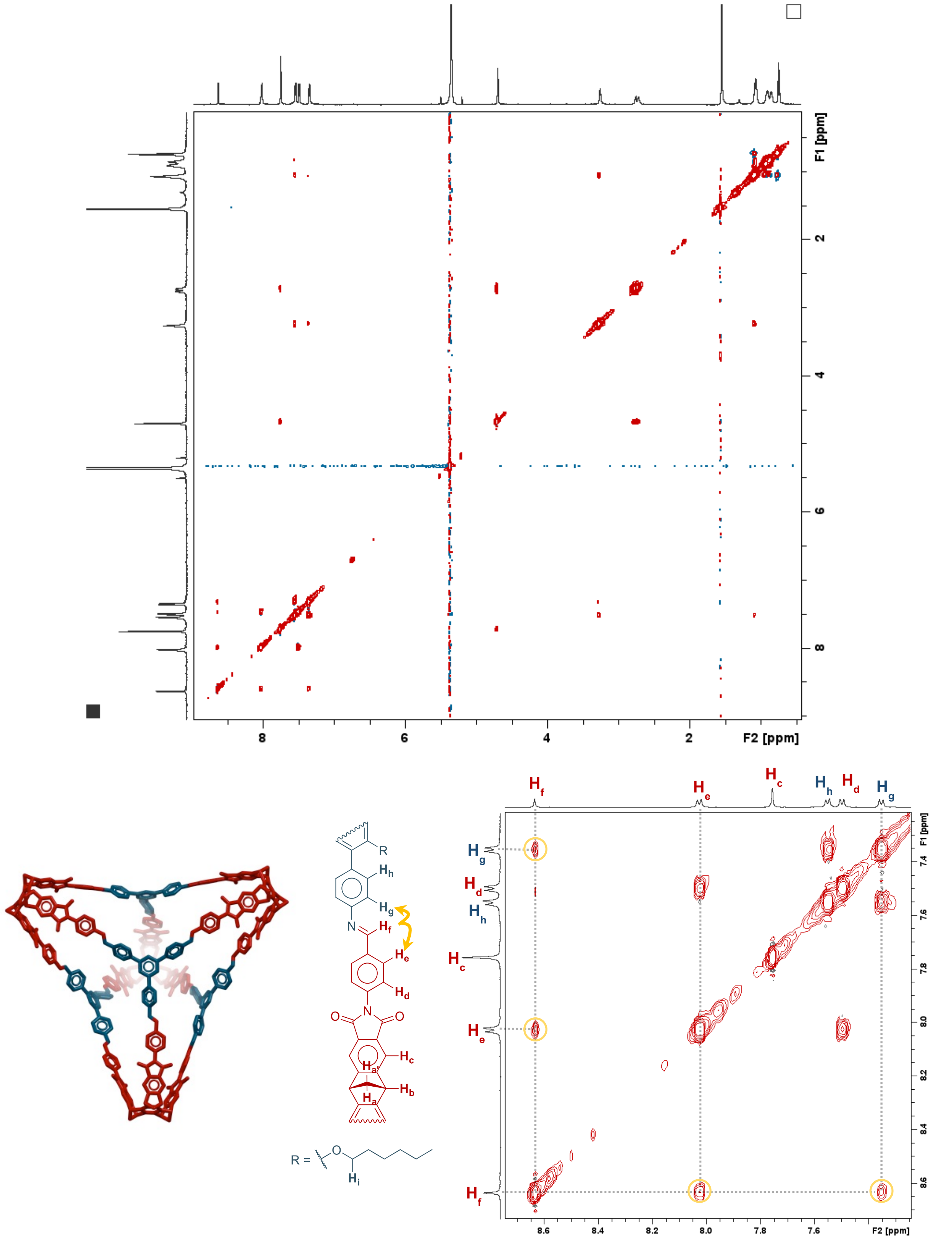

Figure S11. ^{1}H-^{1}H NOESY spectrum of CBC **5** (top: 600 MHz, 300K, CD_2Cl_2) with highlighted (bottom) NOE cross-peaks between the basket and the linker portions of the cage.

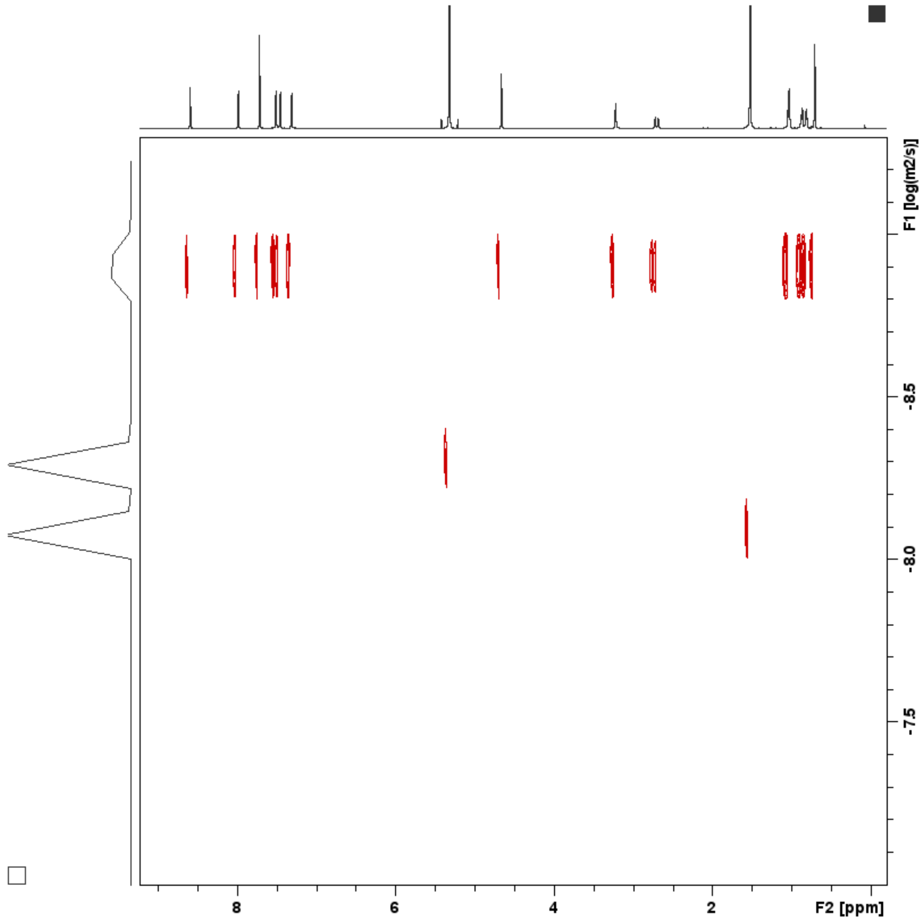

Figure S12. ^{1}H DOSY NMR spectrum (850 MHz, 298K) of CBC **5** in CD_2Cl_2.

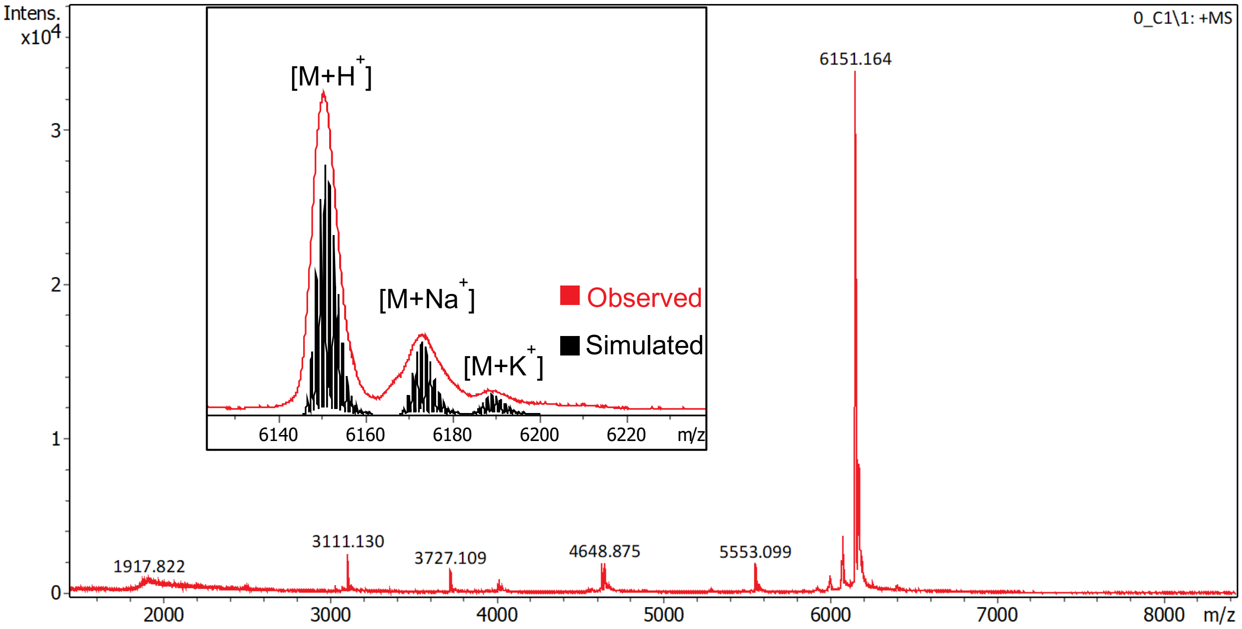

Figure S13. MALDI-MS spectrum of CBC **5** (matrix: DCTB; positive reflector mode). Inset shows zoomed-in view of the peaks corresponding to the cage (red) with overlaid simulated isotopic pattern (black).

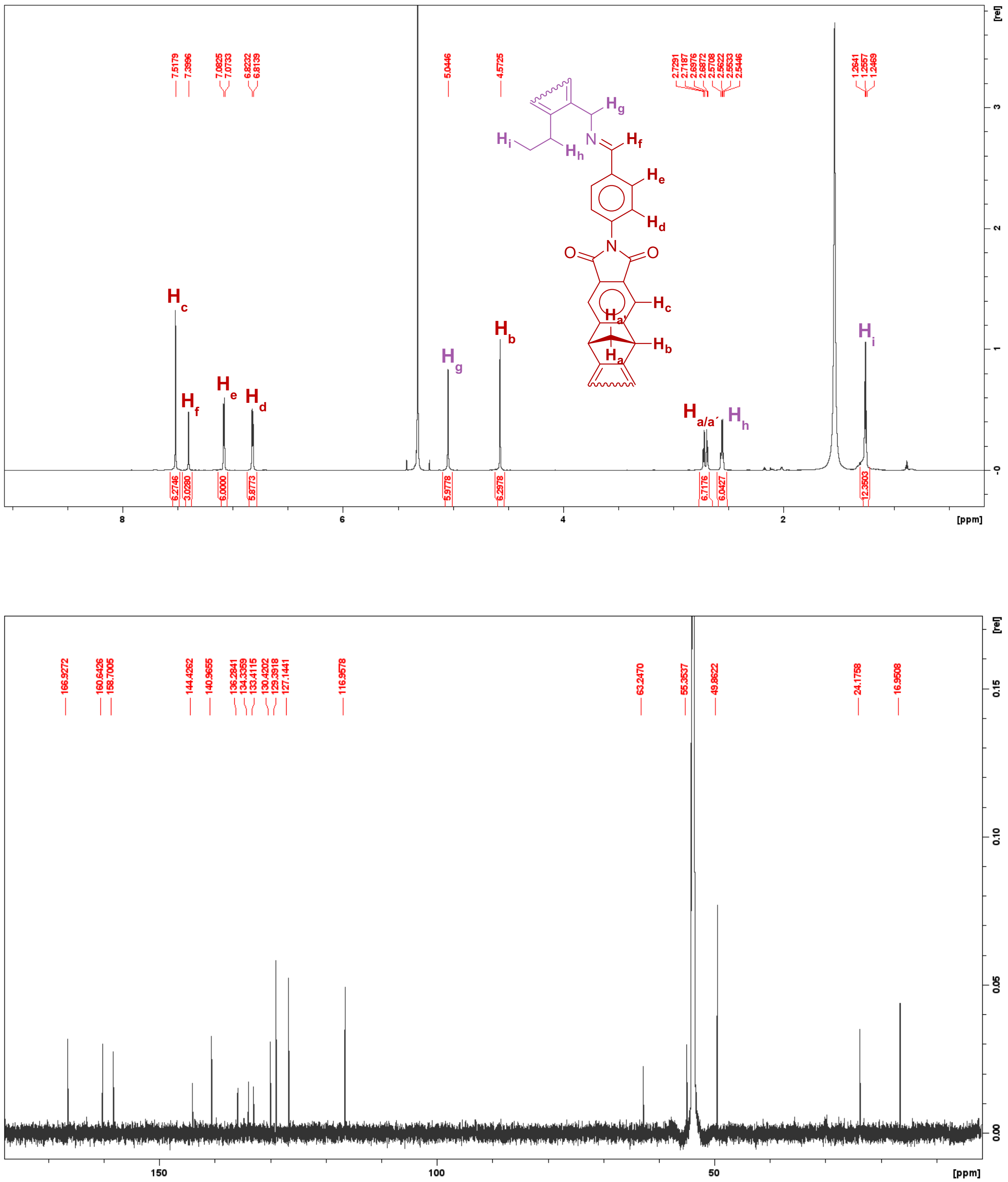

Figure S14. ^{1}H NMR (top: 850 MHz, 298K, CD_2Cl_2) and $^{13}C\{^1H\}$ NMR (bottom: 214 MHz, 298K, CD_2Cl_2) spectra of [1+1] capsule **6**.

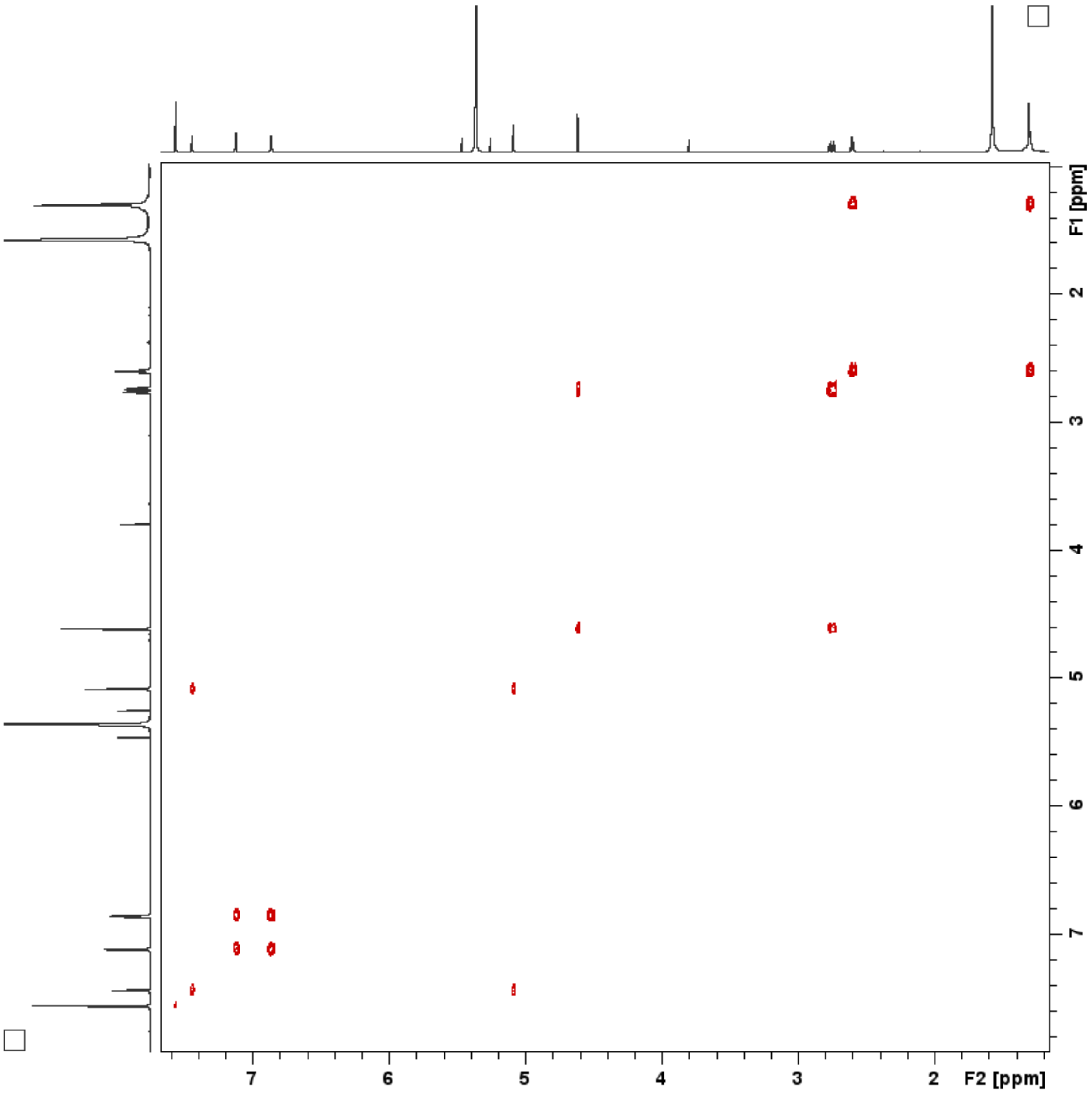

Figure S15. ^{1}H-^{1}H COSY spectrum of [1+1] capsule **6** (850 MHz, 298K, CD_2Cl_2).

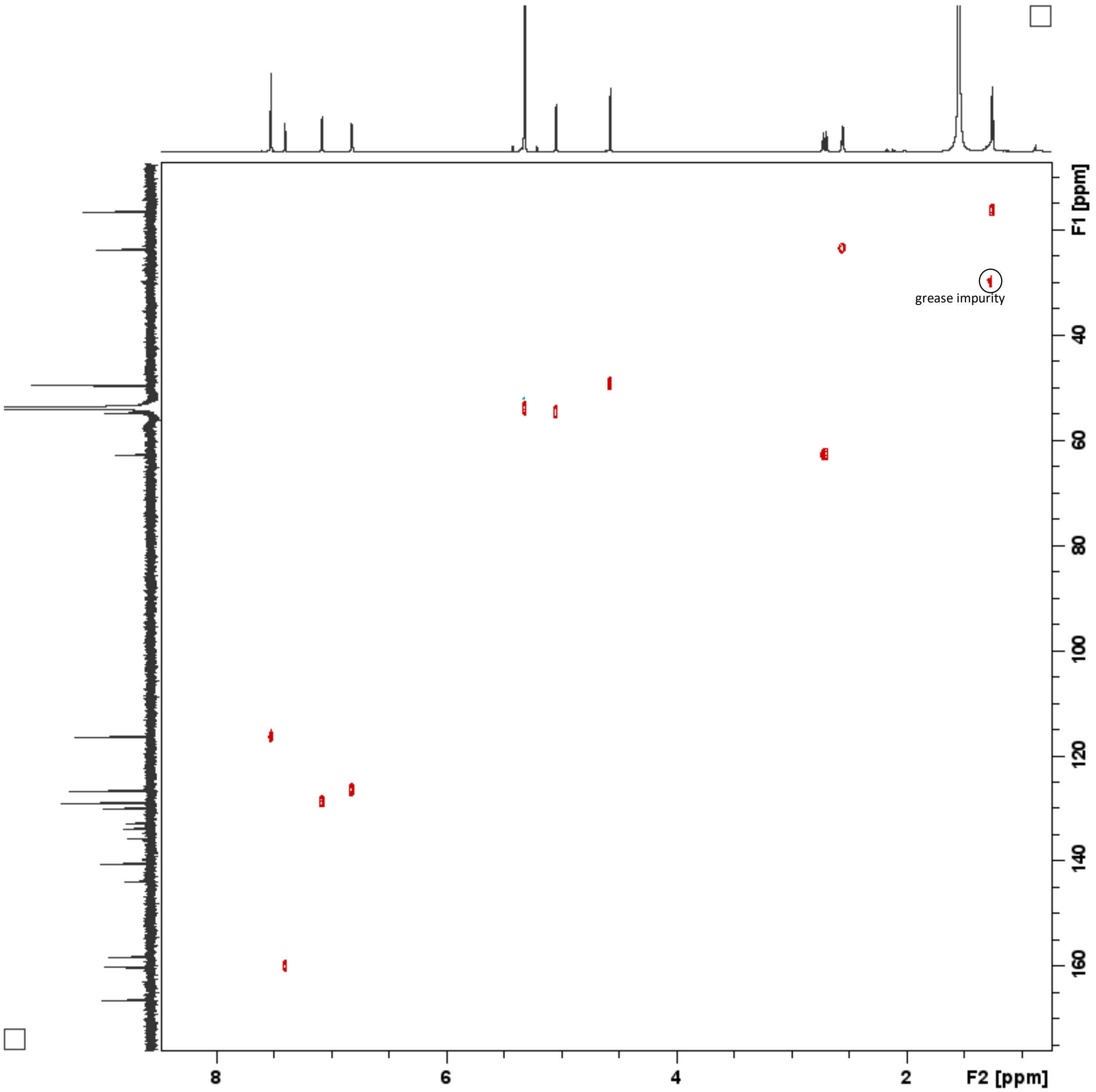

Figure S16. ^{1}H-^{13}C HSQC spectrum of [1+1] capsule **6** (850 MHz, 298K, CD_2Cl_2).

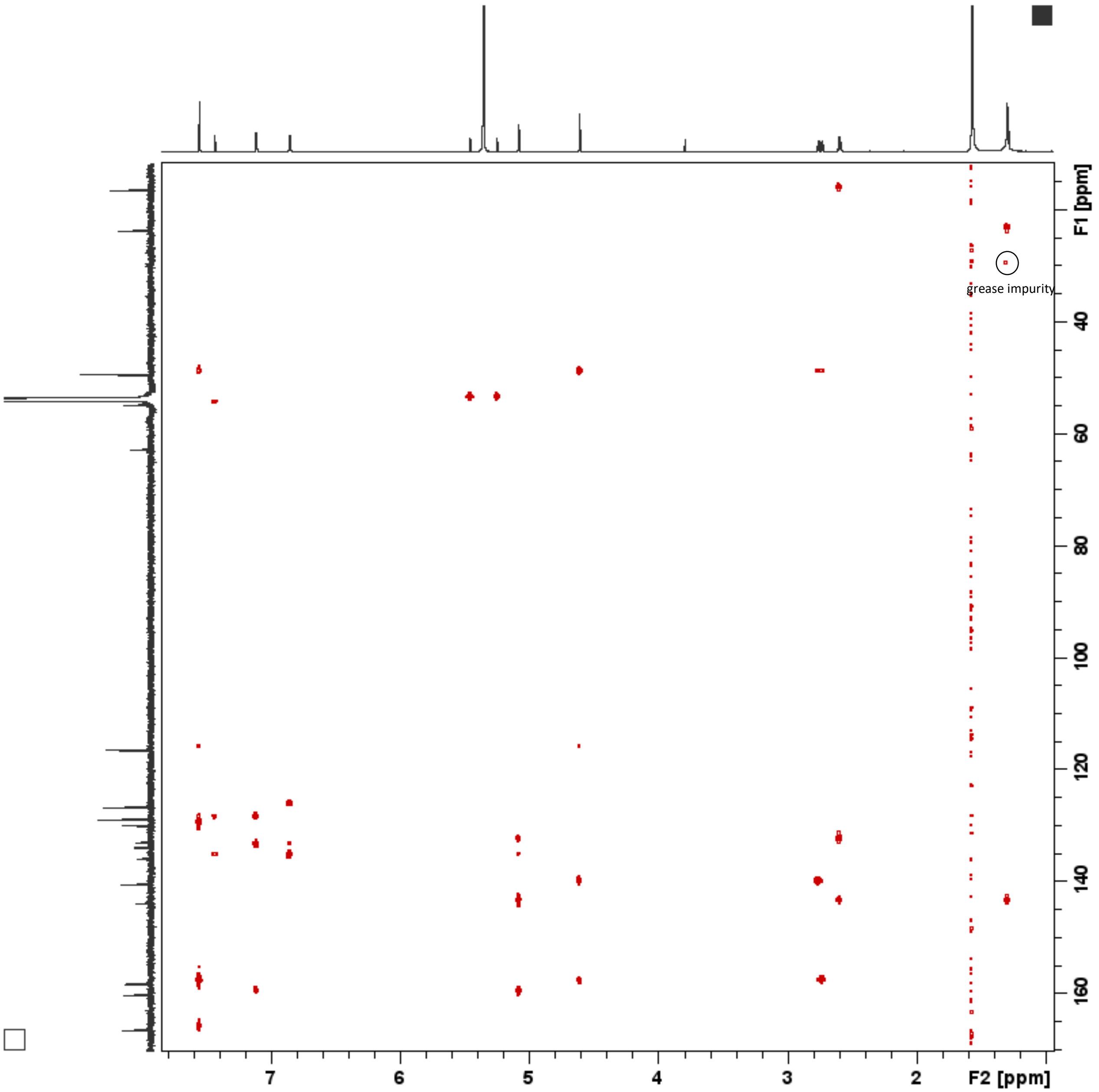

Figure S17. ^{1}H-^{13}C HMBC (850 MHz, 298K) spectrum of [1+1] capsule **6** in CD_2Cl_2.

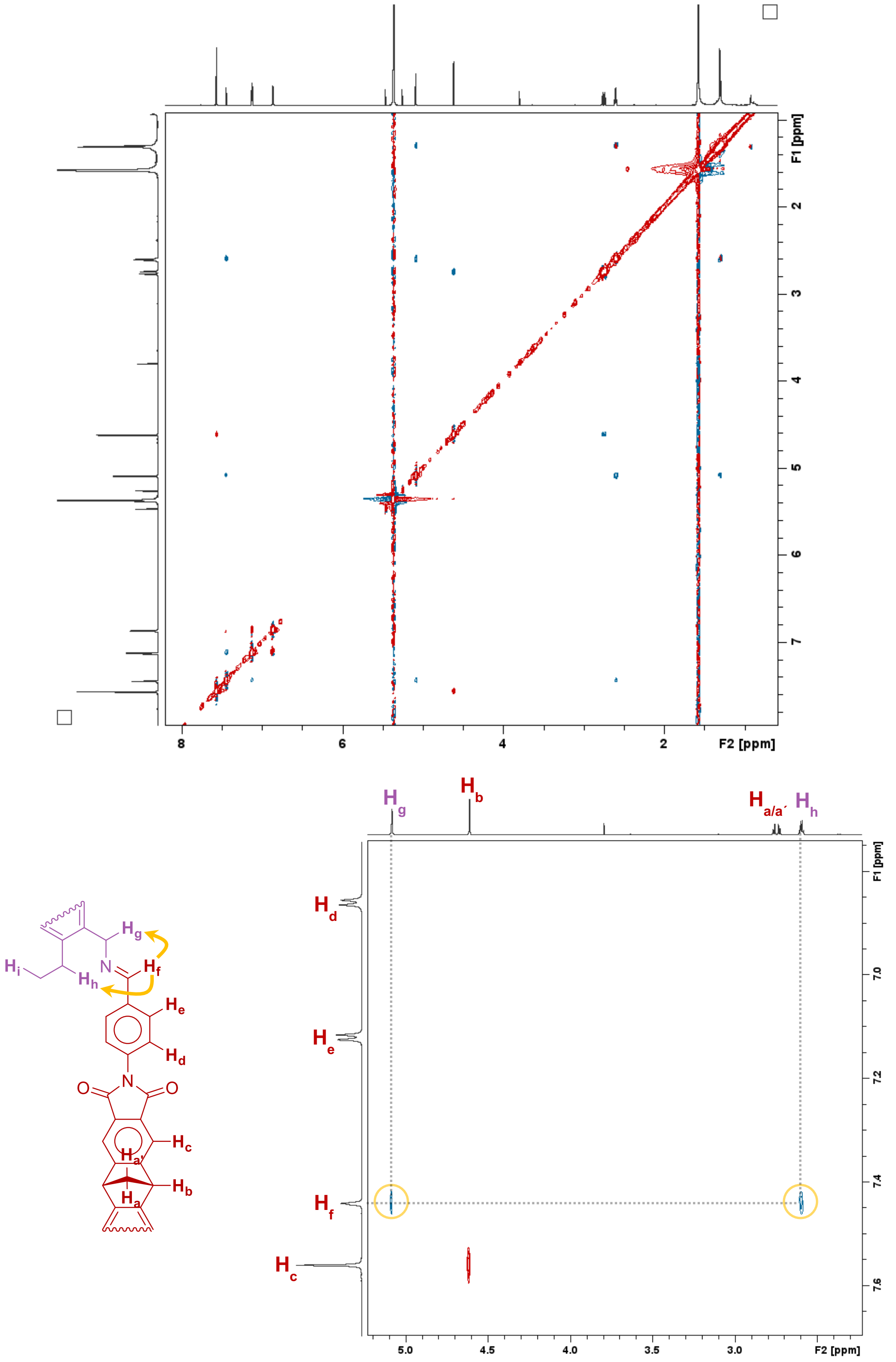

Figure S18. ^{1}H-^{1}H NOESY spectrum of [1+1] capsule **6** (top: 850 MHz, 298K, CD_2Cl_2) with highlighted NOE correlations (bottom) between the basket and the linker portions of the cage.

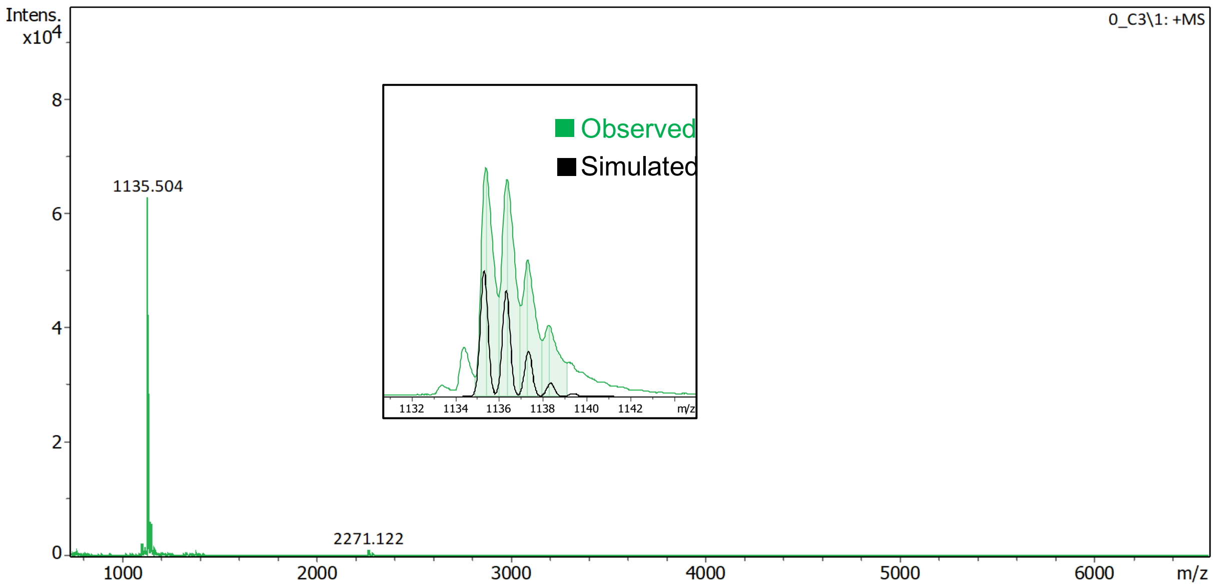

Figure S19. MALDI-MS spectrum of [1+1] capsule **6** (matrix: DCTB; positive reflector mode). Inset shows zoomed-in view of the peaks corresponding to the capsule (green) with overlaid simulated isotopic pattern (black).

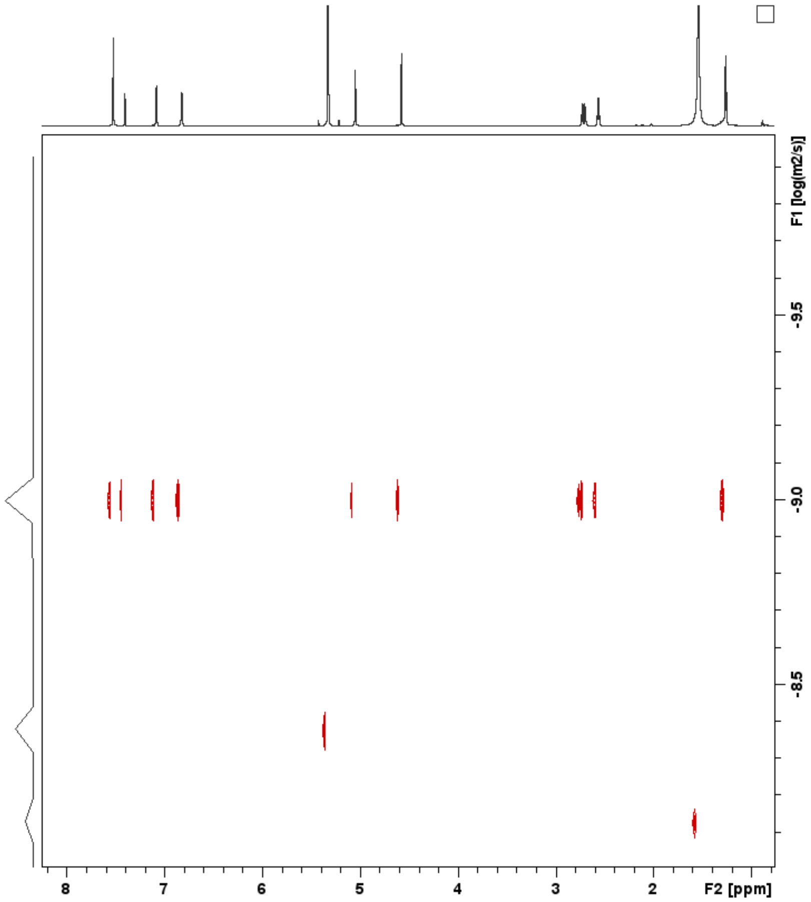

Figure S20. ^{1}H NMR DOSY spectrum (850 MHz, 298K) of [1+1] capsule **6** in CD_2Cl_2.

Monitoring the Formation of CBC 5 with ^{1}H NMR Spectroscopy

To a solution in CD_2Cl_2 (0.39 mL) containing basket **2** (0.298 µmol, 0.51 mM final conc.) and *tris*-amine **4** (0.327 µmol, 0.56 mM final conc., 1.1 eq), a solution in CD_2Cl_2 (0.2 mL) of trifluoroacetic acid (0.584 µmol, 0.990 mM final conc., 2.0eq) was added in an NMR tube (0.59 mL total vol.). The sample was shaken vigorously and immediately loaded into the NMR (600 MHz, 300K). The reaction was monitored overtime with ^{1}H NMR spectroscopy (Figure S18).

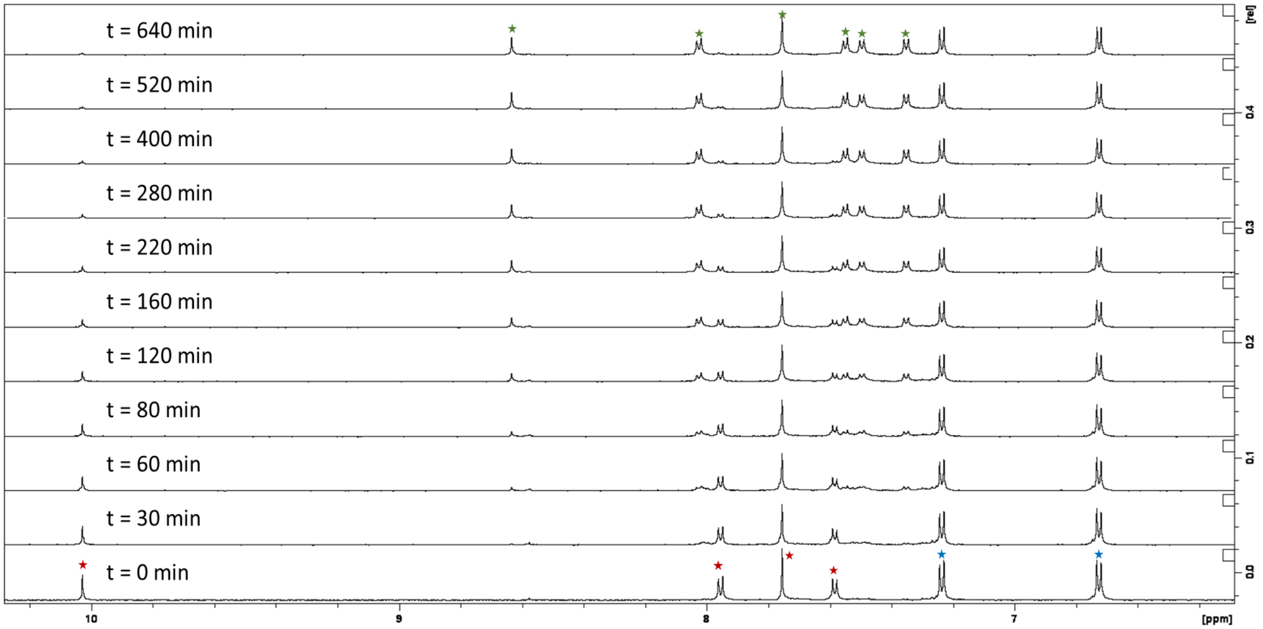

Figure S21. The acid-catalyzed reaction between basket **2** (red stars) and *tris*-amine **4** (blue stars) to give CBC **5** (green stars) monitored over time with ^{1}H NMR spectroscopy (600 MHz, 300K, CD_2Cl_2).

Reversible Formation/Degradation of CBC 5

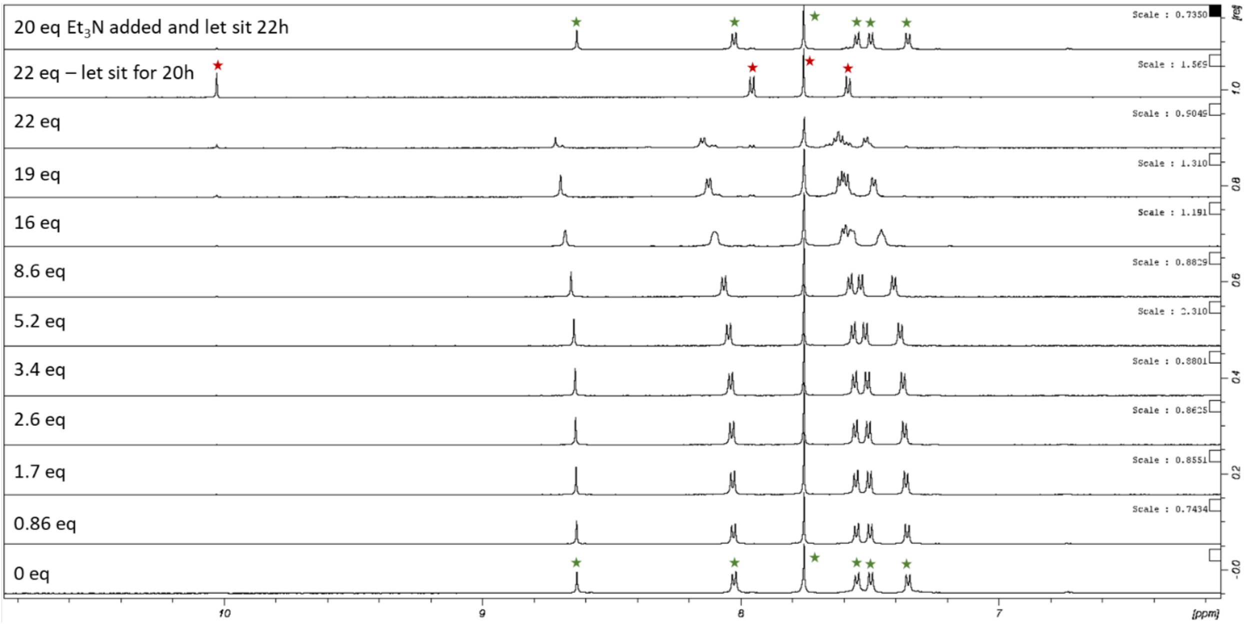

Figure S22. Partial ^{1}H NMR spectra (600 MHz, 300K, CD_2Cl_2) of CBC **5** (0.25 mM, green stars) taken immediately after the addition of increasing amounts of TFA. After the addition of 22 equivalents of TFA with respect to **5**, the sample was let sit for 20h which had resulted in the complete disintegration of the cage. Note that basket **2** (red stars) was present but *tris*-amine **4** was not visible after the cage disassembled. The protonation of *tris*-amine 4 with an excess of TFA caused it to fall out of solution which was corroborated by the observation of a precipitate. The addition of 20 equivalents of triethylamine to the solution and letting it sit for 22h allowed the reformation of CBC **5** (green stars).

Supramolecular Titrations with CBr_4

Due to their poor solubilities, solutions of both basket **2** and CBC **5** were prepared by forming a saturated solution in CD_2Cl_2 and filtering any undissolved solids. Concentrations were then determined by ^1H NMR spectroscopy using an internal standard (nitromethane or 1,3,5-trimethoxy benzene). Incremental amounts of CBr_4 were added and the chemical shifts of the **2** and **5** were monitored at 300K (Figures S20-21).

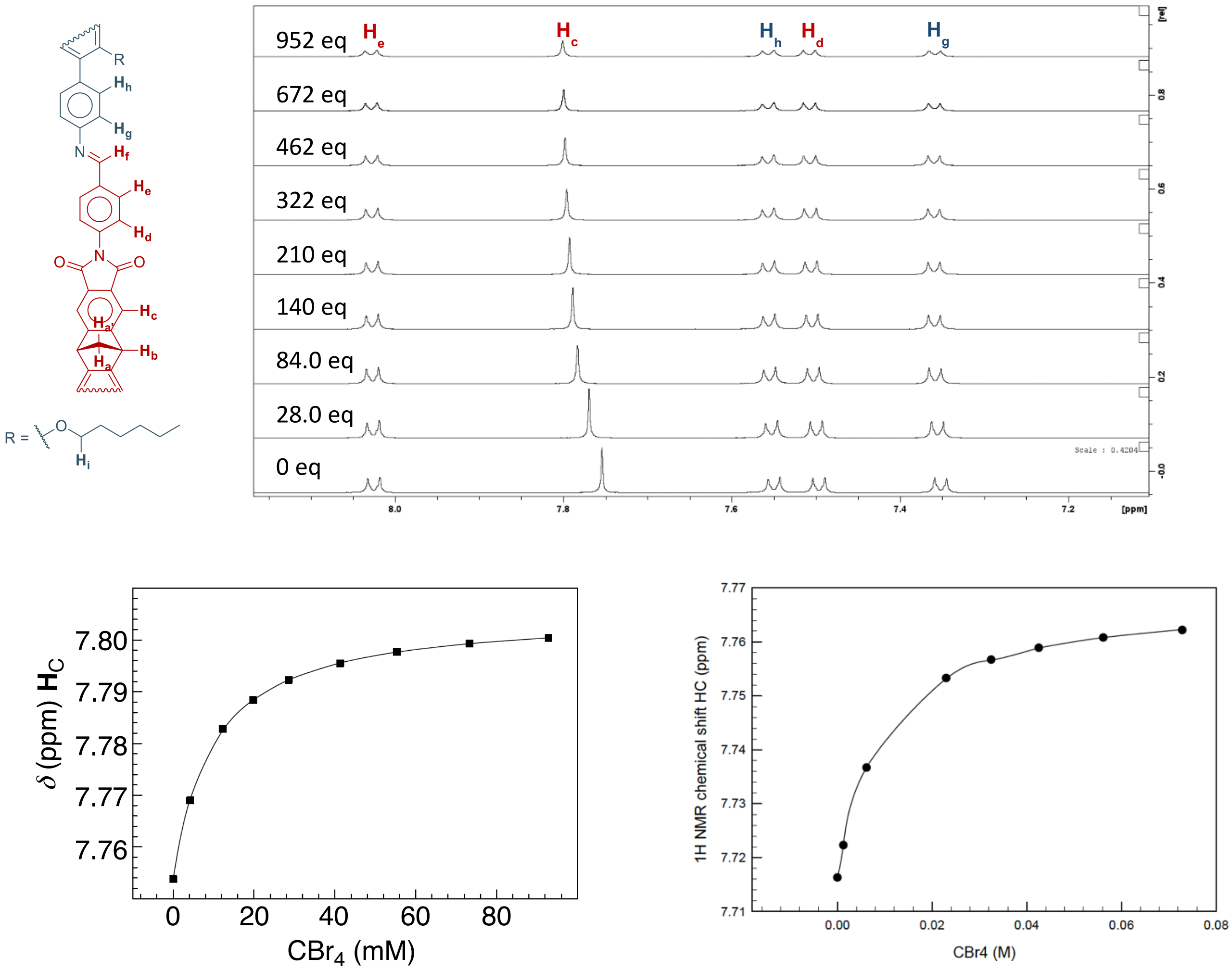

Figure S23. Top: Partial ^1H NMR spectra of 0.15 mM CBC **5** (600 MHz, 300K) in CD_2Cl_2 obtained after incremental addition of a standard solution of CBr_4. Bottom: A nonlinear least-square analysis of the binding isotherms (two independent supramolecular titrations, SigmaPlot) fit well to the formation of a binary complex with K_a = 107 (left) and K_a = 110 (right) so that K_a = 108 (mean) ± 2 M^{-1} (standard deviation).

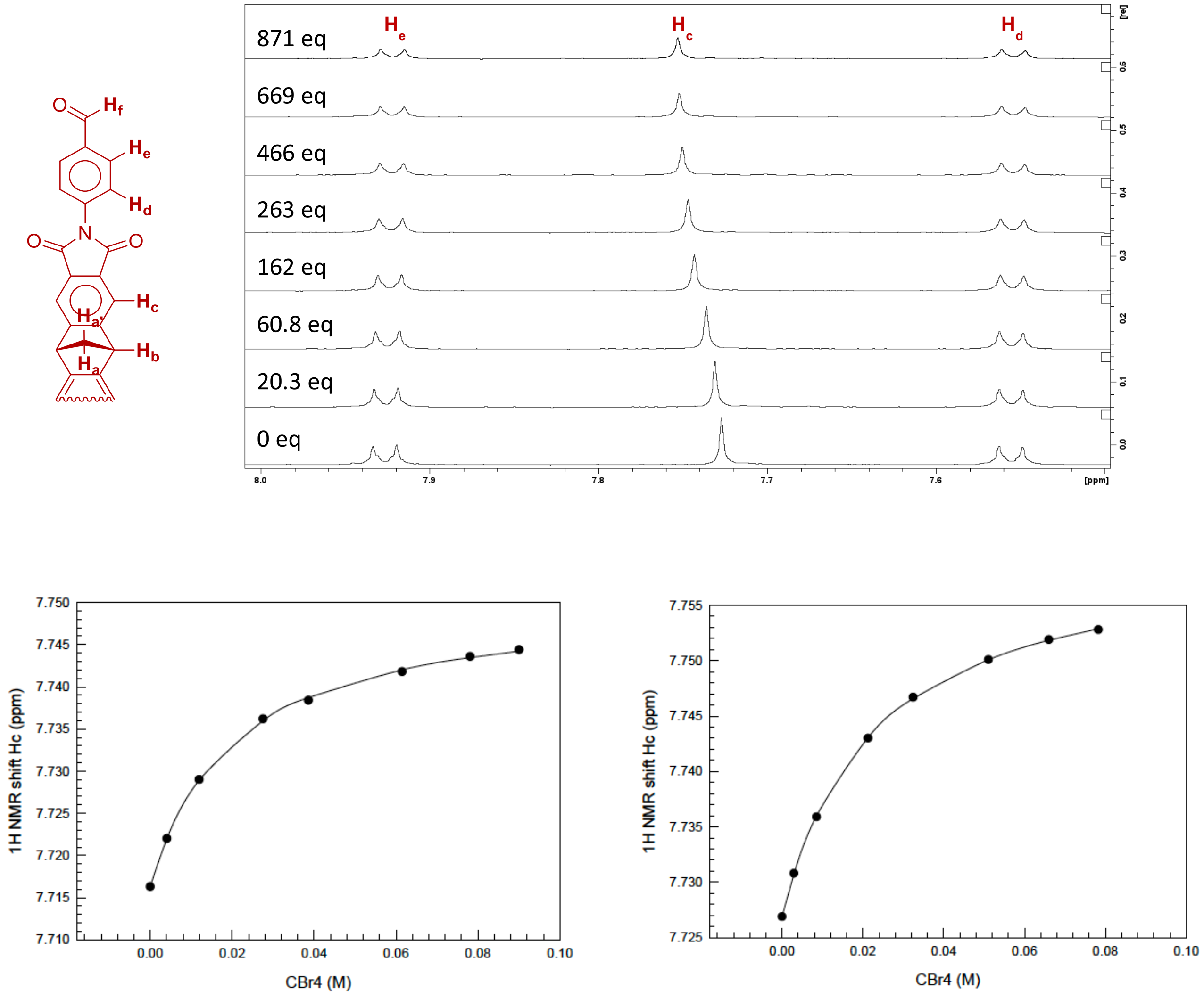

Figure S24. (Top) Partial ^{1}H NMR spectra of 0.13 mM basket **2** (600 MHz, 300K) in CD_2Cl_2 obtained after incremental addition of a standard solution of CBr_4. (Bottom) A nonlinear least-square analysis of the binding isotherms (two independent supramolecular titrations, SigmaPlot) fit well to the formation of a binary complex with K_a = 44 (left) and K_a = 49 (right) so that K_a = 46 (mean) ± 4 M^{-1} (standard deviation).

Switching Between [1+1] cage 6 and CBC 5

To a solution in CD_2Cl_2 (0.72 mL) containing CBC **5** (0.091 mM), *tris*-amine **7** (0.89 mM), and trimethoxy benzene (0.54 mM, internal standard), a solution of TFA in CD_2Cl_2 (138 mM) was added (7.6 µL, 15eq with respect to **5**, final conc. TFA 1.4 mM). ^{1}H NMR of resulting solution was recorded after letting it equilibrate at r.t. for 29h, without stirring. The [4+4] cage **5** had converted to [1+1] capsule **6** as the primary species**,** leaving free *tris*-amine **4** and excess [**7**-H_n]$^{n+}$ in the mixture. An additional portion of TFA solution (220 mM) was added (9.6 µL, 32eq with respect to **5**, total conc. TFA 4.3 mM). ^{1}H NMR of the resulting solution was recorded after letting it equilibrate at r.t. for 15h, without stirring. [1+1] capsule **6** had largely disassembled forming CBC **5** (Figures S22-S23).

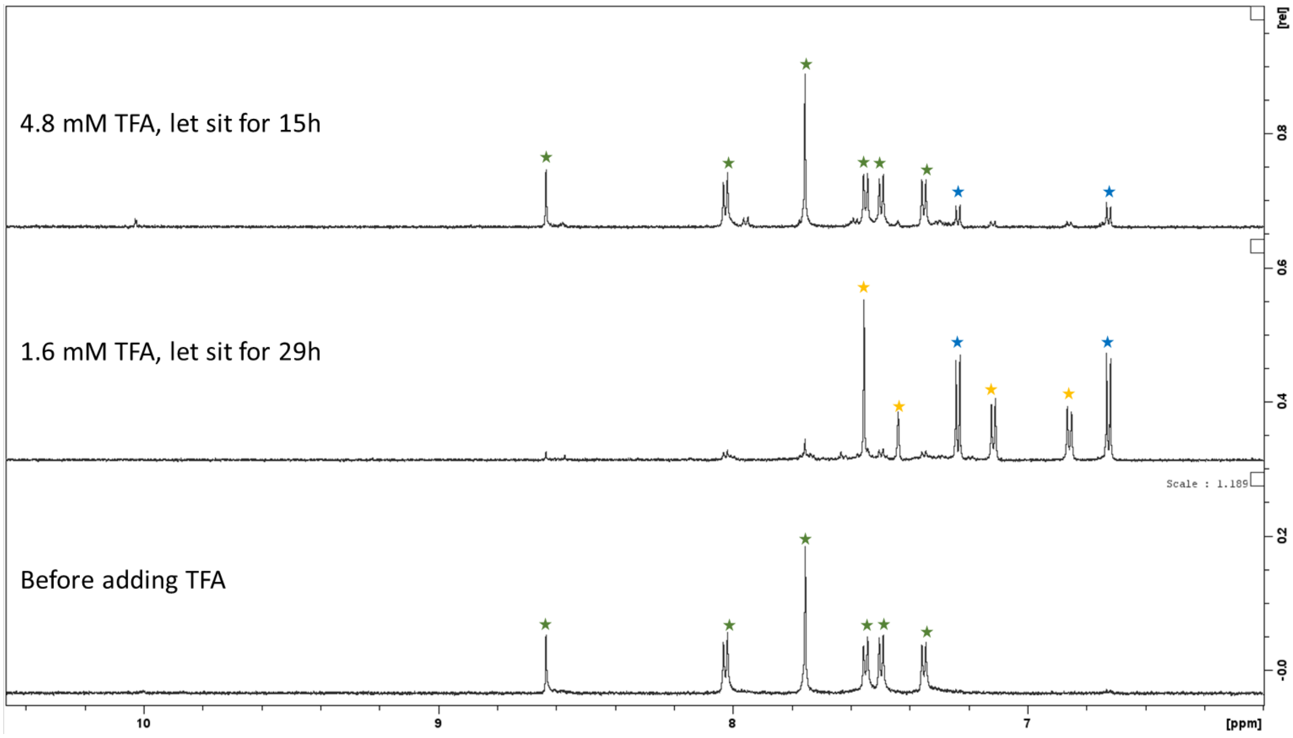

Figure S25. Partial ^{1}H NMR spectra (600 MHz, 300K) of CBC **5** and *tris*-amine **7** before and after the addition of different amounts of TFA (see above for details of the protocol). ^{1}H NMR Resonances from [1+1] capsule **6** (yellow stars), CBC **5** (green stars), and *tris*-amine **4** (blue stars) are labeled.

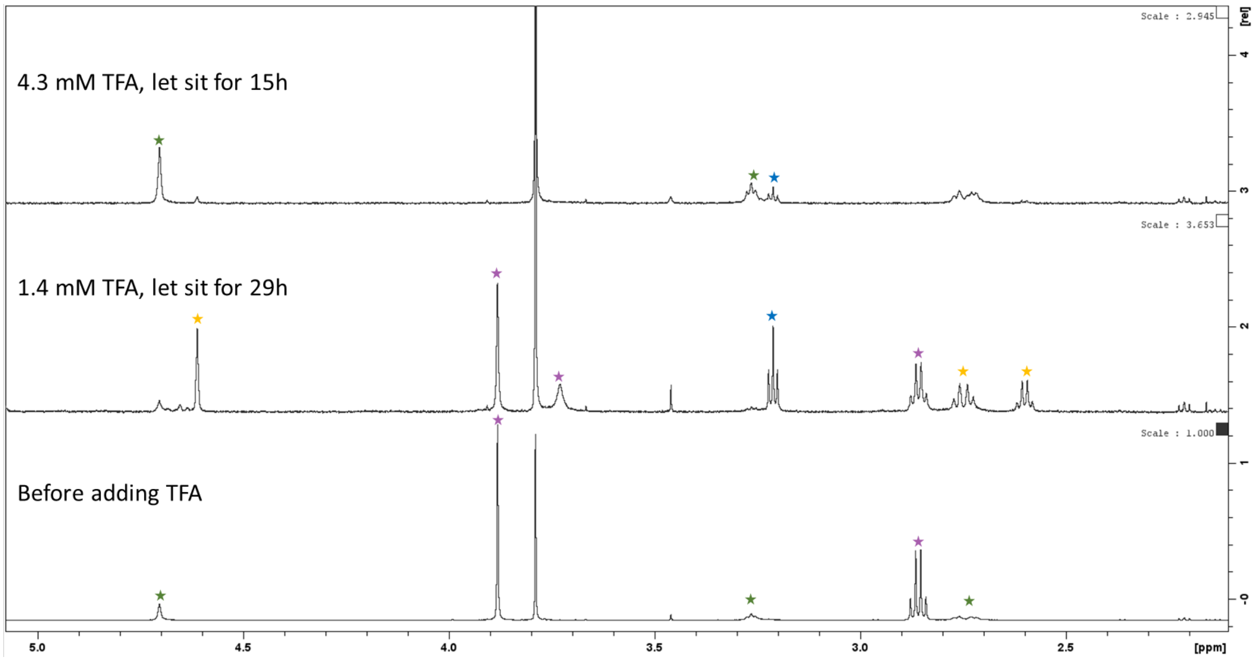

Figure S26. Partial ^{1}H NMR spectra (600 MHz, 298K) of CBC **5**, *tris*-amine **7** before and after the addition of different amounts of TFA (see above for details of the protocol). ^{1}H NMR Resonances from [1+1] capsule **6** (yellow stars), CBC **5** (green stars), *tris*-amine **4** (blue stars), and *tris*-amine **7** (purple stars) are labeled.

Fuel-Driven Reaction Cycles

A solution (0.63 mL) containing CBC **5** (0.11 mM), *tris*-amine **7** (1.1 mM), 1,3,5-trimethoxy benzene (0.29 mM, internal standard) and trifluoroacetic acid (1.70 mM) was prepared in CD_2Cl_2. This solution was allowed to equilibrate for 18h at a room temperature giving [1+1] capsule **6** as the primary species. The starting concentrations of [1+1] cage **6** (0.44 mM), free *tris*-amine **4** (0.44 mM), and free *tris*-amine **7** (0.66 mM) were calculated based on the assumption of full conversion of **5** to **6**. Tribromoacetic acid (TBA; 2.7 mM final concentration) was added to the solution and the sample was immediately loaded into the NMR spectrometer (850 MHz, 298K). The reaction progress was monitored over time with ^{1}H NMR spectroscopy (Figures S27-S31).

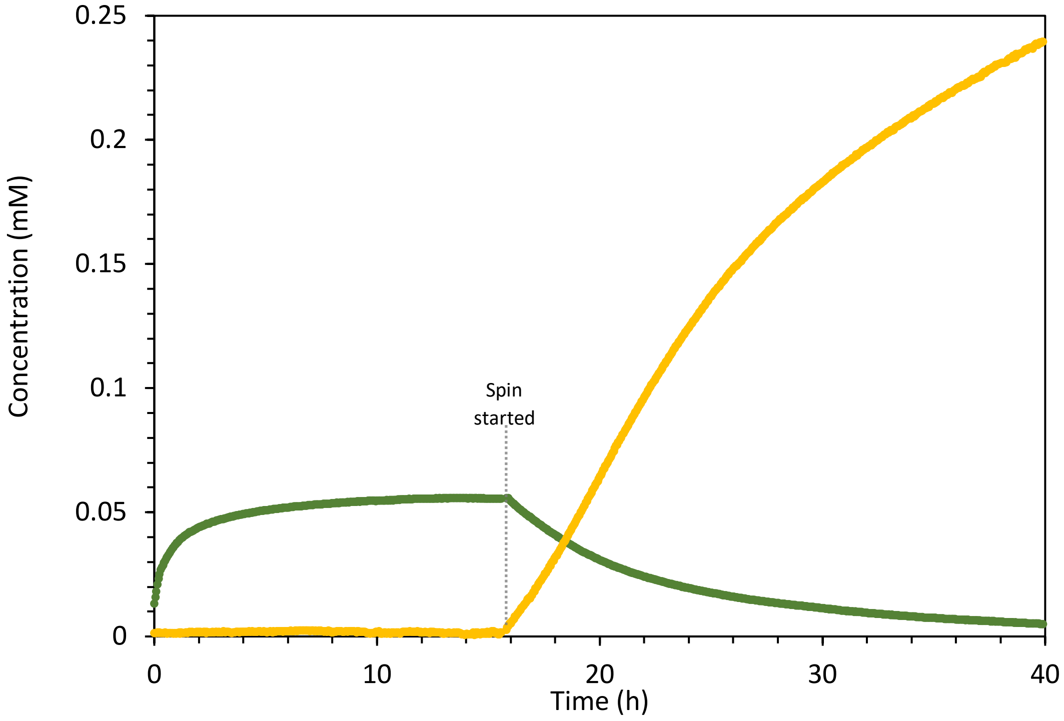

Figure S27. A plot showing the concentration of CBC **5** (green) and [1+1] capsule **6** (yellow) after the addition of TBA monitored overtime with ^{1}H NMR spectroscopy (850 MHz, 298K). Concentrations determined with 1,3,5-trimethoxy benzene as internal standard (see above for details of the protocol).

When spinning of the sample within NMR spectrometer was turned off, the reaction would not proceed beyond the formation of CBC **5** up to 15h (Figure S24). We attribute this to a reduced accessible surface of precipitate [**7**-H_n]$^{n+}$ slowing decarboxylation of TBA. As soon as the sample spinning starts, the conversion of CBC **5** to [1+1] capsule **6** begins to accelerate (Figure S24).

Repeated dissipation cycle: To the solution above, an additional portion of TBA was added (2.5 mM final concentration) and the reaction was monitored with ^{1}H NMR spectroscopy (Figures S25-S28). In this case, we assumed the starting concentrations of capsule **6** (0.44 mM), *tris*-amine **4** (0.44 mM), *tris*-amine 7 (0.66 mM), and trifluoroacetic acid (1.7 mM) based on the starting point of the previous reaction. The concentration of CBC **5** reached a maximum of 0.046 mM at about 5.6h and then decreased over time (Figure S30). [1+1] Capsule **6** started to form at about 5.6h as well, with its concentration plateauing at 0.22 mM after 24h. The decarboxylation of TBA was monitored with the formation of $CHBr_3$. Interestingly, the kinetic profile corresponding to decarboxylation of TBA is sigmoidal in shape (Figure S28). The sudden acceleration in the formation of $CHBr_3$ coincides with the appearance of [1+1] cage **6** (Figure S28).

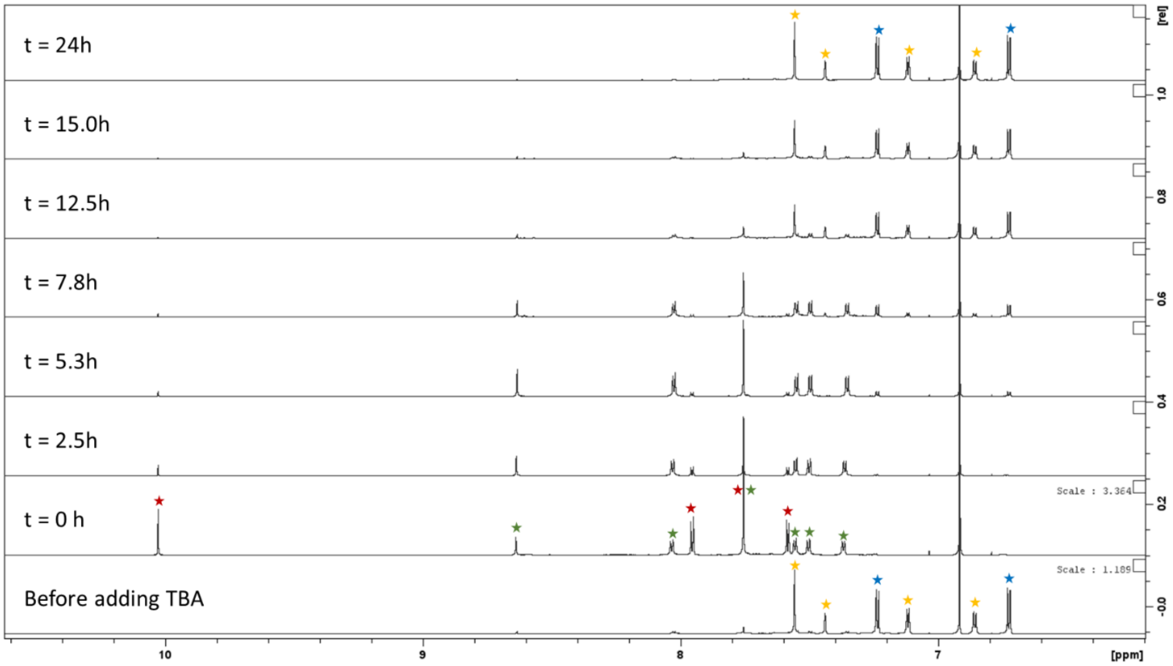

Figure S28. Selected and partial ^{1}H NMR (850 MHz, 298K) spectra of [1+1] capsule **6**, *tris*-amines **4** and **7** along with TFA before and after the addition of TBA. ^{1}H NMR Resonances from [1+1] capsule **6** (yellow stars), CBC **5** (green stars), *tris*-amine **4** (blue stars), and basket **2** (red stars) are labeled.

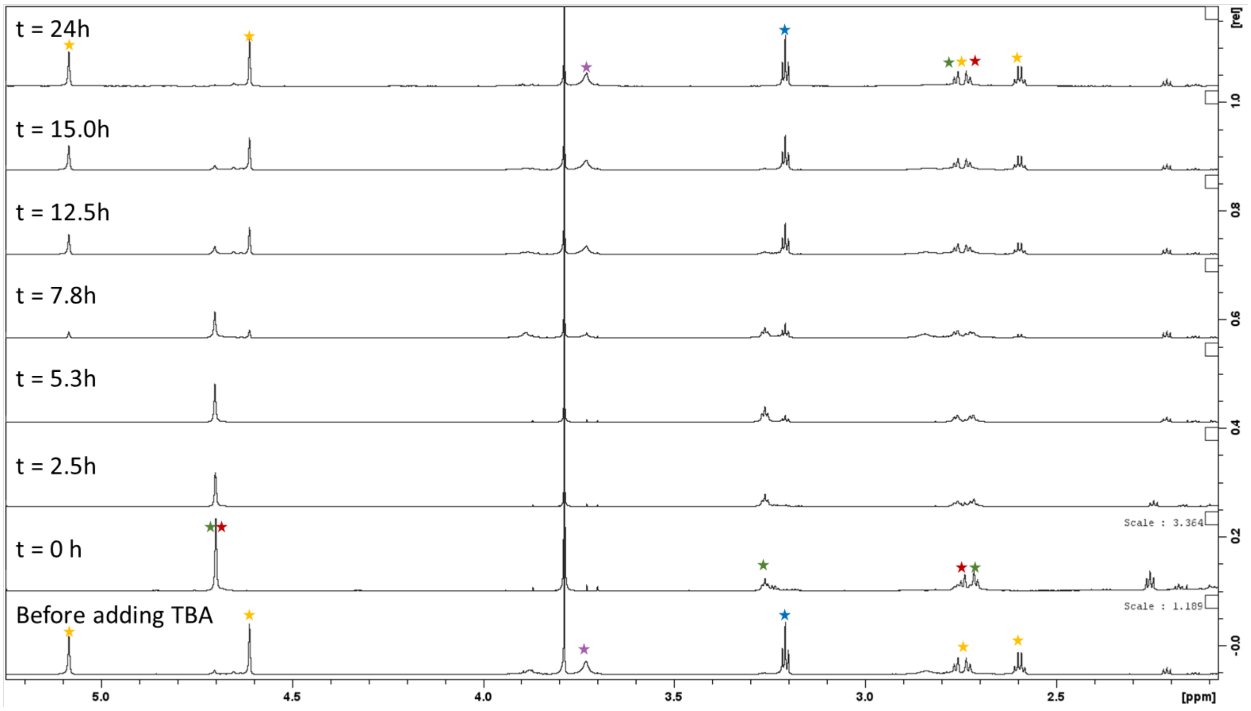

Figure S29. Selected and partial ^{1}H NMR (850 MHz, 298K) spectra of [1+1] cage **6**, *tris*-amine **7** and TFA were after the addition of TBA. ^{1}H NMR Resonances from [1+1] capsule **6** (yellow stars), CBC **5** (green stars), *tris*-amine **4** (blue stars), *tris*-amine **7** (purple stars) and basket **2** (red stars) are labeled.

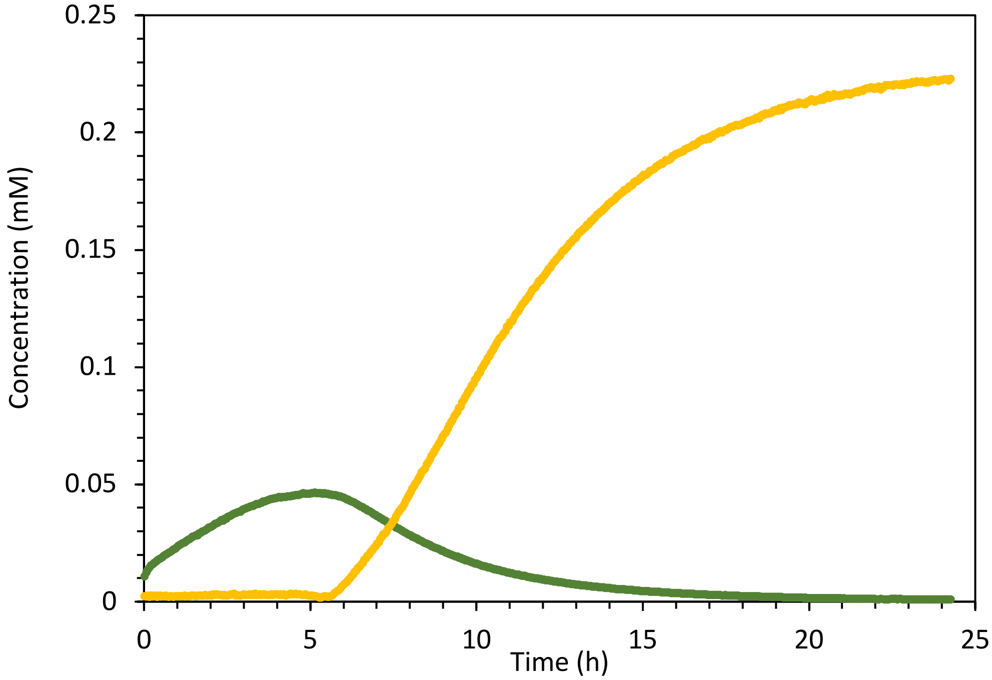

Figure S30. A change in the concentration of CBC **5** (green) and [1+1] capsule **6** (yellow) was monitored with time after the addition of TBA to the reaction mixture and using 1,3,5-trimethoxy benzene as internal standard (^{1}H NMR spectroscopic measurements in Figures S25-26).

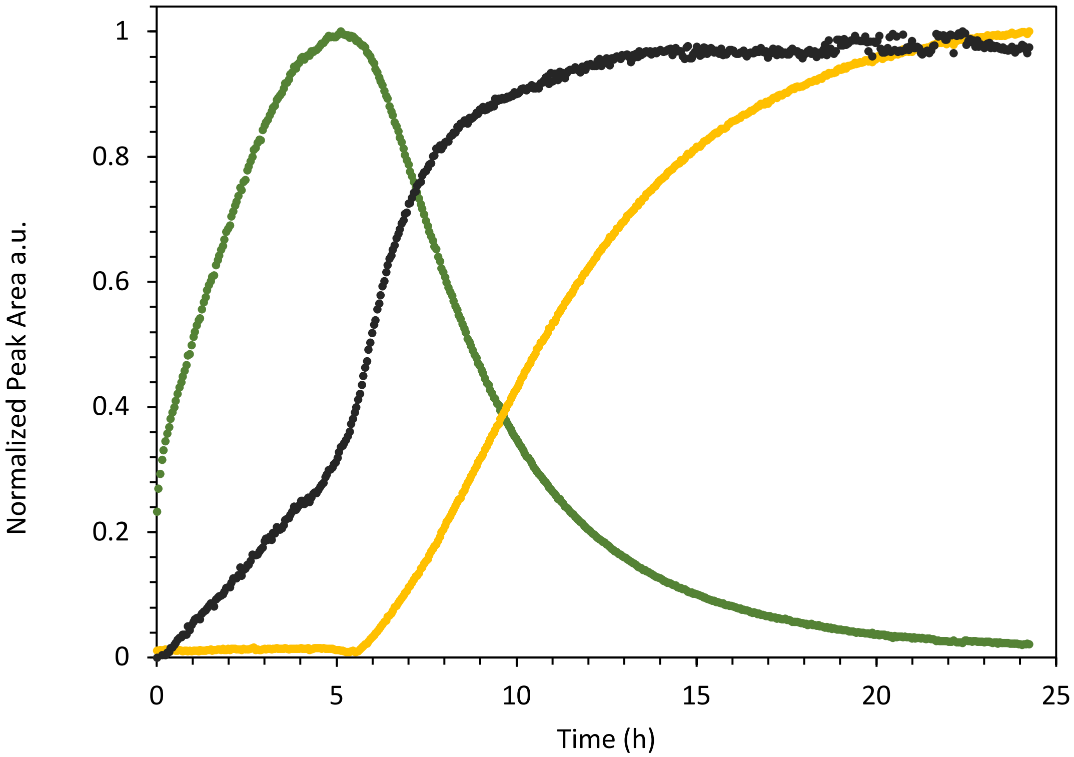

Figure S31. A plot showing ^{1}H NMR peak areas (Figures S25-26) normalized to the maximum of each species, after the dissipation cycle was started, for: [4+4] cage **5** (green), [1+1] capsule **6** (yellow) and $CHBr_3$ (black).

Dissipation Cycles with Different Amounts of TBA

A solution containing [1+1] cage **6** (0.41 mM), *tris*-amine **4** (0.41 mM), *tris*-amine **7** (0.61 mM), 1,3,5-trimethoxy benzene (0.51 mM, internal standard) and trifluoroacetic acid (1.6 mM) was prepared in 2.1 mL of CD_2Cl_2. An aliquot (0.60 mL) of this solution was added to an NMR tube for each dissipation cycle. An appropriate amount of 150 mM solution of TBA (final concentrations of 2.0, 2.8, and 3.6 mM) was added to the tube and mixed vigorously. The sample was immediately loaded into the NMR spectrophotometer (850 MHz, 298K) and the reaction was monitored over time as shown in Figures S29-36.

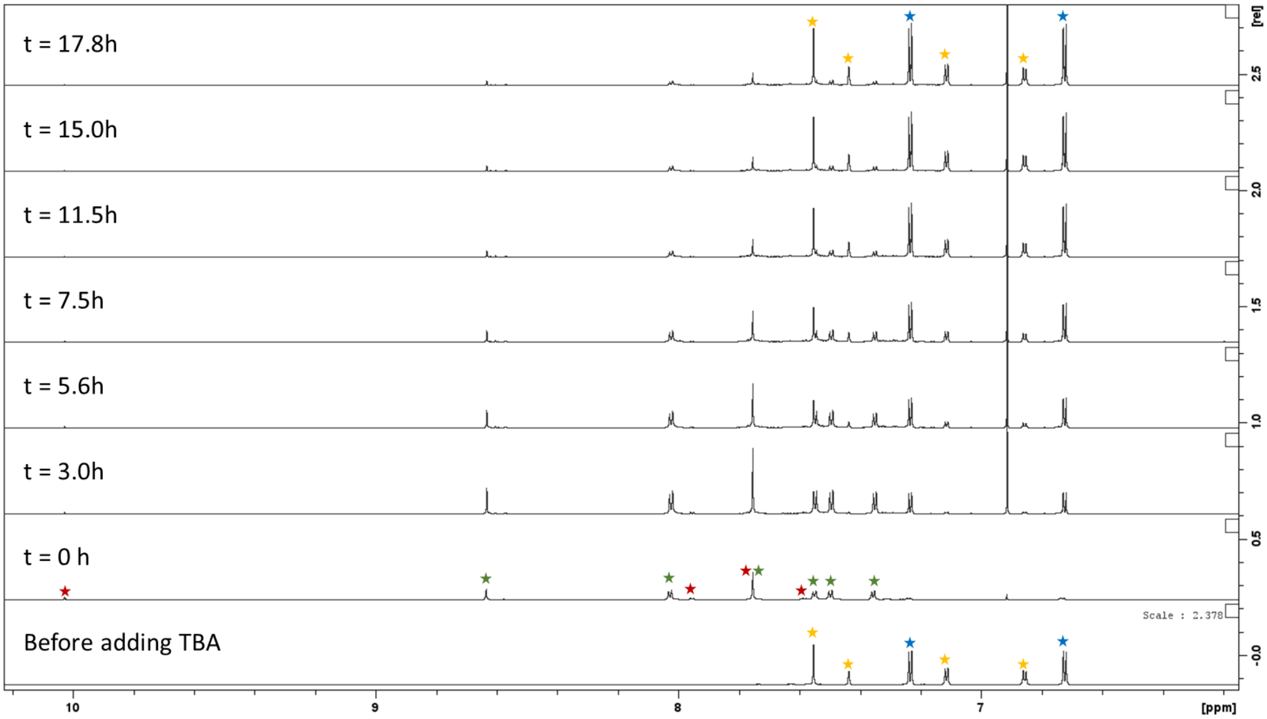

Figure S32. Selected and partial ^1H NMR (850 MHz, 298K) spectra of [1+1] cage **6**, *tris*-amine **4**, *tris*-amine **7** and TFA obtained after the addition of 2.0 mM TBA. ^1H NMR Resonances from [1+1] capsule **6** (yellow stars), CBC **5** (green stars), *tris*-amine **4** (blue stars), and basket **2** (red stars) are labeled.

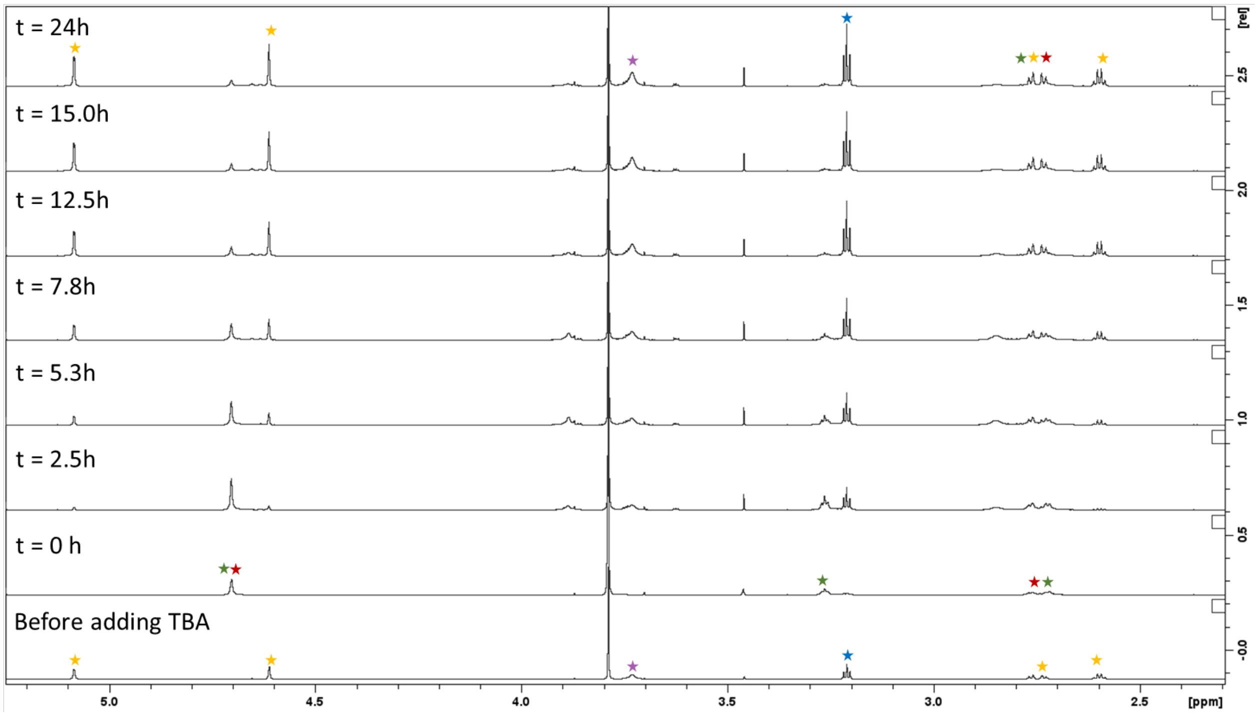

Figure S33. Selected and partial ^{1}H NMR (850 MHz, 298K) spectra of [1+1] cage **6**, *tris*-amine **4**, *tris*-amine **7** and TFA obtained after the addition of 2.0 mM TBA. ^{1}H NMR Resonances from [1+1] capsule **6** (yellow stars), CBC **5** (green stars), *tris*-amine **4** (blue stars), *tris*-amine **7** (purple stars) and basket **2** (red stars) are labeled.

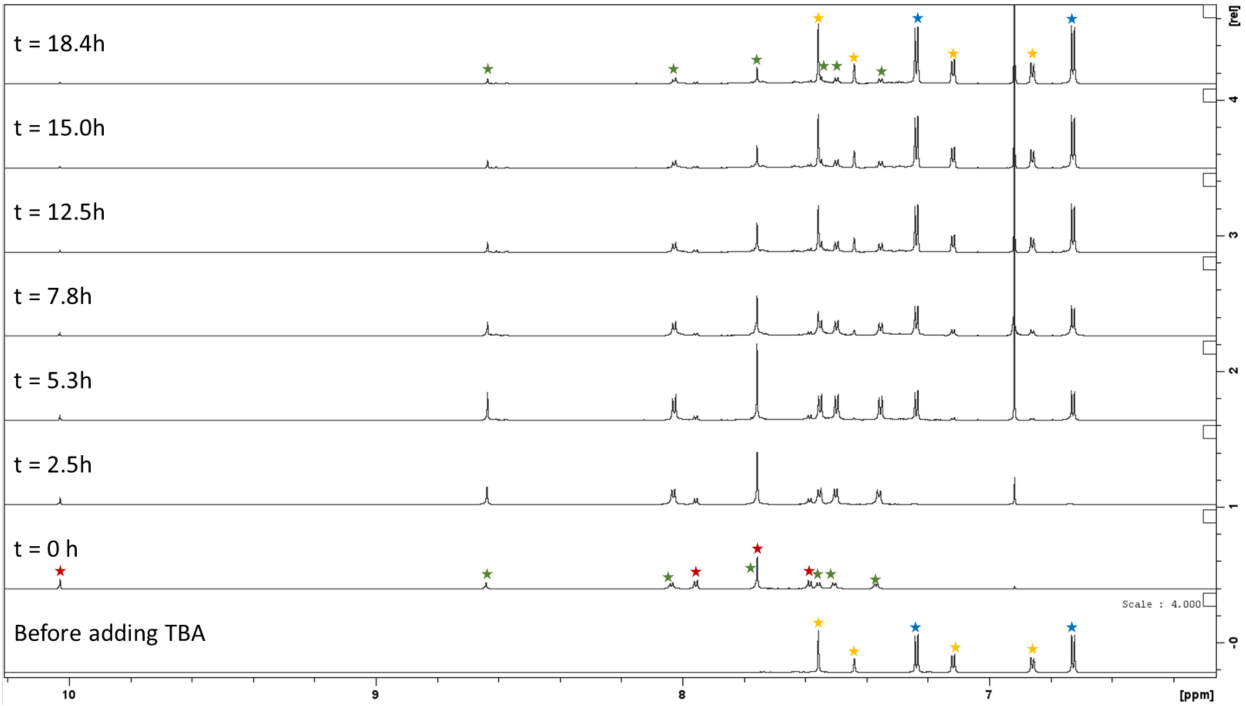

Figure S34. Selected and partial ^{1}H NMR (850 MHz, 298K) spectra of [1+1] cage **6**, *tris*-amine **4**, *tris*-amine **7** and TFA obtained after the addition of 2.8 mM TBA. ^{1}H NMR Resonances from [1+1] capsule **6** (yellow stars), CBC **5** (green stars), *tris*-amine **4** (blue stars), and basket **2** (red stars) are labeled.

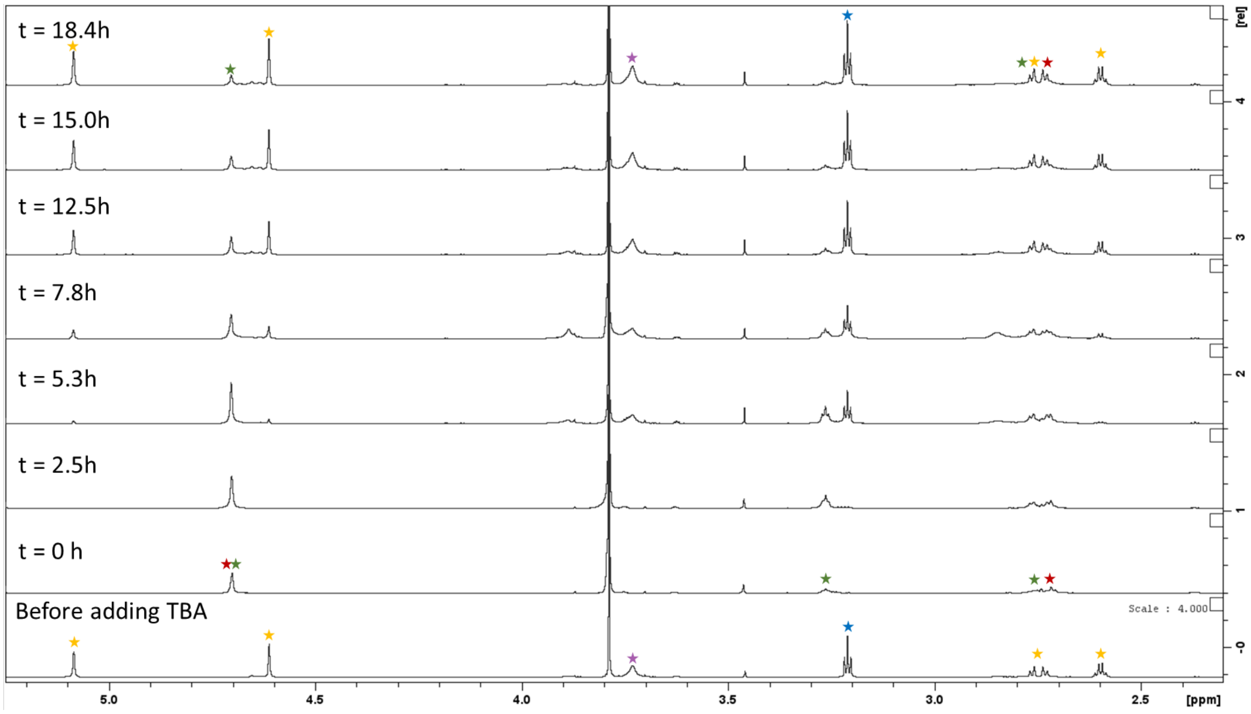

Figure S35. Selected and partial ^{1}H NMR (850 MHz, 298K) spectra of [1+1] cage **6**, *tris*-amine **4**, *tris*-amine **7** and TFA obtained after the addition of 2.8 mM TBA. ^{1}H NMR Resonances from [1+1] capsule **6** (yellow stars), CBC **5** (green stars), *tris*-amine **4** (blue stars), *tris*-amine **7** (purple stars) and basket **2** (red stars) are labeled.

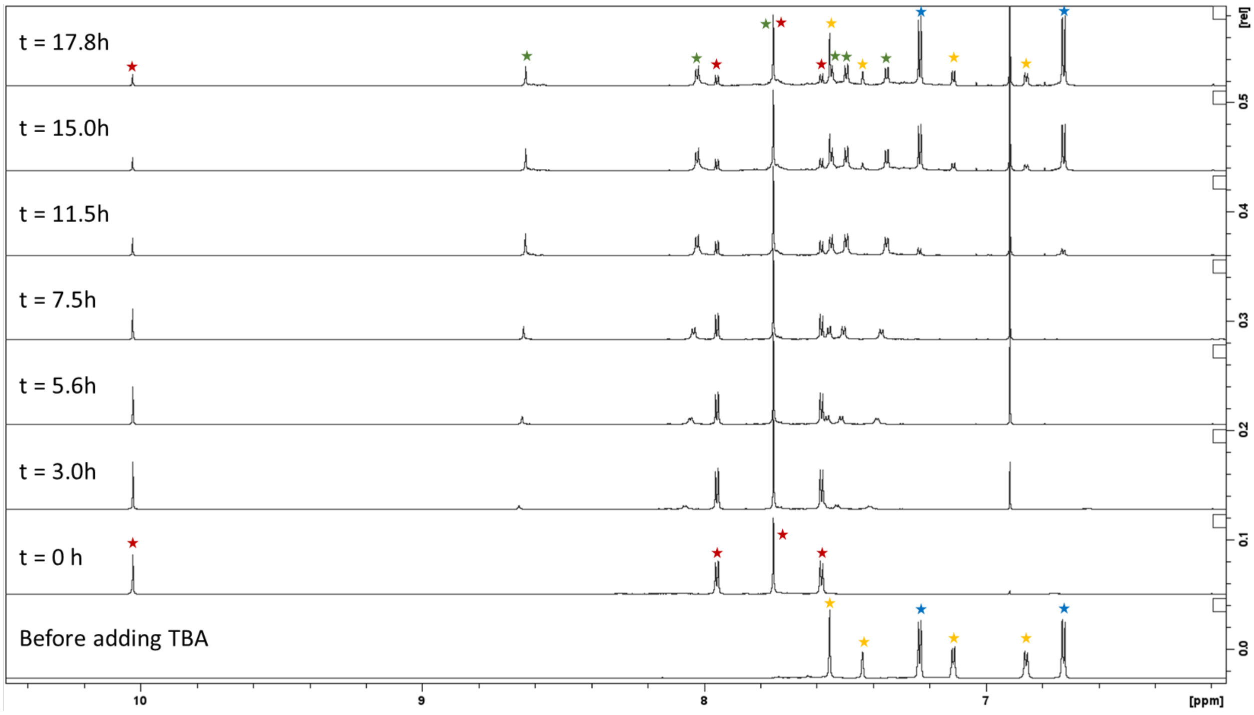

Figure S36. Selected and partial ^{1}H NMR (850 MHz, 298K) spectra of [1+1] cage **6**, *tris*-amine **4**, *tris*-amine **7** and TFA obtained after the addition of 3.6 mM TBA. ^{1}H NMR Resonances from [1+1] capsule **6** (yellow stars), CBC **5** (green stars), *tris*-amine **4** (blue stars), and basket **2** (red stars) are labeled.

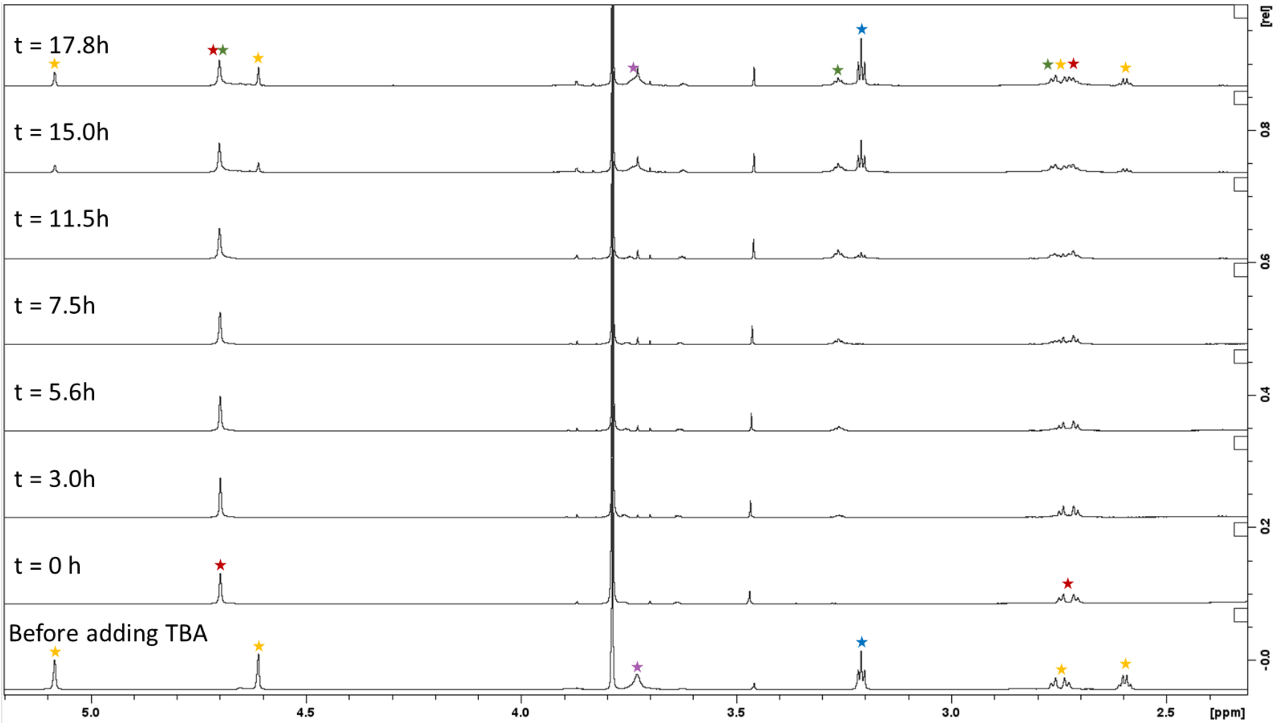

Figure S37. Selected and partial ^{1}H NMR (850 MHz, 298K) spectra of [1+1] cage **6**, *tris*-amine **4**, *tris*-amine **7** and TFA obtained after the addition of 3.6 mM TBA. ^{1}H NMR Resonances from [1+1] capsule **6** (yellow stars), [4+4] cage **5** (green stars), *tris*-amine **4** (blue stars), *tris*-amine **7** (purple stars) and basket **2** (red stars) are labeled.

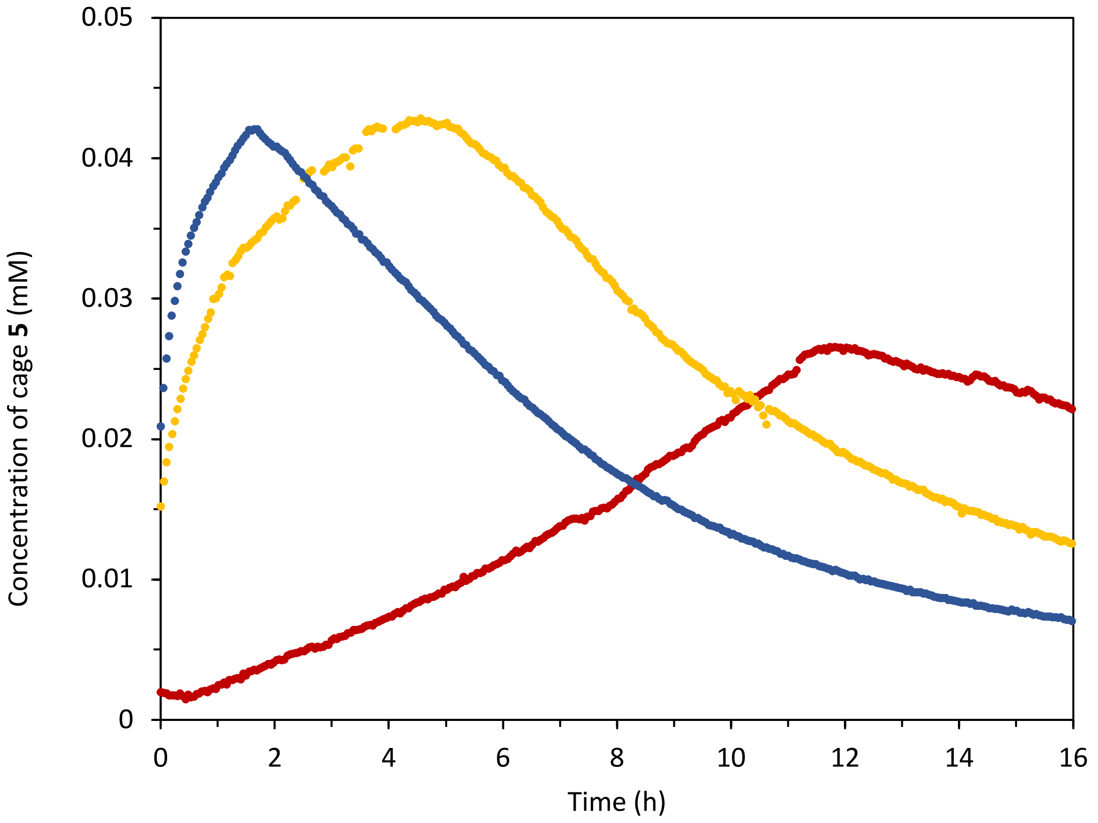

Figure S38. Plot showing the concentration of CBC **5** over time after the addition of 5 (blue), 7 (yellow) and 9 (red) equivalents of TBA. Concentrations were determined using ^{1}H NMR spectroscopy (Figures S29-S34) with trimethoxy benzene as internal standard.

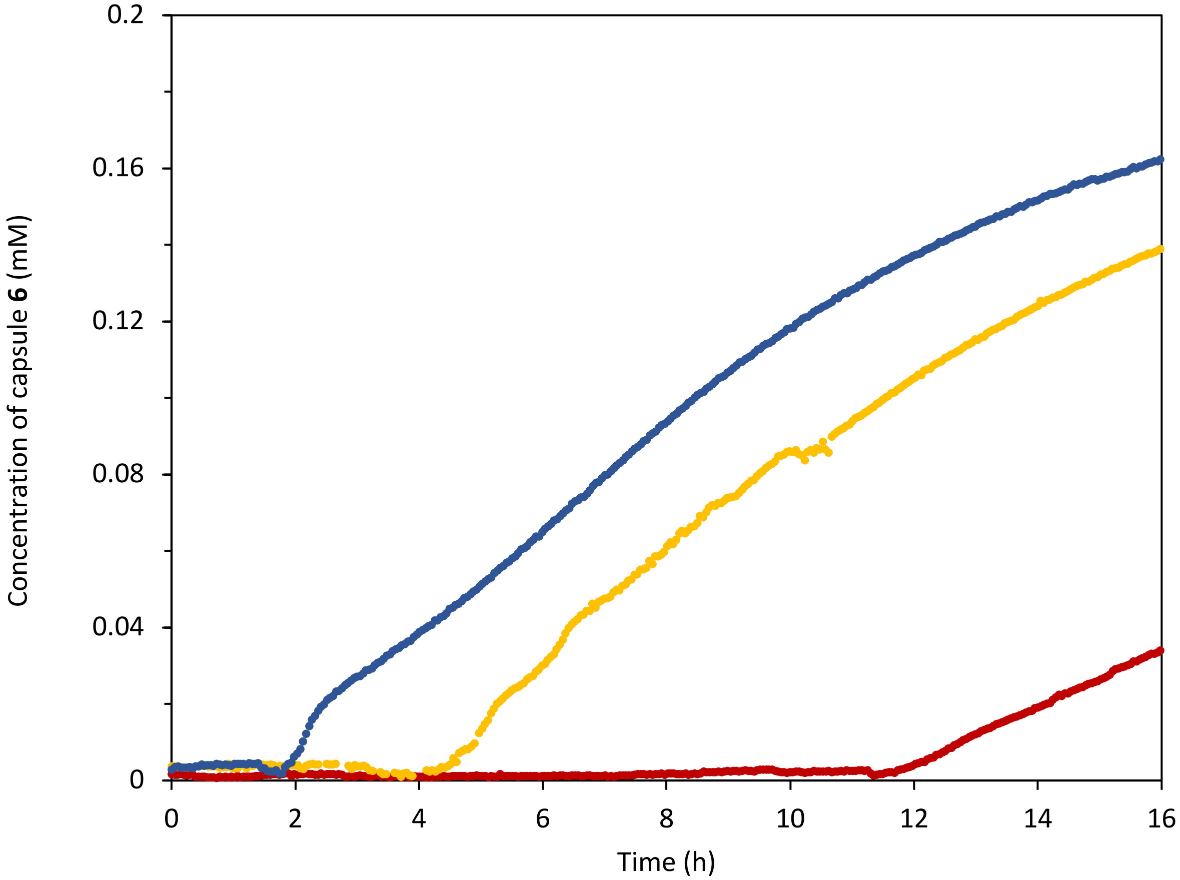

Figure S39. A plot showing the concentration of [1+1] capsule **6** over time after the addition of 5 (blue), 7 (yellow) and 9 (red) equivalents of TBA. Concentrations were determined using ^{1}H NMR spectroscopy (Figures S29-S34) with trimethoxy benzene as internal standard.

Successive Repetitions of Dissipative Cycles

A solution containing [1+1] cage **6** (0.41 mM), *tris*-amine **4** (0.41 mM), *tris*-amine **7** (0.61 mM), 1,3,5-trimethoxy benzene (0.75 mM, internal standard) and trifluoroacetic acid (1.6 mM) was prepared in 0.65 mL of CD_2Cl_2. TBA solution in CD_2Cl_2 (136mM) was added (12.2 µL, final conc. 2.6 mM) to the solution which was then shaken thoroughly. ^{1}H NMR spectrum was recorded (800 MHz, 298K) at 10 min, 5 h, and 24 h after the addition of TBA. Only 60-80% of TBA was consumed 24h following the initial addition (determined by the amount of $CHBr_3$ formed). Subsequent cycles were performed by adding TBA to adjust to 2.6 mM of fuel and recording ^{1}H NMR (800 MHz, 298K) at 10 min, 5 h, and 24 h after addition.

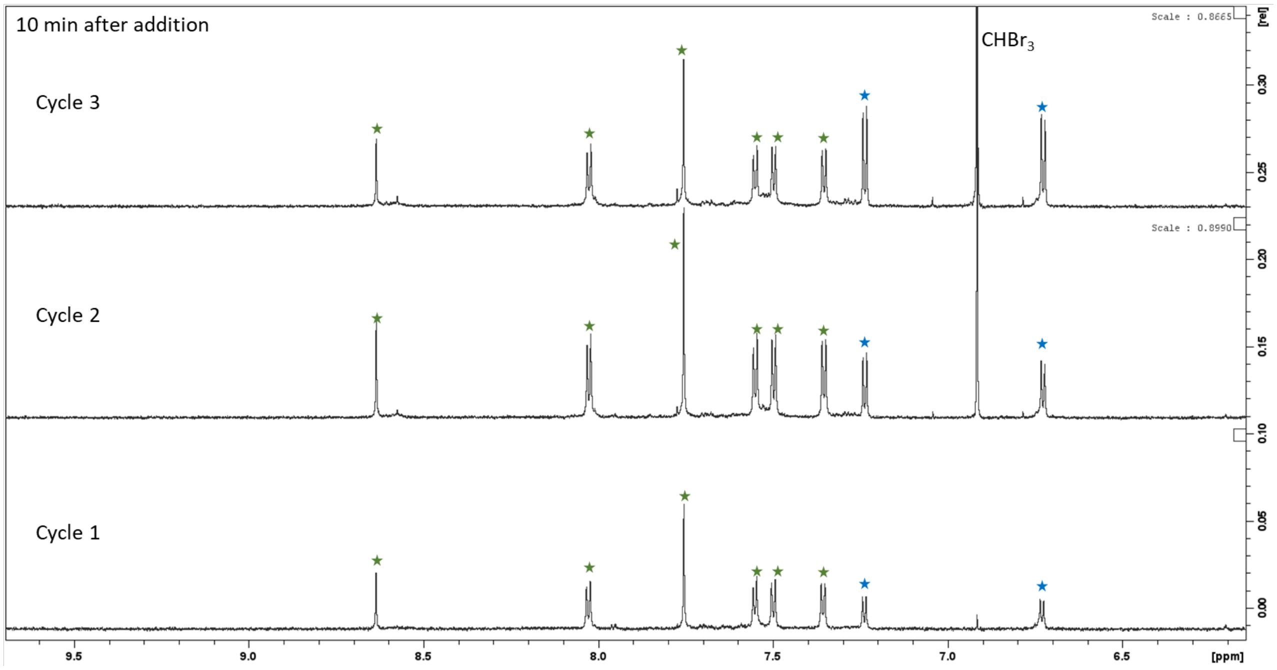

Figure S40. Partial ^{1}H NMR spectra (800 MHz, 298K) of a solution of [1+1] cage **6**, *tris*-amine **4**, *tris*-amine **7** and TFA obtained 10 mins after addition of 2.6 mM TBA in three successive cycles (see protocol above for details). ^{1}H NMR Resonances from [4+4] cage **5** (green stars), and *tris*-amine **4** (blue stars) are labeled.

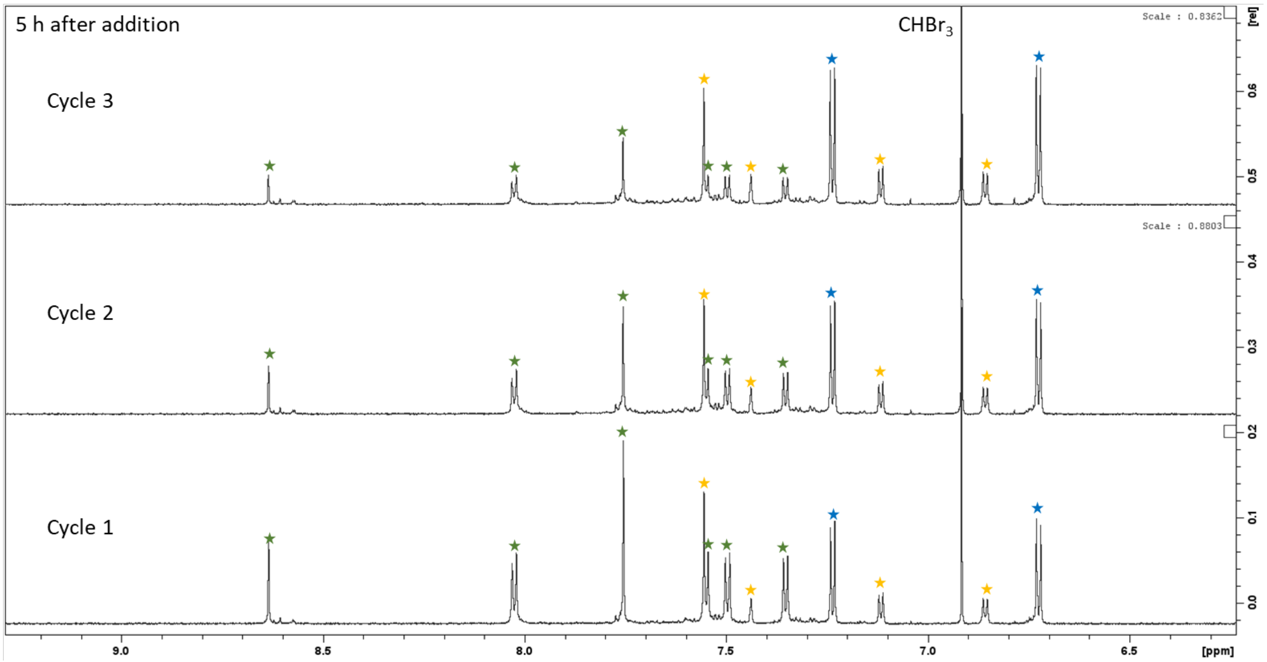

Figure S41. Partial ^{1}H NMR (800 MHz, 298K) spectra of a solution of [1+1] cage **6**, *tris*-amine **4,** *tris*-amine **7** and TFA obtained 5 h after the addition of 2.6 mM TBA in three successive cycles (see protocol above for details). ^{1}H NMR Resonances from [4+4] cage **5** (green stars), [1+1] cage **6** (yellow stars) and *tris*-amine **4** (blue stars) are labeled.

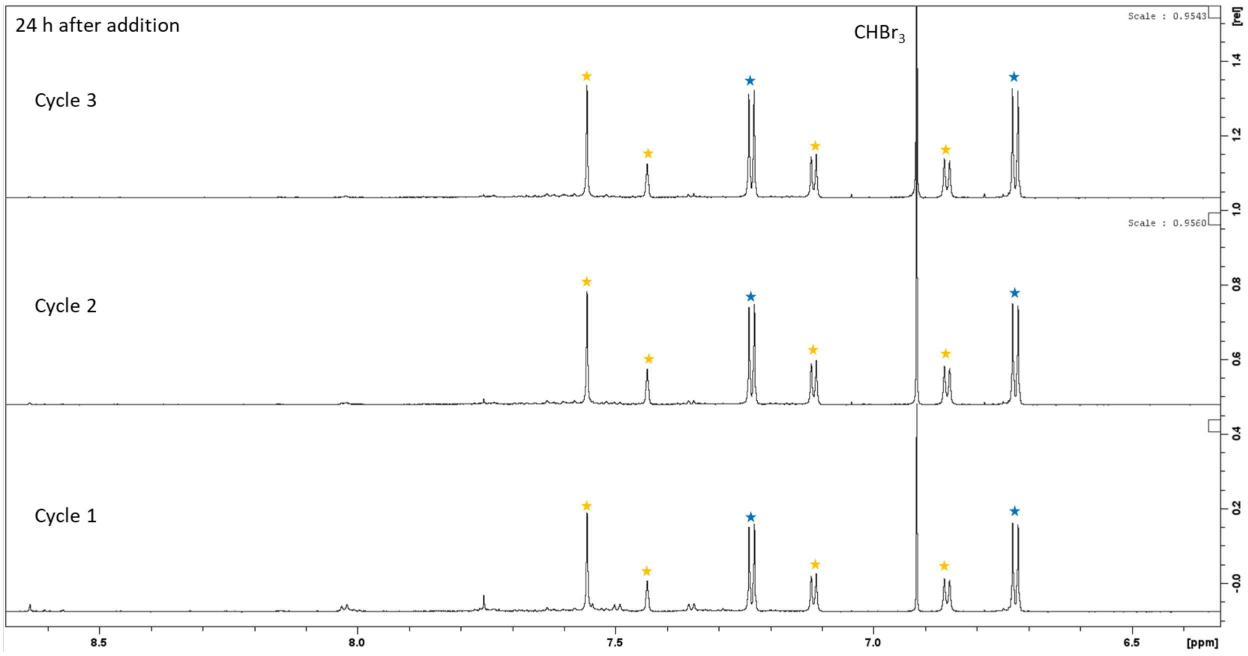

Figure S42. Partial ^{1}H NMR (800 MHz, 298K) spectra of a solution of [1+1] cage **6**, *tris*-amine **4**, *tris*-amine **7** and TFA obtained 5 h after the addition of 2.6 mM TBA in three successive cycles (see protocol above for details). ^{1}H NMR Resonances from [1+1] cage **6** (yellow stars), and *tris*-amine **4** (blue stars) are labeled.

X-Ray Crystallography

Check CIF reports: For compound **2** and [1+1] cage **6**, the B-level alerts are for the resolution of the data. Neither of these crystals diffracted past 0.90Å, so the data were cut at that point. For CBC **5**, all the A-level alerts and most of the B-level alerts are due to the resolution of the diffraction data. The crystal in most orientations didn't diffract much past 1.5Å, but we included data out to 1.3Å since the refinement didn't improve if we cut it at 1.5Å. The thermal parameters of the atoms were better with the data out to 1.3Å included. The only other B-level alert is for a long sp^2-sp^2 carbon-carbon bond generated by symmetry. We tried to restrain it to make it shorter, but due to the nature of the diffraction data and refinement restraints already in place, it made the structure refinement worse.

Experimental Summary for *tris*-Aldehyde Basket 2 (CCDC 2143946)

The single crystal X-ray diffraction studies were carried out on a Bruker Kappa Photon II CPAD diffractometer equipped with Cu K_α radiation (λ = 1.54178). A 0.322 x 0.034 x 0.026 mm colorless needle was mounted on a Cryoloop with Paratone 24EX oil. Data were collected in a nitrogen gas stream at 100(2) K using ϕ and ϖ scans. Crystal-to-detector distance was 40 mm using variable exposure time (30s-120s) depending on θ with a scan width of 1.0°. Data collection was 99.7% complete to 58.967° in θ (0.90Å). A total of 76602 reflections were collected covering the indices, -16<=h<=16, -24<=k<=24, -17<=l<=17. 7345 reflections were found to be symmetry independent, with a R_{int} of 0.0428. Indexing and unit cell refinement indicated a primitive, monoclinic lattice. The space group was found to be $P2_1/c$. The data were integrated using the Bruker SAINT software program and scaled using the SADABS software program. Solution by direct methods (SHELXT) produced a complete phasing model for refinement.

All nonhydrogen atoms were refined anisotropically by full-matrix least-squares (SHELXL-2014). All hydrogen atoms were placed using a riding model. Their positions were constrained relative to their parent atom using the appropriate HFIX command in SHELXL-2014. Due to unmodelable solvent disorder, OLEX2 SMTBX was used to mask out electron density from the lattice due to the disordered solvent. Solvent appeared to be a mix of water and methanol. One void was found with approximately 114 electrons. Crystallographic data are summarized in Table 1.

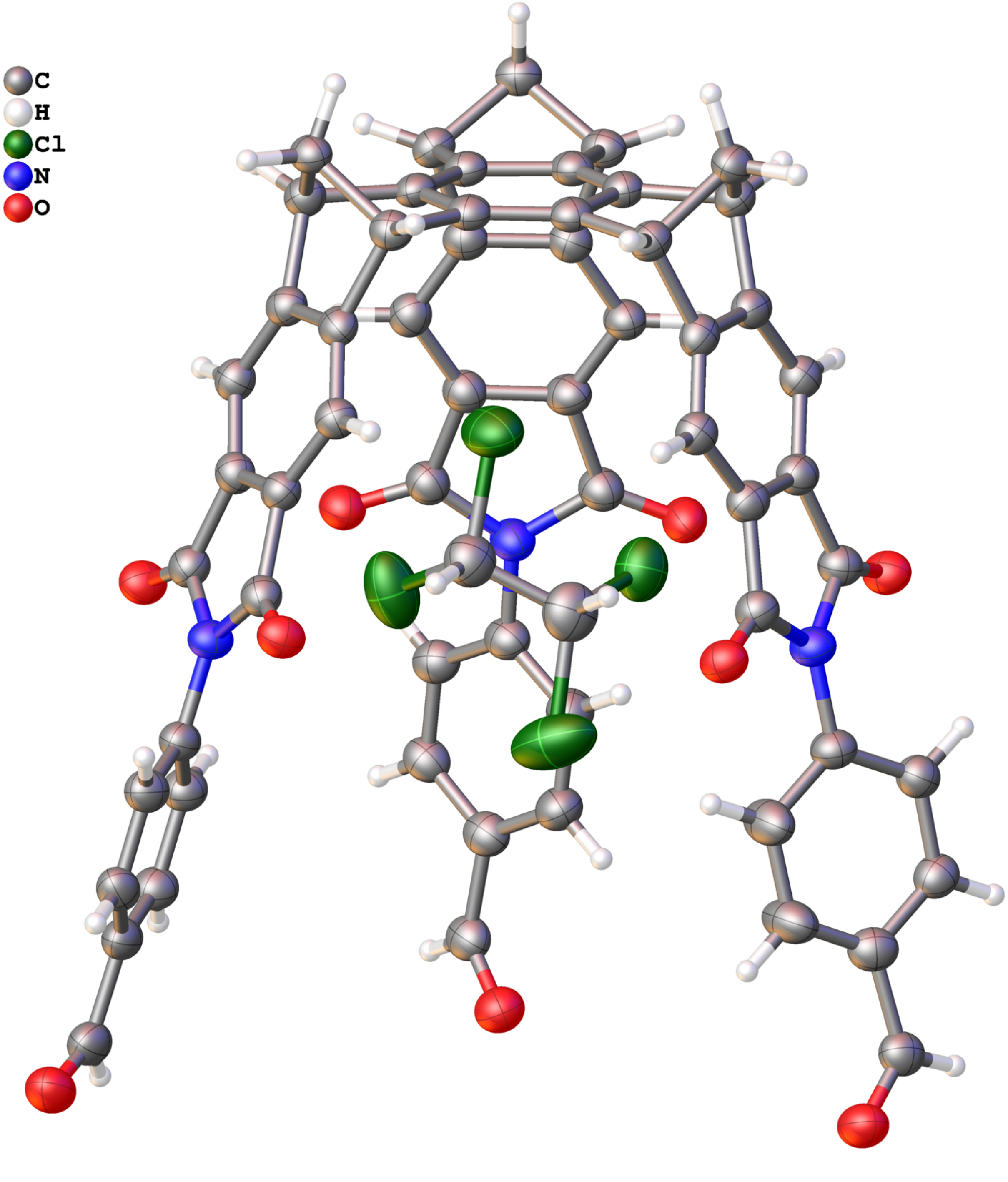
C
H
Cl
N
O

Table 1. Crystal data and structure refinement for *tris*-aldehyde basket **2**.

Report date	2022-01-23	
Identification code	VagAldehyde	
Empirical formula	C62 H35 Cl4 N3 O9	
Molecular formula	C60 H33 N3 O9, C2 H2 Cl4	
Formula weight	1107.73	
Temperature	100.0 K	
Wavelength	1.54178 Å	
Crystal system	Monoclinic	
Space group	P 1 21/c 1	
Unit cell dimensions	a = 14.5572(6) Å	α= 90°.
	b = 22.4891(9) Å	β= 90.014(2)°.
	c = 15.6244(6) Å	γ = 90°.
Volume	5115.1(4) $Å^3$	
Z	4	
Density (calculated)	1.438 Mg/m^3	
Absorption coefficient	2.644 mm^{-1}	
F(000)	2272	
Crystal size	0.322 x 0.034 x 0.026 mm^3	
Crystal color, habit	Colorless Needle	
Theta range for data collection	3.036 to 58.967°.	
Index ranges	-16<=h<=16, -24<=k<=24, -17<=l<=17	
Reflections collected	76602	
Independent reflections	7345 [R(int) = 0.0428, R(sigma) = 0.0207]	
Completeness to theta = 58.967°	99.7 %	

Absorption correction	Semi-empirical from equivalents
Max. and min. transmission	0.1523 and 0.0688
Refinement method	Full-matrix least-squares on F^2
Data / restraints / parameters	7345 / 43 / 741
Goodness-of-fit on F^2	1.028
Final R indices [I>2sigma(I)]	R1 = 0.0557, wR2 = 0.1611
R indices (all data)	R1 = 0.0654, wR2 = 0.1709
Extinction coefficient	n/a
Largest diff. peak and hole	0.469 and -0.842 e.Å^{-3}

Table 2. Atomic coordinates (x 10^4) and equivalent isotropic displacement parameters (Å^{2}x 10^3) for *tris*-aldehyde basket **2**. U(eq) is defined as one third of the trace of the orthogonalized U^{ij} tensor.

	x	y	z	U(eq)
O(1)	8697(2)	4350(1)	4532(2)	45(1)
O(2)	8210(2)	6347(1)	4961(2)	43(1)
O(3)	5105(2)	5305(1)	1725(2)	49(1)
O(4)	5646(2)	7019(1)	7200(2)	41(1)
O(5)	3971(2)	5779(1)	8946(2)	39(1)
O(6)	708(2)	7052(1)	6179(2)	54(1)
O(7)	4902(2)	3288(1)	7824(2)	40(1)
O(8)	7276(2)	2773(1)	6027(1)	40(1)
O(9)	2497(2)	2490(2)	4152(2)	51(1)
O(9B)	3170(9)	2046(7)	3826(8)	60(4)
N(00D)	4600(2)	6400(1)	7900(2)	35(1)
N(00E)	5901(2)	2979(1)	6741(2)	38(1)
N(00F)	8314(2)	5357(1)	4535(2)	40(1)
C(1)	9675(2)	4769(2)	8519(2)	33(1)
C(2)	9483(2)	5384(1)	8644(2)	32(1)
C(3)	8812(2)	5552(1)	9204(2)	32(1)
C(4)	8304(2)	5110(2)	9660(2)	33(1)
C(5)	8476(2)	4520(1)	9519(2)	32(1)
C(6)	9195(2)	4351(1)	8951(2)	33(1)
C(7)	10407(2)	4740(2)	7820(2)	34(1)
C(8)	10966(2)	5311(2)	8047(2)	35(1)

C(9)	10111(2)	5725(2)	8028(2)	33(1)
C(10)	9745(2)	5571(2)	7143(2)	35(1)
C(11)	9916(2)	4960(2)	7015(2)	35(1)
C(12)	9613(2)	4658(2)	6294(2)	37(1)
C(13)	9154(2)	5008(2)	5694(2)	39(1)
C(14)	9004(2)	5612(2)	5817(2)	38(1)
C(15)	9283(2)	5911(2)	6548(2)	37(1)
C(16)	8721(2)	4831(2)	4870(2)	41(1)
C(17)	8476(2)	5844(2)	5084(2)	38(1)
C(18)	7733(2)	5389(2)	3804(2)	39(1)
C(19)	7108(3)	4938(2)	3645(2)	46(1)
C(20)	6499(3)	4994(2)	2973(2)	44(1)
C(21)	6513(2)	5494(2)	2452(2)	40(1)
C(22)	7169(2)	5932(2)	2599(2)	39(1)
C(23)	7782(2)	5884(2)	3273(2)	40(1)
C(24)	5823(3)	5583(2)	1777(2)	41(1)
C(25)	8432(2)	6152(2)	9500(2)	34(1)
C(26)	8243(2)	5998(2)	10450(2)	34(1)
C(27)	7633(2)	5446(2)	10239(2)	34(1)
C(28)	6947(2)	5744(1)	9639(2)	33(1)
C(29)	7444(2)	6172(1)	9168(2)	33(1)
C(30)	7035(2)	6500(1)	8524(2)	34(1)
C(31)	6105(2)	6394(1)	8394(2)	33(1)
C(32)	5606(2)	5998(1)	8886(2)	33(1)
C(33)	6017(2)	5648(1)	9519(2)	33(1)

C(34)	5484(2)	6659(2)	7749(2)	36(1)
C(35)	4628(2)	6019(2)	8621(2)	34(1)
C(36)	3803(2)	6534(2)	7405(2)	36(1)
C(37)	3889(2)	6605(2)	6518(2)	40(1)
C(38)	3116(2)	6733(2)	6037(2)	41(1)
C(39)	2262(2)	6789(2)	6424(2)	42(1)
C(40)	2185(2)	6714(2)	7314(2)	41(1)
C(41)	2950(2)	6588(2)	7802(2)	40(1)
C(42)	1455(3)	6938(2)	5893(3)	45(1)
C(43)	8041(2)	3944(1)	9836(2)	34(1)
C(44)	8925(2)	3549(2)	9891(2)	36(1)
C(45)	9213(2)	3666(1)	8949(2)	35(1)
C(46)	8327(2)	3494(1)	8497(2)	34(1)
C(47)	7597(2)	3664(1)	9046(2)	34(1)
C(48)	6692(2)	3589(1)	8808(2)	35(1)
C(49)	6549(2)	3334(2)	8009(2)	36(1)
C(50)	7264(2)	3176(2)	7471(2)	35(1)
C(51)	8181(2)	3253(1)	7694(2)	34(1)
C(52)	5666(2)	3209(2)	7561(2)	37(1)
C(53)	6876(2)	2946(2)	6651(2)	37(1)
C(54)	5249(2)	2832(2)	6090(2)	38(1)
C(55)	4405(3)	3123(2)	6063(3)	54(1)
C(56)	3770(3)	2972(2)	5444(3)	56(1)
C(57)	3966(2)	2551(2)	4833(2)	42(1)
C(58)	4813(2)	2267(2)	4856(2)	39(1)

C(59)	5451(2)	2400(2)	5481(2)	38(1)
C(60)	3306(7)	2355(4)	4175(7)	40(2)
C(60B)	3240(30)	2511(17)	4160(20)	54(7)
Cl(1S)	7360(1)	5052(1)	7591(1)	69(1)
Cl(2S)	6536(1)	5710(1)	6140(1)	79(1)
Cl(3S)	7153(1)	4278(1)	5918(1)	64(1)
Cl(4S)	5216(1)	4570(1)	5868(1)	98(1)
C(1S)	6389(4)	5131(3)	6927(4)	54(2)
C(2S)	6209(4)	4534(3)	6508(4)	60(2)
C(1SB)	6610(20)	4702(15)	6930(20)	71(9)
C(2SB)	5900(20)	5096(15)	6490(20)	87(10)

Table 3. Bond lengths [Å] and angles [°] for *tris*-aldehyde basket **2**.

O(1)-C(16)	1.205(4)	C(2)-C(9)	1.532(5)
O(2)-C(17)	1.211(4)	C(3)-C(4)	1.428(5)
O(3)-C(24)	1.221(4)	C(3)-C(25)	1.532(5)
O(4)-C(34)	1.204(4)	C(4)-C(5)	1.369(5)
O(5)-C(35)	1.210(4)	C(4)-C(27)	1.530(5)
O(6)-C(42)	1.204(5)	C(5)-C(6)	1.424(5)
O(7)-C(52)	1.199(4)	C(5)-C(43)	1.524(5)
O(8)-C(53)	1.200(4)	C(6)-C(45)	1.540(5)
O(9)-C(60)	1.217(10)	C(7)-H(7)	1.0000
O(9B)-C(60B)	1.18(4)	C(7)-C(8)	1.561(5)
N(00D)-C(34)	1.433(4)	C(7)-C(11)	1.529(5)
N(00D)-C(35)	1.415(4)	C(8)-H(8A)	0.9900
N(00D)-C(36)	1.426(4)	C(8)-H(8B)	0.9900
N(00E)-C(52)	1.423(4)	C(8)-C(9)	1.553(5)
N(00E)-C(53)	1.429(4)	C(9)-H(9)	1.0000
N(00E)-C(54)	1.430(4)	C(9)-C(10)	1.521(5)
N(00F)-C(16)	1.423(5)	C(10)-C(11)	1.411(5)
N(00F)-C(17)	1.411(5)	C(10)-C(15)	1.380(5)
N(00F)-C(18)	1.423(4)	C(11)-C(12)	1.387(5)
C(1)-C(2)	1.425(5)	C(12)-H(12)	0.9500
C(1)-C(6)	1.352(5)	C(12)-C(13)	1.395(5)
C(1)-C(7)	1.528(5)	C(13)-C(14)	1.390(5)
C(2)-C(3)	1.364(5)	C(13)-C(16)	1.486(5)

C(14)-C(15)	1.386(5)	C(29)-C(30)	1.383(5)
C(14)-C(17)	1.475(5)	C(30)-H(30)	0.9500
C(15)-H(15)	0.9500	C(30)-C(31)	1.388(5)
C(18)-C(19)	1.386(5)	C(31)-C(32)	1.383(5)
C(18)-C(23)	1.390(5)	C(31)-C(34)	1.479(5)
C(19)-H(19)	0.9500	C(32)-C(33)	1.397(5)
C(19)-C(20)	1.380(5)	C(32)-C(35)	1.483(5)
C(20)-H(20)	0.9500	C(33)-H(33)	0.9500
C(20)-C(21)	1.388(5)	C(36)-C(37)	1.401(5)
C(21)-C(22)	1.391(5)	C(36)-C(41)	1.393(5)
C(21)-C(24)	1.470(5)	C(37)-H(37)	0.9500
C(22)-H(22)	0.9500	C(37)-C(38)	1.383(5)
C(22)-C(23)	1.384(5)	C(38)-H(38)	0.9500
C(23)-H(23)	0.9500	C(38)-C(39)	1.388(5)
C(24)-H(24)	0.9500	C(39)-C(40)	1.405(5)
C(25)-H(25)	1.0000	C(39)-C(42)	1.477(5)
C(25)-C(26)	1.550(5)	C(40)-H(40)	0.9500
C(25)-C(29)	1.529(4)	C(40)-C(41)	1.379(5)
C(26)-H(26A)	0.9900	C(41)-H(41)	0.9500
C(26)-H(26B)	0.9900	C(42)-H(42)	0.9500
C(26)-C(27)	1.561(5)	C(43)-H(43)	1.0000
C(27)-H(27)	1.0000	C(43)-C(44)	1.566(5)
C(27)-C(28)	1.524(4)	C(43)-C(47)	1.529(4)
C(28)-C(29)	1.411(5)	C(44)-H(44A)	0.9900
C(28)-C(33)	1.384(5)	C(44)-H(44B)	0.9900

C(44)-C(45)	1.553(5)	C(60)-H(60)	0.9500
C(45)-H(45)	1.0000	C(60B)-H(60B)	0.9500
C(45)-C(46)	1.520(5)	Cl(1S)-C(1S)	1.763(6)
C(46)-C(47)	1.419(5)	Cl(1S)-C(1SB)	1.70(3)
C(46)-C(51)	1.383(5)	Cl(2S)-C(1S)	1.802(5)
C(47)-C(48)	1.380(5)	Cl(2S)-C(2SB)	1.75(3)
C(48)-H(48)	0.9500	Cl(3S)-C(2S)	1.754(6)
C(48)-C(49)	1.390(5)	Cl(3S)-C(1SB)	2.01(3)
C(49)-C(50)	1.385(5)	Cl(4S)-C(2S)	1.760(7)
C(49)-C(52)	1.489(5)	Cl(4S)-C(2SB)	1.82(4)
C(50)-C(51)	1.390(5)	C(1S)-H(1S)	1.0000
C(50)-C(53)	1.493(5)	C(1S)-C(2S)	1.516(10)
C(51)-H(51)	0.9500	C(2S)-H(2S)	1.0000
C(54)-C(55)	1.394(5)	C(1SB)-H(1SB)	1.0000
C(54)-C(59)	1.391(5)	C(1SB)-C(2SB)	1.52(5)
C(55)-H(55)	0.9500	C(2SB)-H(2SB)	1.0000
C(55)-C(56)	1.379(6)		
C(56)-H(56)	0.9500	C(35)-N(00D)-C(34)	110.5(3)
C(56)-C(57)	1.374(6)	C(35)-N(00D)-C(36)	125.7(3)
C(57)-C(58)	1.389(5)	C(36)-N(00D)-C(34)	123.8(3)
C(57)-C(60)	1.475(11)	C(52)-N(00E)-C(53)	110.3(3)
C(57)-C(60B)	1.49(4)	C(52)-N(00E)-C(54)	124.4(3)
C(58)-H(58)	0.9500	C(53)-N(00E)-C(54)	125.2(3)
C(58)-C(59)	1.380(5)	C(16)-N(00F)-C(18)	125.8(3)
C(59)-H(59)	0.9500	C(17)-N(00F)-C(16)	110.6(3)

C(17)-N(00F)-C(18)	123.2(3)	C(7)-C(8)-H(8A)	112.9
C(2)-C(1)-C(7)	106.0(3)	C(7)-C(8)-H(8B)	112.9
C(6)-C(1)-C(2)	120.4(3)	H(8A)-C(8)-H(8B)	110.3
C(6)-C(1)-C(7)	133.4(3)	C(9)-C(8)-C(7)	94.1(2)
C(1)-C(2)-C(9)	106.4(3)	C(9)-C(8)-H(8A)	112.9
C(3)-C(2)-C(1)	119.7(3)	C(9)-C(8)-H(8B)	112.9
C(3)-C(2)-C(9)	133.8(3)	C(2)-C(9)-C(8)	99.6(3)
C(2)-C(3)-C(4)	120.0(3)	C(2)-C(9)-H(9)	116.9
C(2)-C(3)-C(25)	134.1(3)	C(8)-C(9)-H(9)	116.9
C(4)-C(3)-C(25)	105.9(3)	C(10)-C(9)-C(2)	104.3(3)
C(3)-C(4)-C(27)	106.5(3)	C(10)-C(9)-C(8)	99.3(3)
C(5)-C(4)-C(3)	119.9(3)	C(10)-C(9)-H(9)	116.9
C(5)-C(4)-C(27)	133.6(3)	C(11)-C(10)-C(9)	106.8(3)
C(4)-C(5)-C(6)	119.6(3)	C(15)-C(10)-C(9)	131.1(3)
C(4)-C(5)-C(43)	134.1(3)	C(15)-C(10)-C(11)	122.0(3)
C(6)-C(5)-C(43)	106.3(3)	C(10)-C(11)-C(7)	106.3(3)
C(1)-C(6)-C(5)	120.3(3)	C(12)-C(11)-C(7)	131.2(3)
C(1)-C(6)-C(45)	133.3(3)	C(12)-C(11)-C(10)	122.4(3)
C(5)-C(6)-C(45)	106.3(3)	C(11)-C(12)-H(12)	122.5
C(1)-C(7)-H(7)	116.9	C(11)-C(12)-C(13)	115.0(3)
C(1)-C(7)-C(8)	99.5(3)	C(13)-C(12)-H(12)	122.5
C(1)-C(7)-C(11)	104.4(3)	C(12)-C(13)-C(16)	129.3(3)
C(8)-C(7)-H(7)	116.9	C(14)-C(13)-C(12)	122.3(3)
C(11)-C(7)-H(7)	116.9	C(14)-C(13)-C(16)	108.4(3)
C(11)-C(7)-C(8)	99.5(3)	C(13)-C(14)-C(17)	108.6(3)

C(15)-C(14)-C(13)	122.9(3)	C(23)-C(22)-H(22)	119.6
C(15)-C(14)-C(17)	128.4(3)	C(18)-C(23)-H(23)	120.6
C(10)-C(15)-C(14)	115.4(3)	C(22)-C(23)-C(18)	118.9(3)
C(10)-C(15)-H(15)	122.3	C(22)-C(23)-H(23)	120.6
C(14)-C(15)-H(15)	122.3	O(3)-C(24)-C(21)	124.3(4)
O(1)-C(16)-N(00F)	125.0(3)	O(3)-C(24)-H(24)	117.9
O(1)-C(16)-C(13)	129.2(3)	C(21)-C(24)-H(24)	117.9
N(00F)-C(16)-C(13)	105.8(3)	C(3)-C(25)-H(25)	116.8
O(2)-C(17)-N(00F)	125.2(3)	C(3)-C(25)-C(26)	98.9(3)
O(2)-C(17)-C(14)	128.3(3)	C(26)-C(25)-H(25)	116.8
N(00F)-C(17)-C(14)	106.5(3)	C(29)-C(25)-C(3)	105.3(3)
C(19)-C(18)-N(00F)	119.8(3)	C(29)-C(25)-H(25)	116.8
C(19)-C(18)-C(23)	120.9(3)	C(29)-C(25)-C(26)	99.5(3)
C(23)-C(18)-N(00F)	119.3(3)	C(25)-C(26)-H(26A)	112.8
C(18)-C(19)-H(19)	120.3	C(25)-C(26)-H(26B)	112.8
C(20)-C(19)-C(18)	119.4(3)	C(25)-C(26)-C(27)	94.4(2)
C(20)-C(19)-H(19)	120.3	H(26A)-C(26)-H(26B)	110.3
C(19)-C(20)-H(20)	119.6	C(27)-C(26)-H(26A)	112.8
C(19)-C(20)-C(21)	120.7(3)	C(27)-C(26)-H(26B)	112.8
C(21)-C(20)-H(20)	119.6	C(4)-C(27)-C(26)	98.8(3)
C(20)-C(21)-C(22)	119.1(3)	C(4)-C(27)-H(27)	116.9
C(20)-C(21)-C(24)	121.4(3)	C(26)-C(27)-H(27)	116.9
C(22)-C(21)-C(24)	119.4(3)	C(28)-C(27)-C(4)	105.8(3)
C(21)-C(22)-H(22)	119.6	C(28)-C(27)-C(26)	98.8(3)
C(23)-C(22)-C(21)	120.9(3)	C(28)-C(27)-H(27)	116.9

C(29)-C(28)-C(27)	106.6(3)	C(41)-C(36)-N(00D)	120.1(3)
C(33)-C(28)-C(27)	131.0(3)	C(41)-C(36)-C(37)	120.7(3)
C(33)-C(28)-C(29)	122.5(3)	C(36)-C(37)-H(37)	120.4
C(28)-C(29)-C(25)	106.6(3)	C(38)-C(37)-C(36)	119.2(3)
C(30)-C(29)-C(25)	131.9(3)	C(38)-C(37)-H(37)	120.4
C(30)-C(29)-C(28)	121.5(3)	C(37)-C(38)-H(38)	119.7
C(29)-C(30)-H(30)	122.1	C(37)-C(38)-C(39)	120.7(3)
C(29)-C(30)-C(31)	115.7(3)	C(39)-C(38)-H(38)	119.7
C(31)-C(30)-H(30)	122.1	C(38)-C(39)-C(40)	119.5(3)
C(30)-C(31)-C(34)	128.7(3)	C(38)-C(39)-C(42)	119.2(3)
C(32)-C(31)-C(30)	122.8(3)	C(40)-C(39)-C(42)	121.3(3)
C(32)-C(31)-C(34)	108.4(3)	C(39)-C(40)-H(40)	119.8
C(31)-C(32)-C(33)	122.0(3)	C(41)-C(40)-C(39)	120.5(3)
C(31)-C(32)-C(35)	109.2(3)	C(41)-C(40)-H(40)	119.8
C(33)-C(32)-C(35)	128.8(3)	C(36)-C(41)-H(41)	120.3
C(28)-C(33)-C(32)	115.3(3)	C(40)-C(41)-C(36)	119.4(3)
C(28)-C(33)-H(33)	122.3	C(40)-C(41)-H(41)	120.3
C(32)-C(33)-H(33)	122.3	O(6)-C(42)-C(39)	123.9(4)
O(4)-C(34)-N(00D)	124.6(3)	O(6)-C(42)-H(42)	118.0
O(4)-C(34)-C(31)	129.6(3)	C(39)-C(42)-H(42)	118.0
N(00D)-C(34)-C(31)	105.9(3)	C(5)-C(43)-H(43)	116.8
O(5)-C(35)-N(00D)	125.4(3)	C(5)-C(43)-C(44)	99.1(3)
O(5)-C(35)-C(32)	128.9(3)	C(5)-C(43)-C(47)	105.3(3)
N(00D)-C(35)-C(32)	105.7(3)	C(44)-C(43)-H(43)	116.8
C(37)-C(36)-N(00D)	119.2(3)	C(47)-C(43)-H(43)	116.8

C(47)-C(43)-C(44)	99.0(3)	C(49)-C(50)-C(51)	122.5(3)
C(43)-C(44)-H(44A)	112.9	C(49)-C(50)-C(53)	109.0(3)
C(43)-C(44)-H(44B)	112.9	C(51)-C(50)-C(53)	128.4(3)
H(44A)-C(44)-H(44B)	110.3	C(46)-C(51)-C(50)	115.1(3)
C(45)-C(44)-C(43)	94.2(2)	C(46)-C(51)-H(51)	122.5
C(45)-C(44)-H(44A)	112.9	C(50)-C(51)-H(51)	122.5
C(45)-C(44)-H(44B)	112.9	O(7)-C(52)-N(00E)	125.8(3)
C(6)-C(45)-C(44)	99.4(3)	O(7)-C(52)-C(49)	127.7(3)
C(6)-C(45)-H(45)	117.0	N(00E)-C(52)-C(49)	106.5(3)
C(44)-C(45)-H(45)	117.0	O(8)-C(53)-N(00E)	125.4(3)
C(46)-C(45)-C(6)	103.9(3)	O(8)-C(53)-C(50)	128.7(3)
C(46)-C(45)-C(44)	99.7(3)	N(00E)-C(53)-C(50)	105.9(3)
C(46)-C(45)-H(45)	117.0	C(55)-C(54)-N(00E)	119.9(3)
C(47)-C(46)-C(45)	106.6(3)	C(59)-C(54)-N(00E)	120.6(3)
C(51)-C(46)-C(45)	130.7(3)	C(59)-C(54)-C(55)	119.5(3)
C(51)-C(46)-C(47)	122.6(3)	C(54)-C(55)-H(55)	120.1
C(46)-C(47)-C(43)	106.4(3)	C(56)-C(55)-C(54)	119.8(4)
C(48)-C(47)-C(43)	132.2(3)	C(56)-C(55)-H(55)	120.1
C(48)-C(47)-C(46)	121.3(3)	C(55)-C(56)-H(56)	119.4
C(47)-C(48)-H(48)	122.1	C(57)-C(56)-C(55)	121.1(4)
C(47)-C(48)-C(49)	115.8(3)	C(57)-C(56)-H(56)	119.4
C(49)-C(48)-H(48)	122.1	C(56)-C(57)-C(58)	118.9(3)
C(48)-C(49)-C(52)	129.0(3)	C(56)-C(57)-C(60)	123.7(4)
C(50)-C(49)-C(48)	122.6(3)	C(56)-C(57)-C(60B)	112.4(15)
C(50)-C(49)-C(52)	108.4(3)	C(58)-C(57)-C(60)	117.3(4)

C(58)-C(57)-C(60B)	128.4(15)
C(57)-C(58)-H(58)	119.5
C(59)-C(58)-C(57)	121.1(3)
C(59)-C(58)-H(58)	119.5
C(54)-C(59)-H(59)	120.2
C(58)-C(59)-C(54)	119.6(3)
C(58)-C(59)-H(59)	120.2
O(9)-C(60)-C(57)	125.2(8)
O(9)-C(60)-H(60)	117.4
C(57)-C(60)-H(60)	117.4
O(9B)-C(60B)-C(57)	115(3)
O(9B)-C(60B)-H(60B)	122.3
C(57)-C(60B)-H(60B)	122.3
Cl(1S)-C(1S)-Cl(2S)	112.3(3)
Cl(1S)-C(1S)-H(1S)	108.4
Cl(2S)-C(1S)-H(1S)	108.4
C(2S)-C(1S)-Cl(1S)	107.6(4)
C(2S)-C(1S)-Cl(2S)	111.5(4)
C(2S)-C(1S)-H(1S)	108.4
Cl(3S)-C(2S)-Cl(4S)	111.1(4)
Cl(3S)-C(2S)-H(2S)	107.6
Cl(4S)-C(2S)-H(2S)	107.6
C(1S)-C(2S)-Cl(3S)	112.5(4)
C(1S)-C(2S)-Cl(4S)	110.2(4)
C(1S)-C(2S)-H(2S)	107.6
Cl(1S)-C(1SB)-Cl(3S)	116.2(16)
Cl(1S)-C(1SB)-H(1SB)	107.6
Cl(3S)-C(1SB)-H(1SB)	107.6
C(2SB)-C(1SB)-Cl(1S)	116(3)
C(2SB)-C(1SB)-Cl(3S)	101(2)
C(2SB)-C(1SB)-H(1SB)	107.6
Cl(2S)-C(2SB)-Cl(4S)	129.2(19)
Cl(2S)-C(2SB)-H(2SB)	106.2
Cl(4S)-C(2SB)-H(2SB)	106.2
C(1SB)-C(2SB)-Cl(2S)	104(2)
C(1SB)-C(2SB)-Cl(4S)	103(2)
C(1SB)-C(2SB)-H(2SB)	106.

Table 4. Anisotropic displacement parameters (Å^{2}x 10^3) for *tris*-aldehyde basket **2**. The anisotropic displacement factor exponent takes the form: $-2\pi^2[h^2a^{*2}U^{11} + ... + 2 h k a^*b^*U^{12}]$

	U^{11}	U^{22}	U^{33}	U^{23}	U^{13}	U^{12}
O(1)	53(2)	42(2)	40(1)	-7(1)	-8(1)	6(1)
O(2)	46(1)	44(2)	40(1)	-1(1)	-6(1)	5(1)
O(3)	49(2)	49(2)	49(2)	-5(1)	-7(1)	-1(1)
O(4)	42(1)	42(1)	37(1)	8(1)	-3(1)	-3(1)
O(5)	35(1)	44(1)	38(1)	5(1)	0(1)	-1(1)
O(6)	43(2)	49(2)	68(2)	2(1)	-10(1)	-1(1)
O(7)	37(1)	47(1)	38(1)	-6(1)	0(1)	-2(1)
O(8)	40(1)	49(1)	33(1)	-5(1)	0(1)	2(1)
O(9)	41(2)	55(2)	57(2)	-9(2)	-8(2)	1(2)
O(9B)	60(7)	70(8)	50(6)	-2(5)	-4(5)	-16(5)
N(00D)	35(2)	39(2)	30(1)	4(1)	-3(1)	-1(1)
N(00E)	38(2)	45(2)	32(2)	-5(1)	-4(1)	1(1)
N(00F)	44(2)	43(2)	32(2)	-3(1)	-5(1)	5(1)
C(1)	30(2)	38(2)	30(2)	-3(1)	-6(1)	1(1)
C(2)	30(2)	38(2)	28(2)	-3(1)	-4(1)	-2(1)
C(3)	31(2)	35(2)	31(2)	-2(1)	-8(1)	-3(1)
C(4)	33(2)	38(2)	28(2)	0(1)	-6(1)	0(1)
C(5)	32(2)	37(2)	27(2)	0(1)	-6(1)	-1(1)
C(6)	32(2)	34(2)	32(2)	-2(1)	-5(1)	0(1)
C(7)	31(2)	42(2)	30(2)	-3(1)	-1(1)	2(1)
C(8)	32(2)	42(2)	31(2)	0(1)	1(1)	-1(1)

C(9)	33(2)	35(2)	31(2)	-2(1)	0(1)	-4(1)
C(10)	30(2)	41(2)	34(2)	0(2)	1(1)	-1(1)
C(11)	34(2)	38(2)	33(2)	1(1)	1(1)	1(1)
C(12)	38(2)	39(2)	34(2)	-3(2)	-4(2)	3(2)
C(13)	38(2)	45(2)	34(2)	-6(2)	-4(2)	3(2)
C(14)	38(2)	40(2)	36(2)	-1(2)	-1(2)	3(2)
C(15)	37(2)	38(2)	37(2)	-3(2)	2(2)	0(2)
C(16)	43(2)	43(2)	35(2)	-1(2)	-3(2)	5(2)
C(17)	38(2)	39(2)	36(2)	0(2)	0(2)	3(2)
C(18)	40(2)	44(2)	32(2)	-4(2)	-3(2)	5(2)
C(19)	51(2)	45(2)	41(2)	1(2)	-7(2)	2(2)
C(20)	46(2)	45(2)	43(2)	-6(2)	-6(2)	-1(2)
C(21)	42(2)	47(2)	32(2)	-3(2)	1(2)	5(2)
C(22)	42(2)	46(2)	31(2)	2(2)	2(2)	6(2)
C(23)	38(2)	47(2)	35(2)	-3(2)	1(2)	1(2)
C(24)	44(2)	44(2)	35(2)	-7(2)	-1(2)	8(2)
C(25)	32(2)	35(2)	34(2)	-2(1)	0(1)	-1(1)
C(26)	34(2)	38(2)	32(2)	-3(1)	-4(1)	3(1)
C(27)	34(2)	38(2)	28(2)	0(1)	0(1)	0(1)
C(28)	37(2)	33(2)	28(2)	-5(1)	-2(1)	3(1)
C(29)	33(2)	35(2)	31(2)	-4(1)	0(1)	0(1)
C(30)	35(2)	33(2)	33(2)	-2(1)	2(1)	-1(1)
C(31)	36(2)	32(2)	32(2)	-1(1)	0(1)	2(1)
C(32)	33(2)	33(2)	33(2)	-3(1)	-1(1)	1(1)
C(33)	34(2)	35(2)	31(2)	0(1)	1(1)	-1(1)

C(34)	38(2)	34(2)	35(2)	-2(2)	-2(2)	0(2)
C(35)	39(2)	34(2)	30(2)	-2(1)	-2(2)	2(2)
C(36)	36(2)	33(2)	38(2)	2(1)	-6(2)	-1(1)
C(37)	42(2)	41(2)	38(2)	1(2)	-4(2)	-1(2)
C(38)	44(2)	42(2)	38(2)	2(2)	-6(2)	-1(2)
C(39)	45(2)	30(2)	49(2)	2(2)	-12(2)	-3(2)
C(40)	34(2)	36(2)	52(2)	3(2)	-2(2)	-1(2)
C(41)	41(2)	36(2)	41(2)	5(2)	-4(2)	-4(2)
C(42)	46(2)	36(2)	54(2)	2(2)	-11(2)	-5(2)
C(43)	36(2)	35(2)	30(2)	-1(1)	-1(1)	-3(1)
C(44)	38(2)	34(2)	36(2)	2(1)	-6(2)	-1(1)
C(45)	36(2)	35(2)	34(2)	-3(1)	-3(1)	1(1)
C(46)	38(2)	31(2)	34(2)	3(1)	-4(1)	-2(1)
C(47)	41(2)	30(2)	30(2)	2(1)	0(1)	-3(1)
C(48)	37(2)	35(2)	32(2)	2(1)	1(1)	-2(1)
C(49)	39(2)	36(2)	33(2)	2(1)	-2(2)	-3(2)
C(50)	37(2)	35(2)	33(2)	1(1)	-2(2)	0(1)
C(51)	37(2)	34(2)	32(2)	2(1)	1(1)	-2(1)
C(52)	37(2)	38(2)	36(2)	-2(2)	-1(2)	-1(2)
C(53)	36(2)	40(2)	36(2)	1(2)	-3(2)	0(2)
C(54)	39(2)	43(2)	34(2)	-1(2)	-6(2)	-2(2)
C(55)	53(2)	64(3)	46(2)	-17(2)	-9(2)	18(2)
C(56)	47(2)	74(3)	49(2)	-11(2)	-11(2)	17(2)
C(57)	41(2)	49(2)	37(2)	3(2)	-1(1)	-4(2)
C(58)	46(2)	40(2)	33(2)	1(2)	-2(2)	-3(2)

C(59)	37(2)	39(2)	38(2)	0(2)	-1(2)	1(2)
C(60)	40(3)	40(5)	39(3)	5(3)	-1(2)	-2(2)
C(60B)	52(9)	67(9)	44(9)	4(6)	-11(8)	-9(6)
Cl(1S)	70(1)	83(1)	55(1)	-14(1)	-14(1)	9(1)
Cl(2S)	83(1)	61(1)	94(1)	19(1)	29(1)	12(1)
Cl(3S)	77(1)	54(1)	60(1)	0(1)	10(1)	-2(1)
Cl(4S)	60(1)	151(1)	83(1)	-36(1)	-1(1)	-17(1)
C(1S)	58(4)	51(4)	53(4)	4(2)	0(3)	0(3)
C(2S)	67(4)	56(4)	57(4)	8(3)	8(3)	-6(3)
C(1SB)	76(10)	76(10)	59(10)	-3(6)	-8(6)	-4(5)
C(2SB)	89(11)	106(11)	66(11)	11(5)	-10(5)	8(6)

Table 5. Hydrogen coordinates (x 10^4) and isotropic displacement parameters ($Å^2$x 10^3) for *tris*-aldehyde basket **2**.

	x	y	z	U(eq)
H(7)	10761	4360	7770	41
H(8A)	11257	5290	8619	42
H(8B)	11427	5415	7606	42
H(9)	10217	6156	8153	40
H(12)	9711	4244	6215	44
H(15)	9165	6323	6633	45
H(19)	7099	4592	3995	55
H(20)	6064	4688	2866	53
H(22)	7198	6268	2231	47
H(23)	8228	6185	3372	48
H(24)	5943	5877	1355	49
H(25)	8821	6510	9387	41
H(26A)	7902	6314	10755	41
H(26B)	8807	5892	10769	41
H(27)	7373	5221	10736	40
H(30)	7368	6779	8190	40
H(33)	5681	5365	9844	40
H(37)	4471	6566	6249	48
H(38)	3170	6783	5435	50
H(40)	1602	6751	7582	49

H(41)	2896	6538	8404	47
H(42)	1529	6943	5289	54
H(43)	7654	3971	10364	41
H(44A)	9378	3700	10311	44
H(44B)	8786	3125	10005	44
H(45)	9790	3471	8746	42
H(48)	6197	3705	9168	42
H(51)	8671	3147	7322	41
H(55)	4267	3424	6469	65
H(56)	3188	3163	5440	68
H(58)	4955	1977	4435	47
H(59)	6024	2198	5494	46
H(60)	3531	2103	3734	48
H(60B)	2858	2839	4021	65
H(1S)	5849	5234	7293	65
H(2S)	6090	4240	6975	72
H(1SB)	6265	4401	7275	85
H(2SB)	5485	5246	6955	105

Experimental Summary for CBC 5 (CCDC 2143948)

The single crystal X-ray diffraction studies were carried out on a Bruker Kappa Photon III CPAD diffractometer equipped with Mo K_α radiation (λ = 0.71073 Å). A 0.091 x 0.078 x 0.063 mm colorless block was mounted on a Cryoloop with Paratone 24EX oil. Data were collected in a nitrogen gas stream at 100(2) K using ϕ and ϖ scans. Crystal-to-detector distance was 100 mm using variable exposure time (60s-300s) depending on θ with a scan width of 1.0°. Data collection was 99.7% complete to 15.920° in θ (1.30Å). A total of 192739 reflections were collected covering the indices, -38<=h<=38, -38<=k<=38, -17<=l<=17. 7007 reflections were found to be symmetry independent, with a R_{int} of 0.1969. Indexing and unit cell refinement indicated a body centered, tetragonal lattice. The space group was found to be $I4_1/a$. The data were integrated using the Bruker SAINT software program and scaled using the SADABS software program. Solution by direct methods (SHELXT) produced a complete phasing model for refinement.

All nonhydrogen atoms were refined anisotropically by full-matrix least-squares (SHELXL-2014). All hydrogen atoms were placed using a riding model. Their positions were constrained relative to their parent atom using the appropriate HFIX command in SHELXL-2014. Due to unmodelable solvent disorder, OLEX2 SMTBX was used to mask out electron density from the lattice due to the disordered solvent. Solvent appeared to be a mixture of methanol and 1,1,2,2-Tetrachloroethane. One large void was found to contain approximately 5350 electrons. Crystallographic data are summarized in Table 1.

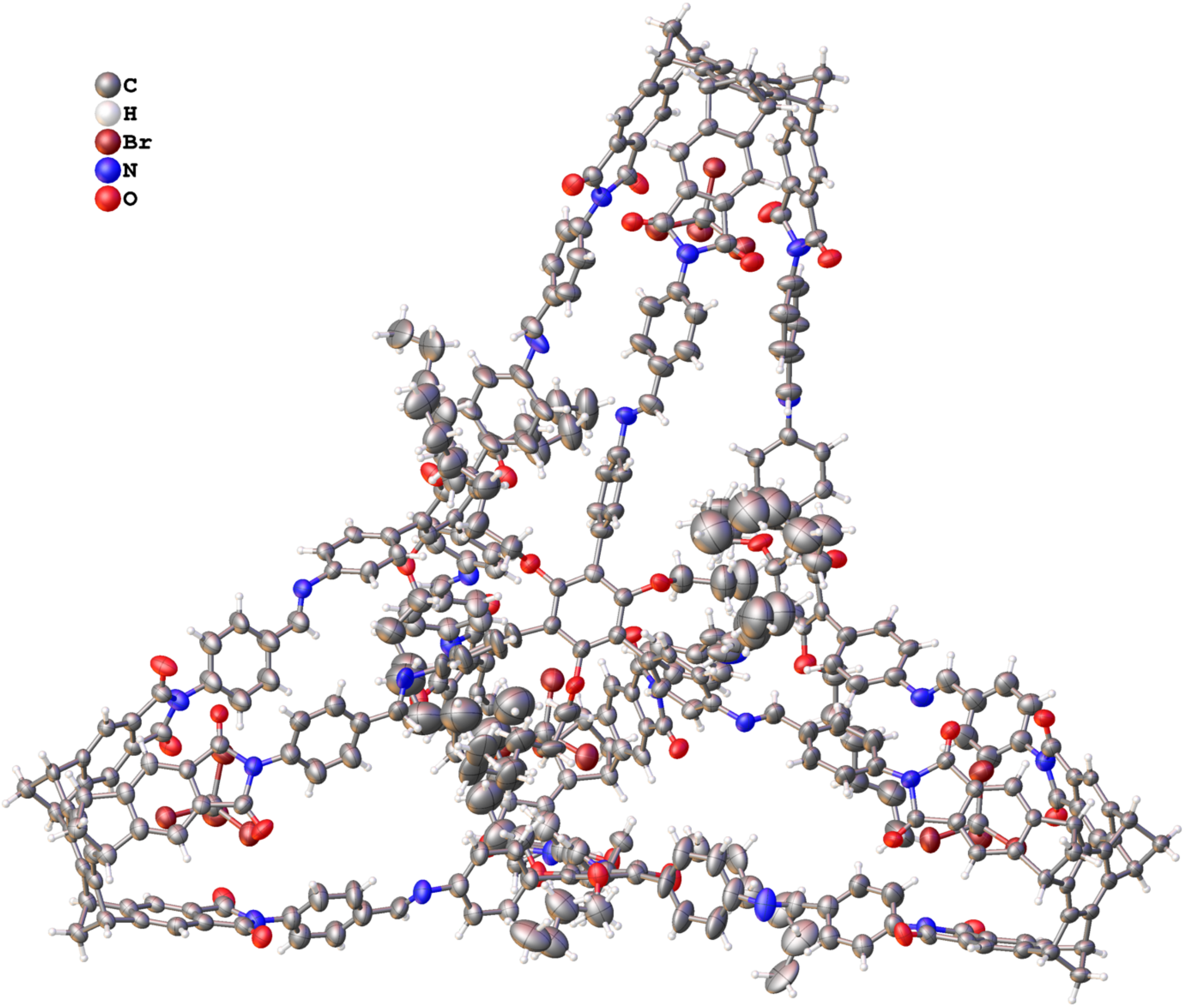
C
H
Br
N
O

Table 1. Crystal data and structure refinement for CBC **5**.

Report date	2022-01-23	
Identification code	VagLrgCage	
Empirical formula	C412 H336 Br16 N24 O36	
Molecular formula	C408 H336 N24 O36, 4(Br4 C), [+ solvents]	
Formula weight	7477.59	
Temperature	100.00 K	
Wavelength	0.71073 Å	
Crystal system	Tetragonal	
Space group	$I4_1/a$	
Unit cell dimensions	a = 50.408(5) Å	α= 90°.
	b = 50.408(5) Å	β= 90°.
	c = 22.984(3) Å	γ = 90°.
Volume	58402(13) $Å^3$	
Z	4	
Density (calculated)	0.850 Mg/m^3	
Absorption coefficient	1.141 mm^{-1}	
F(000)	15296	
Crystal size	0.091 x 0.078 x 0.063 mm^3	
Crystal color, habit	Colorless Block	
Theta range for data collection	1.807 to 15.920°.	
Index ranges	-38<=h<=38, -38<=k<=38, -17<=l<=17	
Reflections collected	192739	
Independent reflections	7007 [R(int) = 0.1969, R(sigma) = 0.0636]	
Completeness to theta = 15.920°	99.7 %	

Absorption correction	Semi-empirical from equivalents
Max. and min. transmission	0.0797 and 0.0636
Refinement method	Full-matrix least-squares on F^2
Data / restraints / parameters	7007 / 2245 / 973
Goodness-of-fit on F^2	1.722
Final R indices [I>2sigma(I)]	R1 = 0.1685, wR2 = 0.4278
R indices (all data)	R1 = 0.2289, wR2 = 0.4743
Extinction coefficient	n/a
Largest diff. peak and hole	0.968 and -0.652 e.Å^{-3}

Table 2. Atomic coordinates (x 10^4) and equivalent isotropic displacement parameters ($Å^2$x 10^3) for CBC **5**. U(eq) is defined as one third of the trace of the orthogonalized U^{ij} tensor.

	x	y	z	U(eq)
O(1)	5292(4)	4372(3)	3022(9)	151(8)
O(2)	5153(4)	5077(4)	4209(9)	166(9)
N(1)	5180(5)	4665(3)	3742(8)	151(8)
N(2)	4418(4)	4081(4)	5531(10)	127(8)
C(1)	6133(4)	5454(5)	2552(9)	108(8)
C(2)	6176(3)	5238(5)	2190(11)	111(8)
C(3)	5911(4)	5177(4)	1899(8)	118(8)
C(4)	5805(4)	5457(4)	1834(7)	123(9)
C(5)	5842(4)	5525(3)	2490(8)	108(8)
C(6)	5688(3)	5294(2)	2744(6)	101(8)
C(7)	5734(3)	5080(3)	2378(6)	107(8)
C(8)	5618(3)	4835(3)	2498(6)	121(9)
C(9)	5458(3)	4805(2)	2985(7)	114(8)
C(10)	5412(3)	5020(3)	3352(6)	117(8)
C(11)	5527(3)	5264(3)	3231(6)	106(8)
C(12)	5300(6)	4580(4)	3208(11)	133(10)
C(13)	5255(5)	4939(4)	3873(9)	125(10)
C(14)	5062(4)	4507(4)	4179(8)	169(11)
C(15)	5135(4)	4245(4)	4270(10)	202(13)
C(16)	4981(5)	4082(3)	4623(11)	199(13)
C(17)	4754(4)	4183(4)	4885(10)	190(12)

C(18)	4681(4)	4445(4)	4794(10)	171(12)
C(19)	4835(5)	4608(3)	4442(9)	169(11)
C(20)	4602(6)	4003(5)	5239(14)	165(12)
C(21)	4271(3)	3896(3)	5882(8)	113(9)
C(22)	4031(4)	3807(4)	5658(6)	109(9)
C(23)	3884(3)	3618(4)	5961(9)	113(9)
C(24)	3977(4)	3519(3)	6487(9)	122(9)
C(25)	4217(4)	3609(4)	6711(7)	138(10)
C(26)	4364(3)	3798(4)	6408(9)	134(10)
O(1')	7048(4)	4220(4)	3957(9)	149(8)
O(2')	6368(4)	3898(4)	2853(9)	163(9)
N(1')	6724(4)	3975(3)	3501(9)	157(8)
N(2')	6559(6)	3091(4)	5391(9)	148(9)
C(1')	6425(4)	5135(4)	2186(11)	112(8)
C(2')	6633(5)	5236(4)	2514(10)	119(8)
C(3')	6880(4)	5074(3)	2370(9)	117(8)
C(4')	6830(4)	5025(4)	1730(8)	123(9)
C(5')	6545(4)	4913(4)	1827(8)	122(8)
C(6')	6618(3)	4699(3)	2256(6)	118(8)
C(7')	6823(3)	4800(2)	2592(7)	114(8)
C(8')	6925(3)	4653(3)	3051(7)	131(9)
C(9')	6821(3)	4404(3)	3175(6)	130(9)
C(10')	6615(3)	4302(2)	2839(7)	123(9)
C(11')	6514(3)	4450(3)	2379(6)	110(8)
C(12')	6880(6)	4207(5)	3629(11)	137(10)

C(13')	6526(5)	4038(4)	3054(12)	144(10)
C(14')	6687(5)	3749(3)	3847(9)	165(10)
C(15')	6652(5)	3766(3)	4446(9)	206(13)
C(16')	6617(6)	3537(5)	4773(7)	210(13)
C(17')	6617(6)	3290(4)	4501(9)	192(12)
C(18')	6652(5)	3273(3)	3903(9)	202(13)
C(19')	6687(5)	3503(4)	3576(7)	184(12)
C(20')	6568(8)	3066(4)	4869(10)	170(12)
C(21')	6496(4)	2863(3)	5745(8)	158(10)
C(22')	6605(4)	2623(4)	5569(7)	137(10)
C(23')	6564(4)	2395(3)	5899(10)	142(11)
C(24')	6413(5)	2408(4)	6405(9)	142(11)
C(25')	6304(4)	2648(5)	6581(7)	143(10)
C(26')	6346(4)	2876(3)	6251(9)	154(11)
O(1")	5919(4)	5306(4)	5463(8)	143(8)
O(2")	6754(4)	4967(4)	5419(9)	154(8)
N(1")	6303(4)	5075(4)	5601(7)	133(8)
N(2")	5870(5)	4539(6)	7905(11)	206(12)
C(1")	6575(5)	5443(4)	2867(9)	117(8)
C(2")	6326(5)	5559(5)	2884(9)	119(8)
C(3")	6333(4)	5765(4)	3367(8)	116(8)
C(4")	6615(4)	5863(4)	3298(9)	126(9)
C(5")	6737(4)	5578(4)	3335(8)	137(8)
C(6")	6617(3)	5499(3)	3912(6)	139(9)
C(7")	6364(3)	5609(3)	3921(6)	134(9)

C(8")	6190(3)	5548(3)	4370(7)	116(9)
C(9")	6269(3)	5376(3)	4811(6)	116(8)
C(10")	6521(3)	5266(3)	4802(6)	115(8)
C(11")	6695(3)	5327(3)	4352(7)	131(9)
C(12")	6128(4)	5263(6)	5319(10)	124(10)
C(13")	6563(4)	5084(6)	5309(10)	133(10)
C(14")	6270(4)	4949(4)	6143(7)	162(10)
C(15")	6144(4)	5071(3)	6609(9)	161(11)
C(16")	6062(4)	4922(4)	7084(7)	171(11)
C(17")	6105(4)	4649(4)	7092(8)	161(11)
C(18")	6231(5)	4527(3)	6626(9)	192(12)
C(19")	6313(5)	4676(4)	6151(8)	212(13)
C(20")	6075(5)	4516(6)	7644(10)	167(12)
C(21")	5811(5)	4369(5)	8390(8)	218(12)
C(22")	5903(5)	4453(5)	8929(11)	251(15)
C(23")	5831(6)	4317(6)	9431(8)	241(15)
C(24")	5667(6)	4096(6)	9394(10)	207(14)
C(25")	5574(5)	4012(5)	8855(14)	226(14)
C(26")	5646(5)	4148(5)	8353(10)	210(13)
Br(1S)	6210(1)	4899(1)	3530(2)	159(2)
Br(4S)	5978(1)	4327(1)	3600(2)	210(2)
Br(2S)	6412(1)	4518(1)	4484(2)	191(2)
Br(3S)	5835(1)	4746(1)	4556(2)	203(2)
C(1S)	6110(7)	4623(7)	4024(15)	159(2)
O(3)	3719(5)	3585(4)	7567(10)	168(8)

C(33)	3885(6)	3646(6)	8050(14)	216(13)
C(34)	3822(6)	3907(6)	8358(15)	223(14)
C(35)	3527(7)	3924(6)	8470(20)	285(17)
C(36)	3446(7)	4207(5)	8640(20)	325(19)
C(37)	3161(8)	4206(8)	8874(18)	350(20)
C(38)	2963(7)	4243(8)	8370(20)	330(20)
O(4)	3398(4)	2687(4)	7566(9)	158(8)
C(39)	3120(5)	2681(6)	7447(15)	211(13)
C(40)	2965(6)	2475(6)	7800(14)	271(16)
C(41)	2966(8)	2208(6)	7500(16)	303(18)
C(42)	2820(7)	1995(7)	7856(17)	321(19)
C(43)	2540(7)	1940(7)	7620(20)	330(20)
C(44)	2463(11)	1646(8)	7710(30)	390(30)
O(5)	3894(5)	3020(4)	5928(8)	146(8)
C(45)	4148(5)	2896(8)	5903(16)	236(15)
C(46)	4321(6)	2980(10)	5389(13)	345(19)
C(47)	4587(6)	2843(9)	5420(18)	430(20)
C(48)	4806(6)	3037(8)	5610(30)	500(30)
C(49)	5078(6)	2897(13)	5620(20)	530(30)
C(50)	5164(11)	2834(16)	6250(30)	560(40)
C(27)	3829(4)	3295(4)	6791(11)	122(8)
C(28)	3721(5)	3332(4)	7342(11)	138(9)
C(29)	3568(4)	3133(5)	7594(7)	122(9)
C(30)	3524(4)	2897(4)	7297(11)	114(9)
C(31)	3631(4)	2860(4)	6746(11)	114(9)

C(32)	3784(4)	3059(5)	6493(7)	130(9)

Table 3. Bond lengths [Å] and angles [°] for CBC **5**.

O(1)-C(12)	1.135(17)	C(8)-H(8)	0.9500
O(2)-C(13)	1.160(17)	C(8)-C(9)	1.3900
N(1)-C(12)	1.435(14)	C(9)-C(10)	1.3900
N(1)-C(13)	1.462(14)	C(9)-C(12)	1.476(14)
N(1)-C(14)	1.412(11)	C(10)-C(11)	1.3900
N(2)-C(20)	1.212(15)	C(10)-C(13)	1.493(14)
N(2)-C(21)	1.437(17)	C(11)-H(11)	0.9500
C(1)-C(2)	1.388(11)	C(14)-C(15)	1.3900
C(1)-C(5)	1.518(15)	C(14)-C(19)	1.3900
C(1)-C(2")	1.345(16)	C(15)-H(15)	0.9500
C(2)-C(3)	1.526(15)	C(15)-C(16)	1.3900
C(2)-C(1')	1.360(16)	C(16)-H(16)	0.9500
C(3)-H(3)	1.0000	C(16)-C(17)	1.3900
C(3)-C(4)	1.516(15)	C(17)-C(18)	1.3900
C(3)-C(7)	1.500(14)	C(17)-C(20)	1.436(17)
C(4)-H(4A)	0.9900	C(18)-H(18)	0.9500
C(4)-H(4B)	0.9900	C(18)-C(19)	1.3900
C(4)-C(5)	1.559(15)	C(19)-H(19)	0.9500
C(5)-H(5)	1.0000	C(20)-H(20)	0.9500
C(5)-C(6)	1.513(14)	C(21)-C(22)	1.3900
C(6)-C(7)	1.3900	C(21)-C(26)	1.3900
C(6)-C(11)	1.3900	C(22)-H(22)	0.9500
C(7)-C(8)	1.3900	C(22)-C(23)	1.3900

C(23)-H(23)	0.9500	C(5')-C(6')	1.507(15)
C(23)-C(24)	1.3900	C(6')-C(7')	1.3900
C(24)-C(25)	1.3900	C(6')-C(11')	1.3900
C(24)-C(27)	1.528(19)	C(7')-C(8')	1.3900
C(25)-H(25)	0.9500	C(8')-H(8')	0.9500
C(25)-C(26)	1.3900	C(8')-C(9')	1.3900
C(26)-H(26)	0.9500	C(9')-C(10')	1.3900
O(1')-C(12')	1.133(18)	C(9')-C(12')	1.471(15)
O(2')-C(13')	1.160(18)	C(10')-C(11')	1.3900
N(1')-C(12')	1.440(15)	C(10')-C(13')	1.490(15)
N(1')-C(13')	1.467(15)	C(11')-H(11')	0.9500
N(1')-C(14')	1.402(12)	C(14')-C(15')	1.3900
N(2')-C(20')	1.208(16)	C(14')-C(19')	1.3900
N(2')-C(21')	1.445(17)	C(15')-H(15')	0.9500
C(1')-C(2')	1.386(12)	C(15')-C(16')	1.3900
C(1')-C(5')	1.517(17)	C(16')-H(16')	0.9500
C(2')-C(3')	1.523(17)	C(16')-C(17')	1.3900
C(2')-C(1'')	1.354(16)	C(17')-C(18')	1.3900
C(3')-H(3')	1.0000	C(17')-C(20')	1.433(17)
C(3')-C(4')	1.515(16)	C(18')-H(18')	0.9500
C(3')-C(7')	1.499(15)	C(18')-C(19')	1.3900
C(4')-H(4'A)	0.9900	C(19')-H(19')	0.9500
C(4')-H(4'B)	0.9900	C(20')-H(20')	0.9500
C(4')-C(5')	1.561(17)	C(21')-C(22')	1.3900
C(5')-H(5')	1.0000	C(21')-C(26')	1.3900

C(22')-H(22')	0.9500	C(5")-H(5")	1.0000
C(22')-C(23')	1.3900	C(5")-C(6")	1.513(15)
C(23')-H(23')	0.9500	C(6")-C(7")	1.3900
C(23')-C(24')	1.3900	C(6")-C(11")	1.3900
C(24')-C(25')	1.3900	C(7")-C(8")	1.3900
C(24')-C(31)#1	1.58(19)	C(8")-H(8")	0.9500
C(25')-H(25')	0.9500	C(8")-C(9")	1.3900
C(25')-C(26')	1.3900	C(9")-C(10")	1.3900
C(26')-H(26')	0.9500	C(9")-C(12")	1.480(14)
O(1")-C(12")	1.129(17)	C(10")-C(11")	1.3900
O(2")-C(13")	1.159(17)	C(10")-C(13")	1.496(14)
N(1")-C(12")	1.448(14)	C(11")-H(11")	0.9500
N(1")-C(13")	1.469(14)	C(14")-C(15")	1.3900
N(1")-C(14")	1.408(11)	C(14")-C(19")	1.3900
N(2")-C(20")	1.202(16)	C(15")-H(15")	0.9500
N(2")-C(21")	1.438(18)	C(15")-C(16")	1.3900
C(1")-C(2")	1.386(12)	C(16")-H(16")	0.9500
C(1")-C(5")	1.514(17)	C(16")-C(17")	1.3900
C(2")-C(3")	1.519(17)	C(17")-C(18")	1.3900
C(3")-H(3")	1.0000	C(17")-C(20")	1.442(16)
C(3")-C(4")	1.512(16)	C(18")-H(18")	0.9500
C(3")-C(7")	1.503(15)	C(18")-C(19")	1.3900
C(4")-H(4"A)	0.9900	C(19")-H(19")	0.9500
C(4")-H(4"B)	0.9900	C(20")-H(20")	0.9500
C(4")-C(5")	1.564(17)	C(21")-C(22")	1.3900

C(21")-C(26")	1.3900	C(36)-H(36A)	0.9900
C(22")-H(22")	0.9500	C(36)-H(36B)	0.9900
C(22")-C(23")	1.3900	C(36)-C(37)	1.537(14)
C(23")-H(23")	0.9500	C(37)-H(37A)	0.9900
C(23")-C(24")	1.3900	C(37)-H(37B)	0.9900
C(24")-C(25")	1.3900	C(37)-C(38)	1.546(14)
C(24")-C(29)#2	1.45(18)	C(38)-H(38A)	0.9800
C(25")-H(25")	0.9500	C(38)-H(38B)	0.9800
C(25")-C(26")	1.3900	C(38)-H(38C)	0.9800
C(26")-H(26")	0.9500	O(4)-C(39)	1.428(13)
Br(1S)-C(1S)	1.86(3)	O(4)-C(30)	1.381(16)
Br(4S)-C(1S)	1.90(4)	C(39)-H(39A)	0.9900
Br(2S)-C(1S)	1.93(4)	C(39)-H(39B)	0.9900
Br(3S)-C(1S)	1.95(4)	C(39)-C(40)	1.529(13)
O(3)-C(33)	1.425(13)	C(40)-H(40A)	0.9900
O(3)-C(28)	1.379(16)	C(40)-H(40B)	0.9900
C(33)-H(33A)	0.9900	C(40)-C(41)	1.512(13)
C(33)-H(33B)	0.9900	C(41)-H(41A)	0.9900
C(33)-C(34)	1.531(12)	C(41)-H(41B)	0.9900
C(34)-H(34A)	0.9900	C(41)-C(42)	1.536(13)
C(34)-H(34B)	0.9900	C(42)-H(42A)	0.9900
C(34)-C(35)	1.512(13)	C(42)-H(42B)	0.9900
C(35)-H(35A)	0.9900	C(42)-C(43)	1.535(14)
C(35)-H(35B)	0.9900	C(43)-H(43A)	0.9900
C(35)-C(36)	1.533(13)	C(43)-H(43B)	0.9900

C(43)-C(44)	1.543(14)	C(27)-C(32)	1.3900
C(44)-H(44A)	0.9800	C(28)-C(29)	1.3900
C(44)-H(44B)	0.9800	C(29)-C(30)	1.3900
C(44)-H(44C)	0.9800	C(30)-C(31)	1.3900
O(5)-C(45)	1.427(13)	C(31)-C(32)	1.3900
O(5)-C(32)	1.426(16)		
C(45)-H(45A)	0.9900	C(12)-N(1)-C(13)	110.3(12)
C(45)-H(45B)	0.9900	C(14)-N(1)-C(12)	128.2(16)
C(45)-C(46)	1.529(13)	C(14)-N(1)-C(13)	119.6(15)
C(46)-H(46A)	0.9900	C(20)-N(2)-C(21)	119.9(19)
C(46)-H(46B)	0.9900	C(2)-C(1)-C(5)	106.1(13)
C(46)-C(47)	1.508(13)	C(2")-C(1)-C(2)	123(3)
C(47)-H(47A)	0.9900	C(2")-C(1)-C(5)	131(2)
C(47)-H(47B)	0.9900	C(1)-C(2)-C(3)	106.6(13)
C(47)-C(48)	1.538(14)	C(1')-C(2)-C(1)	116(3)
C(48)-H(48A)	0.9900	C(1')-C(2)-C(3)	137(2)
C(48)-H(48B)	0.9900	C(2)-C(3)-H(3)	116.6
C(48)-C(49)	1.538(14)	C(4)-C(3)-C(2)	99.4(13)
C(49)-H(49A)	0.9900	C(4)-C(3)-H(3)	116.6
C(49)-H(49B)	0.9900	C(7)-C(3)-C(2)	105.4(13)
C(49)-C(50)	1.545(14)	C(7)-C(3)-H(3)	116.6
C(50)-H(50A)	0.9800	C(7)-C(3)-C(4)	99.6(11)
C(50)-H(50B)	0.9800	C(3)-C(4)-H(4A)	113.0
C(50)-H(50C)	0.9800	C(3)-C(4)-H(4B)	113.0
C(27)-C(28)	1.3900	C(3)-C(4)-C(5)	93.8(11)

H(4A)-C(4)-H(4B)	110.4	C(10)-C(11)-C(6)	120.0
C(5)-C(4)-H(4A)	113.0	C(10)-C(11)-H(11)	120.0
C(5)-C(4)-H(4B)	113.0	O(1)-C(12)-N(1)	125.6(18)
C(1)-C(5)-C(4)	98.9(12)	O(1)-C(12)-C(9)	126.9(19)
C(1)-C(5)-H(5)	116.8	N(1)-C(12)-C(9)	107.2(13)
C(4)-C(5)-H(5)	116.8	O(2)-C(13)-N(1)	126.1(18)
C(6)-C(5)-C(1)	106.3(13)	O(2)-C(13)-C(10)	127.1(19)
C(6)-C(5)-C(4)	98.3(11)	N(1)-C(13)-C(10)	103.3(12)
C(6)-C(5)-H(5)	116.8	C(15)-C(14)-N(1)	122.1(16)
C(7)-C(6)-C(5)	106.2(10)	C(15)-C(14)-C(19)	120.0
C(7)-C(6)-C(11)	120.0	C(19)-C(14)-N(1)	116.7(16)
C(11)-C(6)-C(5)	133.8(10)	C(14)-C(15)-H(15)	120.0
C(6)-C(7)-C(3)	106.8(10)	C(16)-C(15)-C(14)	120.0
C(6)-C(7)-C(8)	120.0	C(16)-C(15)-H(15)	120.0
C(8)-C(7)-C(3)	133.2(10)	C(15)-C(16)-H(16)	120.0
C(7)-C(8)-H(8)	120.0	C(15)-C(16)-C(17)	120.0
C(9)-C(8)-C(7)	120.0	C(17)-C(16)-H(16)	120.0
C(9)-C(8)-H(8)	120.0	C(16)-C(17)-C(20)	117.1(13)
C(8)-C(9)-C(10)	120.0	C(18)-C(17)-C(16)	120.0
C(8)-C(9)-C(12)	132.6(11)	C(18)-C(17)-C(20)	122.9(13)
C(10)-C(9)-C(12)	107.4(11)	C(17)-C(18)-H(18)	120.0
C(9)-C(10)-C(13)	111.2(11)	C(19)-C(18)-C(17)	120.0
C(11)-C(10)-C(9)	120.0	C(19)-C(18)-H(18)	120.0
C(11)-C(10)-C(13)	128.6(11)	C(14)-C(19)-H(19)	120.0
C(6)-C(11)-H(11)	120.0	C(18)-C(19)-C(14)	120.0

C(18)-C(19)-H(19)	120.0	C(20')-N(2')-C(21')	118.9(19)
N(2)-C(20)-C(17)	121.3(18)	C(2)-C(1')-C(2')	124(3)
N(2)-C(20)-H(20)	119.3	C(2)-C(1')-C(5')	131(3)
C(17)-C(20)-H(20)	119.3	C(2')-C(1')-C(5')	105.4(15)
C(22)-C(21)-N(2)	116.9(15)	C(1')-C(2')-C(3')	107.7(15)
C(22)-C(21)-C(26)	120.0	C(1")-C(2')-C(1')	116(3)
C(26)-C(21)-N(2)	123.1(15)	C(1")-C(2')-C(3')	136(3)
C(21)-C(22)-H(22)	120.0	C(2')-C(3')-H(3')	116.9
C(21)-C(22)-C(23)	120.0	C(4')-C(3')-C(2')	99.4(14)
C(23)-C(22)-H(22)	120.0	C(4')-C(3')-H(3')	116.9
C(22)-C(23)-H(23)	120.0	C(7')-C(3')-C(2')	105.3(14)
C(24)-C(23)-C(22)	120.0	C(7')-C(3')-H(3')	116.9
C(24)-C(23)-H(23)	120.0	C(7')-C(3')-C(4')	98.4(13)
C(23)-C(24)-C(25)	120.0	C(3')-C(4')-H(4'A)	112.9
C(23)-C(24)-C(27)	119.8(18)	C(3')-C(4')-H(4'B)	112.9
C(25)-C(24)-C(27)	119.9(18)	C(3')-C(4')-C(5')	94.2(12)
C(24)-C(25)-H(25)	120.0	H(4'A)-C(4')-H(4'B)	110.3
C(26)-C(25)-C(24)	120.0	C(5')-C(4')-H(4'A)	112.9
C(26)-C(25)-H(25)	120.0	C(5')-C(4')-H(4'B)	112.9
C(21)-C(26)-H(26)	120.0	C(1')-C(5')-C(4')	100.2(13)
C(25)-C(26)-C(21)	120.0	C(1')-C(5')-H(5')	116.9
C(25)-C(26)-H(26)	120.0	C(4')-C(5')-H(5')	116.9
C(12')-N(1')-C(13')	109.8(13)	C(6')-C(5')-C(1')	105.6(14)
C(14')-N(1')-C(12')	128.0(17)	C(6')-C(5')-C(4')	97.3(13)
C(14')-N(1')-C(13')	118.9(16)	C(6')-C(5')-H(5')	116.9

C(7')-C(6')-C(5')	106.4(11)	C(15')-C(14')-C(19')	120.0
C(7')-C(6')-C(11')	120.0	C(19')-C(14')-N(1')	118.1(16)
C(11')-C(6')-C(5')	133.5(11)	C(14')-C(15')-H(15')	120.0
C(6')-C(7')-C(3')	107.0(11)	C(14')-C(15')-C(16')	120.0
C(8')-C(7')-C(3')	133.0(11)	C(16')-C(15')-H(15')	120.0
C(8')-C(7')-C(6')	120.0	C(15')-C(16')-H(16')	120.0
C(7')-C(8')-H(8')	120.0	C(17')-C(16')-C(15')	120.0
C(7')-C(8')-C(9')	120.0	C(17')-C(16')-H(16')	120.0
C(9')-C(8')-H(8')	120.0	C(16')-C(17')-C(18')	120.0
C(8')-C(9')-C(12')	132.6(12)	C(16')-C(17')-C(20')	116.1(14)
C(10')-C(9')-C(8')	120.0	C(18')-C(17')-C(20')	123.9(14)
C(10')-C(9')-C(12')	107.3(12)	C(17')-C(18')-H(18')	120.0
C(9')-C(10')-C(13')	111.7(12)	C(19')-C(18')-C(17')	120.0
C(11')-C(10')-C(9')	120.0	C(19')-C(18')-H(18')	120.0
C(11')-C(10')-C(13')	128.3(12)	C(14')-C(19')-H(19')	120.0
C(6')-C(11')-H(11')	120.0	C(18')-C(19')-C(14')	120.0
C(10')-C(11')-C(6')	120.0	C(18')-C(19')-H(19')	120.0
C(10')-C(11')-H(11')	120.0	N(2')-C(20')-C(17')	120.7(19)
O(1')-C(12')-N(1')	126.3(19)	N(2')-C(20')-H(20')	119.7
O(1')-C(12')-C(9')	126(2)	C(17')-C(20')-H(20')	119.7
N(1')-C(12')-C(9')	106.9(14)	C(22')-C(21')-N(2')	116.4(15)
O(2')-C(13')-N(1')	127.7(19)	C(22')-C(21')-C(26')	120.0
O(2')-C(13')-C(10')	128.4(19)	C(26')-C(21')-N(2')	123.5(15)
N(1')-C(13')-C(10')	102.8(13)	C(21')-C(22')-H(22')	120.0
C(15')-C(14')-N(1')	121.9(16)	C(23')-C(22')-C(21')	120.0

C(23')-C(22')-H(22')	120.0
C(22')-C(23')-H(23')	120.0
C(22')-C(23')-C(24')	120.0
C(24')-C(23')-H(23')	120.0
C(23')-C(24')-C(25')	120.0
C(23')-C(24')-C(31)#1	117(5)
C(25')-C(24')-C(31)#1	123(5)
C(24')-C(25')-H(25')	120.0
C(26')-C(25')-C(24')	120.0
C(26')-C(25')-H(25')	120.0
C(21')-C(26')-H(26')	120.0
C(25')-C(26')-C(21')	120.0
C(25')-C(26')-H(26')	120.0
C(12")-N(1")-C(13")	108.4(12)
C(14")-N(1")-C(12")	128.1(16)
C(14")-N(1")-C(13")	121.7(15)
C(20")-N(2")-C(21")	121(2)
C(2')-C(1")-C(2")	123(3)
C(2')-C(1")-C(5")	131(2)
C(2")-C(1")-C(5")	106.2(15)
C(1)-C(2")-C(1")	118(3)
C(1)-C(2")-C(3")	134(2)
C(1")-C(2")-C(3")	106.9(15)
C(2")-C(3")-H(3")	116.7
C(4")-C(3")-C(2")	99.5(14)
C(4")-C(3")-H(3")	116.7
C(7")-C(3")-C(2")	105.4(14)
C(7")-C(3")-H(3")	116.7
C(7")-C(3")-C(4")	99.1(13)
C(3")-C(4")-H(4"A)	112.9
C(3")-C(4")-H(4"B)	112.9
C(3")-C(4")-C(5")	93.9(12)
H(4"A)-C(4")-H(4"B)	110.4
C(5")-C(4")-H(4"A)	112.9
C(5")-C(4")-H(4"B)	112.9
C(1")-C(5")-C(4")	99.2(13)
C(1")-C(5")-H(5")	116.8
C(4")-C(5")-H(5")	116.8
C(6")-C(5")-C(1")	106.6(14)
C(6")-C(5")-C(4")	97.5(13)
C(6")-C(5")-H(5")	116.8
C(7")-C(6")-C(5")	105.9(11)
C(7")-C(6")-C(11")	120.0
C(11")-C(6")-C(5")	133.6(11)
C(6")-C(7")-C(3")	107.1(11)
C(6")-C(7")-C(8")	120.0
C(8")-C(7")-C(3")	132.7(11)
C(7")-C(8")-H(8")	120.0
C(9")-C(8")-C(7")	120.0
C(9")-C(8")-H(8")	120.0

C(8")-C(9")-C(10")	120.0	C(18")-C(17")-C(16")	120.0
C(8")-C(9")-C(12")	132.8(11)	C(18")-C(17")-C(20")	121.3(14)
C(10")-C(9")-C(12")	107.1(11)	C(17")-C(18")-H(18")	120.0
C(9")-C(10")-C(13")	111.2(11)	C(19")-C(18")-C(17")	120.0
C(11")-C(10")-C(9")	120.0	C(19")-C(18")-H(18")	120.0
C(11")-C(10")-C(13")	128.8(11)	C(14")-C(19")-H(19")	120.0
C(6")-C(11")-H(11")	120.0	C(18")-C(19")-C(14")	120.0
C(10")-C(11")-C(6")	120.0	C(18")-C(19")-H(19")	120.0
C(10")-C(11")-H(11")	120.0	N(2")-C(20")-C(17")	119(2)
O(1")-C(12")-N(1")	124.5(18)	N(2")-C(20")-H(20")	120.5
O(1")-C(12")-C(9")	127.1(19)	C(17")-C(20")-H(20")	120.5
N(1")-C(12")-C(9")	108.3(13)	C(22")-C(21")-N(2")	116.0(16)
O(2")-C(13")-N(1")	128.5(18)	C(22")-C(21")-C(26")	120.0
O(2")-C(13")-C(10")	126.7(18)	C(26")-C(21")-N(2")	123.6(16)
N(1")-C(13")-C(10")	104.6(12)	C(21")-C(22")-H(22")	120.0
C(15")-C(14")-N(1")	122.5(16)	C(21")-C(22")-C(23")	120.0
C(15")-C(14")-C(19")	120.0	C(23")-C(22")-H(22")	120.0
C(19")-C(14")-N(1")	116.0(16)	C(22")-C(23")-H(23")	120.0
C(14")-C(15")-H(15")	120.0	C(24")-C(23")-C(22")	120.0
C(14")-C(15")-C(16")	120.0	C(24")-C(23")-H(23")	120.0
C(16")-C(15")-H(15")	120.0	C(23")-C(24")-C(25")	120.0
C(15")-C(16")-H(16")	120.0	C(23")-C(24")-C(29)#2	119(6)
C(15")-C(16")-C(17")	120.0	C(25")-C(24")-C(29)#2	121(5)
C(17")-C(16")-H(16")	120.0	C(24")-C(25")-H(25")	120.0
C(16")-C(17")-C(20")	117.0(15)	C(24")-C(25")-C(26")	120.0

C(26")-C(25")-H(25")	120.0	C(34)-C(35)-C(36)	110.8(14)
C(21")-C(26")-H(26")	120.0	H(35A)-C(35)-H(35B)	108.1
C(25")-C(26")-C(21")	120.0	C(36)-C(35)-H(35A)	109.5
C(25")-C(26")-H(26")	120.0	C(36)-C(35)-H(35B)	109.5
Br(1S)-C(1S)-Br(4S)	111.6(18)	C(35)-C(36)-H(36A)	109.7
Br(1S)-C(1S)-Br(2S)	109.2(18)	C(35)-C(36)-H(36B)	109.7
Br(1S)-C(1S)-Br(3S)	109.7(18)	C(35)-C(36)-C(37)	109.7(14)
Br(4S)-C(1S)-Br(2S)	110.0(18)	H(36A)-C(36)-H(36B)	108.2
Br(4S)-C(1S)-Br(3S)	108.6(18)	C(37)-C(36)-H(36A)	109.7
Br(2S)-C(1S)-Br(3S)	107.8(17)	C(37)-C(36)-H(36B)	109.7
C(28)-O(3)-C(33)	119(2)	C(36)-C(37)-H(37A)	109.7
O(3)-C(33)-H(33A)	108.5	C(36)-C(37)-H(37B)	109.7
O(3)-C(33)-H(33B)	108.5	C(36)-C(37)-C(38)	109.7(14)
O(3)-C(33)-C(34)	115.0(14)	H(37A)-C(37)-H(37B)	108.2
H(33A)-C(33)-H(33B)	107.5	C(38)-C(37)-H(37A)	109.7
C(34)-C(33)-H(33A)	108.5	C(38)-C(37)-H(37B)	109.7
C(34)-C(33)-H(33B)	108.5	C(37)-C(38)-H(38A)	109.5
C(33)-C(34)-H(34A)	109.8	C(37)-C(38)-H(38B)	109.5
C(33)-C(34)-H(34B)	109.8	C(37)-C(38)-H(38C)	109.5
H(34A)-C(34)-H(34B)	108.3	H(38A)-C(38)-H(38B)	109.5
C(35)-C(34)-C(33)	109.2(13)	H(38A)-C(38)-H(38C)	109.5
C(35)-C(34)-H(34A)	109.8	H(38B)-C(38)-H(38C)	109.5
C(35)-C(34)-H(34B)	109.8	C(30)-O(4)-C(39)	113(2)
C(34)-C(35)-H(35A)	109.5	O(4)-C(39)-H(39A)	108.6
C(34)-C(35)-H(35B)	109.5	O(4)-C(39)-H(39B)	108.6

O(4)-C(39)-C(40)	114.5(15)	H(43A)-C(43)-H(43B)	108.0
H(39A)-C(39)-H(39B)	107.6	C(44)-C(43)-H(43A)	109.4
C(40)-C(39)-H(39A)	108.6	C(44)-C(43)-H(43B)	109.4
C(40)-C(39)-H(39B)	108.6	C(43)-C(44)-H(44A)	109.5
C(39)-C(40)-H(40A)	109.4	C(43)-C(44)-H(44B)	109.5
C(39)-C(40)-H(40B)	109.4	C(43)-C(44)-H(44C)	109.5
H(40A)-C(40)-H(40B)	108.0	H(44A)-C(44)-H(44B)	109.5
C(41)-C(40)-C(39)	111.2(14)	H(44A)-C(44)-H(44C)	109.5
C(41)-C(40)-H(40A)	109.4	H(44B)-C(44)-H(44C)	109.5
C(41)-C(40)-H(40B)	109.4	C(32)-O(5)-C(45)	116(2)
C(40)-C(41)-H(41A)	109.2	O(5)-C(45)-H(45A)	108.5
C(40)-C(41)-H(41B)	109.2	O(5)-C(45)-H(45B)	108.5
C(40)-C(41)-C(42)	112.2(15)	O(5)-C(45)-C(46)	115.0(15)
H(41A)-C(41)-H(41B)	107.9	H(45A)-C(45)-H(45B)	107.5
C(42)-C(41)-H(41A)	109.2	C(46)-C(45)-H(45A)	108.5
C(42)-C(41)-H(41B)	109.2	C(46)-C(45)-H(45B)	108.5
C(41)-C(42)-H(42A)	109.1	C(45)-C(46)-H(46A)	109.6
C(41)-C(42)-H(42B)	109.1	C(45)-C(46)-H(46B)	109.6
H(42A)-C(42)-H(42B)	107.9	H(46A)-C(46)-H(46B)	108.2
C(43)-C(42)-C(41)	112.3(15)	C(47)-C(46)-C(45)	110.1(14)
C(43)-C(42)-H(42A)	109.1	C(47)-C(46)-H(46A)	109.6
C(43)-C(42)-H(42B)	109.1	C(47)-C(46)-H(46B)	109.6
C(42)-C(43)-H(43A)	109.4	C(46)-C(47)-H(47A)	109.4
C(42)-C(43)-H(43B)	109.4	C(46)-C(47)-H(47B)	109.4
C(42)-C(43)-C(44)	111.2(15)	C(46)-C(47)-C(48)	111.1(15)

H(47A)-C(47)-H(47B)	108.0	O(3)-C(28)-C(29)	120(2)
C(48)-C(47)-H(47A)	109.4	C(29)-C(28)-C(27)	120.0
C(48)-C(47)-H(47B)	109.4	C(28)-C(29)-C(24")#3	125(7)
C(47)-C(48)-H(48A)	109.5	C(28)-C(29)-C(30)	120.0
C(47)-C(48)-H(48B)	109.5	C(30)-C(29)-C(24")#3	114(8)
C(47)-C(48)-C(49)	110.8(15)	O(4)-C(30)-C(29)	121(2)
H(48A)-C(48)-H(48B)	108.1	O(4)-C(30)-C(31)	119(2)
C(49)-C(48)-H(48A)	109.5	C(29)-C(30)-C(31)	120.0
C(49)-C(48)-H(48B)	109.5	C(30)-C(31)-C(24')#1	121(6)
C(48)-C(49)-H(49A)	109.4	C(32)-C(31)-C(24')#1	119(6)
C(48)-C(49)-H(49B)	109.4	C(32)-C(31)-C(30)	120.0
C(48)-C(49)-C(50)	111.3(15)	C(27)-C(32)-O(5)	120(2)
H(49A)-C(49)-H(49B)	108.0	C(31)-C(32)-O(5)	120(2)
C(50)-C(49)-H(49A)	109.4	C(31)-C(32)-C(27)	120.0
C(50)-C(49)-H(49B)	109.4		
C(49)-C(50)-H(50A)	109.5		
C(49)-C(50)-H(50B)	109.5		
C(49)-C(50)-H(50C)	109.5		
H(50A)-C(50)-H(50B)	109.5		
H(50A)-C(50)-H(50C)	109.5		
H(50B)-C(50)-H(50C)	109.5		
C(28)-C(27)-C(24)	121(2)		
C(28)-C(27)-C(32)	120.0		
C(32)-C(27)-C(24)	119(2)		
O(3)-C(28)-C(27)	118(2)		

Symmetry transformations used to generate equivalent atoms:

#1 -x+1,-y+1/2,z+0   #2 y+1/4,-x+3/4,-z+7/4   #3 -y+3/4,x-1/4,-z+7/4

Table 4. Anisotropic displacement parameters (Å^{2}x 10^3) for CBC **5**. The anisotropic displacement factor exponent takes the form: $-2\pi^2[h^2 a^{*2}U^{11} + ... + 2 h k a^* b^* U^{12}]$

	U^{11}	U^{22}	U^{33}	U^{23}	U^{13}	U^{12}
O(1)	161(19)	126(17)	166(19)	-8(14)	34(15)	-32(15)
O(2)	180(20)	132(17)	180(20)	-63(15)	59(16)	-46(15)
N(1)	174(18)	116(15)	163(18)	-17(13)	39(14)	-37(14)
N(2)	140(20)	98(17)	140(20)	12(14)	16(14)	3(14)
C(1)	133(15)	99(16)	93(16)	17(12)	15(13)	-29(12)
C(2)	150(15)	104(16)	80(16)	29(12)	26(13)	-26(12)
C(3)	151(17)	114(16)	88(16)	10(13)	11(13)	-17(14)
C(4)	130(18)	124(18)	116(16)	4(15)	11(14)	-8(15)
C(5)	131(16)	97(15)	95(16)	28(13)	13(14)	-6(12)
C(6)	108(17)	97(15)	97(16)	19(12)	-7(12)	-3(13)
C(7)	116(17)	118(16)	88(16)	2(13)	3(13)	-15(13)
C(8)	130(20)	122(17)	116(18)	8(14)	1(15)	-13(15)
C(9)	106(18)	116(16)	120(18)	-6(13)	6(13)	-9(14)
C(10)	106(17)	113(16)	133(17)	1(13)	3(14)	-14(14)
C(11)	113(19)	95(16)	111(17)	-2(14)	-3(14)	-5(14)
C(12)	140(20)	113(18)	150(20)	-9(15)	28(16)	-28(16)
C(13)	140(20)	106(17)	127(19)	-9(14)	27(15)	-10(15)
C(14)	220(20)	104(19)	180(20)	1(16)	57(17)	-31(16)
C(15)	260(30)	140(20)	210(30)	17(19)	80(20)	-3(19)
C(16)	250(30)	130(20)	230(30)	38(19)	80(20)	-6(18)
C(17)	230(20)	130(20)	210(20)	18(18)	84(19)	-17(17)

C(18)	220(20)	120(20)	180(20)	35(18)	70(20)	-1(18)
C(19)	220(30)	110(20)	180(20)	1(18)	44(19)	-24(17)
C(20)	200(20)	110(20)	190(20)	6(18)	45(18)	-11(17)
C(21)	114(18)	101(18)	126(19)	3(14)	-7(14)	15(14)
C(22)	119(19)	120(20)	85(18)	-11(14)	-4(14)	2(14)
C(23)	119(18)	130(20)	94(19)	-27(14)	18(15)	-5(15)
C(24)	129(19)	124(17)	115(18)	-10(14)	17(14)	-13(15)
C(25)	140(20)	140(20)	130(20)	20(16)	-8(16)	-34(16)
C(26)	116(19)	140(20)	140(20)	24(16)	-37(16)	-22(16)
O(1')	160(20)	112(15)	170(20)	12(14)	-10(15)	-6(13)
O(2')	220(20)	122(17)	144(19)	-26(14)	25(15)	-35(15)
N(1')	182(19)	154(17)	134(17)	9(13)	14(14)	-19(14)
N(2')	200(20)	100(16)	140(20)	12(14)	-16(19)	-6(15)
C(1')	148(15)	107(16)	82(16)	13(12)	21(13)	-23(12)
C(2')	157(16)	98(16)	103(17)	10(12)	25(13)	-23(13)
C(3')	142(16)	101(15)	108(16)	-8(13)	25(14)	-21(13)
C(4')	144(18)	117(18)	109(16)	-6(14)	21(15)	-27(15)
C(5')	154(17)	115(17)	98(16)	2(12)	10(14)	-35(13)
C(6')	140(18)	102(16)	112(17)	-15(13)	13(14)	-26(13)
C(7')	126(18)	110(15)	106(17)	-18(13)	26(13)	-16(13)
C(8')	114(19)	136(18)	140(20)	-2(15)	8(15)	-12(15)
C(9')	126(19)	130(17)	133(18)	-9(14)	31(14)	0(14)
C(10')	130(19)	114(16)	126(19)	-17(13)	34(14)	-6(14)
C(11')	119(18)	92(16)	120(19)	-21(13)	36(14)	-21(14)
C(12')	150(20)	141(19)	120(20)	-2(15)	29(15)	-3(15)

C(13')	170(20)	120(19)	150(20)	-4(16)	18(15)	-13(15)
C(14')	200(20)	140(19)	150(20)	25(15)	0(19)	-1(18)
C(15')	300(30)	160(20)	160(20)	29(17)	0(20)	-10(20)
C(16')	330(30)	160(20)	150(20)	50(17)	0(20)	20(20)
C(17')	260(20)	140(20)	170(20)	28(16)	0(20)	30(20)
C(18')	260(30)	160(20)	190(20)	31(18)	10(20)	10(20)
C(19')	200(30)	160(20)	190(20)	11(17)	-20(20)	-10(20)
C(20')	220(20)	130(20)	170(20)	25(17)	10(20)	57(19)
C(21')	200(20)	107(18)	160(20)	5(15)	2(17)	-11(17)
C(22')	160(20)	102(19)	140(20)	11(16)	-5(18)	-31(17)
C(23')	180(20)	101(19)	150(20)	-12(16)	-7(18)	-24(18)
C(24')	180(30)	96(19)	150(20)	17(17)	13(18)	-9(18)
C(25')	180(20)	110(20)	140(20)	18(17)	23(18)	7(19)
C(26')	210(20)	95(19)	160(20)	19(15)	-6(18)	2(18)
O(1")	166(18)	158(18)	104(16)	13(13)	22(13)	51(15)
O(2")	126(17)	125(18)	210(20)	4(15)	-26(15)	33(13)
N(1")	145(16)	127(16)	127(16)	11(12)	4(13)	15(13)
N(2")	140(20)	290(30)	190(20)	118(19)	-1(17)	60(20)
C(1")	146(16)	84(16)	120(16)	3(12)	18(13)	-31(13)
C(2")	143(16)	85(16)	129(17)	7(12)	3(13)	-23(13)
C(3")	149(17)	94(17)	106(16)	5(12)	1(14)	-22(13)
C(4")	151(19)	91(16)	136(19)	-17(14)	-12(16)	-14(14)
C(5")	162(17)	103(17)	147(17)	-11(14)	1(14)	-31(14)
C(6")	175(19)	106(18)	136(17)	4(13)	-4(14)	-15(15)
C(7")	165(18)	105(18)	132(17)	6(13)	-2(14)	-28(15)

C(8")	154(19)	88(18)	107(18)	-3(14)	-19(14)	-38(15)
C(9")	145(17)	109(18)	95(16)	-7(13)	-3(13)	12(14)
C(10")	138(17)	104(17)	103(16)	-20(13)	-7(13)	1(14)
C(11")	154(19)	119(19)	121(19)	-26(14)	-1(14)	-9(16)
C(12")	145(19)	120(20)	103(19)	-9(15)	8(14)	21(15)
C(13")	140(18)	120(20)	139(19)	-1(15)	-7(15)	23(15)
C(14")	190(20)	160(20)	140(19)	15(16)	22(16)	17(18)
C(15")	150(20)	180(20)	150(20)	18(17)	1(18)	28(19)
C(16")	170(20)	200(20)	150(20)	14(18)	1(19)	10(20)
C(17")	150(20)	170(20)	160(20)	26(17)	-1(18)	15(19)
C(18")	240(30)	170(20)	160(20)	20(18)	40(20)	-20(20)
C(19")	270(30)	180(20)	180(20)	28(18)	50(20)	0(20)
C(20")	110(20)	210(20)	180(20)	53(18)	0(18)	20(20)
C(21")	160(20)	310(30)	180(20)	140(20)	-12(19)	10(20)
C(22")	220(30)	350(30)	190(20)	130(20)	0(20)	-60(20)
C(23")	230(30)	330(40)	160(20)	140(20)	-10(20)	-50(20)
C(24")	150(30)	310(30)	160(30)	130(20)	20(20)	-30(20)
C(25")	170(30)	330(30)	180(30)	130(20)	0(20)	0(20)
C(26")	140(20)	320(30)	170(20)	140(20)	-10(20)	10(20)
Br(1S)	180(4)	149(4)	148(4)	6(3)	8(3)	-10(3)
Br(4S)	217(5)	170(4)	244(5)	-13(4)	21(4)	-32(4)
Br(2S)	223(5)	183(4)	169(4)	13(3)	-10(3)	17(4)
Br(3S)	215(5)	222(5)	172(4)	2(4)	49(4)	-6(4)
C(1S)	180(4)	149(4)	148(4)	6(3)	8(3)	-10(3)
O(3)	200(20)	142(16)	163(19)	-19(14)	51(15)	-79(15)

C(33)	280(30)	150(20)	220(30)	10(20)	-10(20)	-60(20)
C(34)	310(30)	120(20)	240(30)	-30(20)	-20(30)	-60(20)
C(35)	360(40)	170(30)	330(30)	30(30)	20(30)	-40(30)
C(36)	400(40)	180(30)	400(40)	80(30)	100(40)	-10(30)
C(37)	430(50)	190(30)	440(50)	150(30)	120(40)	10(30)
C(38)	400(50)	110(30)	480(60)	90(40)	100(40)	80(30)
O(4)	172(19)	154(17)	148(16)	-13(13)	22(15)	-41(15)
C(39)	220(20)	220(30)	190(30)	0(20)	40(20)	-50(20)
C(40)	300(30)	280(30)	240(30)	30(30)	40(30)	-90(30)
C(41)	390(40)	300(40)	220(30)	20(30)	10(30)	-80(30)
C(42)	400(40)	330(40)	230(30)	90(30)	-30(30)	-90(30)
C(43)	370(50)	340(40)	270(40)	90(40)	-40(40)	-80(40)
C(44)	440(60)	390(50)	330(60)	170(40)	-110(50)	-90(50)
O(5)	220(20)	104(15)	113(14)	-7(12)	22(14)	-11(14)
C(45)	250(30)	220(30)	240(30)	30(30)	50(20)	30(20)
C(46)	330(40)	380(40)	320(40)	50(30)	100(30)	50(30)
C(47)	360(40)	500(50)	430(40)	50(40)	100(40)	60(40)
C(48)	380(40)	580(60)	530(50)	20(50)	70(40)	40(40)
C(49)	400(50)	630(60)	570(60)	10(60)	70(50)	60(50)
C(50)	460(70)	660(90)	550(70)	0(70)	50(60)	70(70)
C(27)	121(18)	119(16)	125(17)	-1(13)	15(14)	-10(14)
C(28)	140(20)	131(18)	143(18)	-2(14)	30(15)	-35(16)
C(29)	130(20)	122(18)	114(19)	7(14)	10(16)	-22(15)
C(30)	123(18)	127(18)	92(18)	8(14)	-8(15)	-13(15)
C(31)	108(19)	128(19)	105(18)	4(15)	-29(14)	8(15)

C(32)	144(19)	128(18)	117(17)	0(14)	-1(15)	-16(15)

Table 5. Hydrogen coordinates (x 10^4) and isotropic displacement parameters ($Å^2$ x 10^3) for CBC **5**.

	x	y	z	U(eq)
H(3)	5917	5066	1537	141
H(4A)	5617	5462	1708	148
H(4B)	5916	5568	1575	148
H(5)	5788	5706	2617	129
H(8)	5650	4689	2248	145
H(11)	5496	5411	3482	127
H(15)	5291	4176	4091	242
H(16)	5031	3903	4685	239
H(18)	4525	4514	4974	205
H(19)	4785	4787	4380	203
H(20)	4648	3821	5244	198
H(22)	3967	3874	5299	131
H(23)	3720	3557	5808	136
H(25)	4281	3542	7070	166
H(26)	4528	3859	6561	161
H(3')	7055	5154	2478	141
H(4'A)	6953	4892	1561	148
H(4'B)	6832	5190	1495	148
H(5')	6444	4856	1472	147
H(8')	7066	4722	3281	157

H(11')	6373	4381	2150	132
H(15')	6651	3934	4631	247
H(16')	6592	3548	5182	253
H(18')	6652	3105	3717	243
H(19')	6712	3491	3166	221
H(20')	6542	2896	4700	204
H(22')	6708	2614	5224	164
H(23')	6639	2231	5779	171
H(25')	6201	2657	6926	172
H(26')	6271	3040	6371	184
H(3")	6190	5903	3362	140
H(4"A)	6672	5979	3621	151
H(4"B)	6647	5950	2919	151
H(5")	6935	5565	3300	165
H(8")	6018	5624	4376	139
H(11")	6868	5251	4346	158
H(15")	6114	5257	6604	193
H(16")	5976	5005	7403	206
H(18")	6261	4341	6631	230
H(19")	6399	4593	5832	255
H(20")	6216	4413	7799	200
H(22")	6015	4604	8955	301
H(23")	5894	4375	9799	289
H(25")	5462	3861	8830	271
H(26")	5583	4090	7985	253

H(33A)	4071	3652	7913	259
H(33B)	3871	3500	8336	259
H(34A)	3919	3916	8732	267
H(34B)	3880	4059	8113	267
H(35A)	3477	3800	8783	342
H(35B)	3430	3870	8111	342
H(36A)	3457	4324	8293	391
H(36B)	3568	4275	8938	391
H(37A)	3139	4351	9159	425
H(37B)	3125	4036	9074	425
H(38A)	2781	4243	8519	490
H(38B)	2998	4413	8172	490
H(38C)	2984	4098	8087	490
H(39A)	3094	2643	7028	253
H(39B)	3044	2858	7527	253
H(40A)	3046	2458	8191	326
H(40B)	2780	2536	7851	326
H(41A)	3151	2151	7436	364
H(41B)	2879	2225	7115	364
H(42A)	2924	1829	7848	385
H(42B)	2808	2054	8266	385
H(43A)	2533	1986	7205	392
H(43B)	2411	2054	7831	392
H(44A)	2284	1617	7554	584
H(44B)	2589	1533	7496	584

H(44C)	2467	1602	8121	584
H(45A)	4122	2701	5885	283
H(45B)	4244	2936	6268	283
H(46A)	4232	2933	5020	414
H(46B)	4347	3175	5397	414
H(47A)	4631	2768	5034	517
H(47B)	4577	2695	5702	517
H(48A)	4765	3107	6001	596
H(48B)	4813	3188	5335	596
H(49A)	5212	3013	5437	639
H(49B)	5068	2730	5397	639
H(50A)	5338	2746	6247	837
H(50B)	5178	3000	6475	837
H(50C)	5033	2718	6435	837

Experimental Summary for [1+1] Capsule 6 (CCDC 2143947)

The single crystal X-ray diffraction studies were carried out on a Bruker Kappa Photon III CPAD diffractometer equipped with Mo K_{α} radiation (λ = 0.71073 Å). A 0.063 x 0.047 x 0.028 mm colorless block was mounted on a Cryoloop with Paratone 24EX oil. Data were collected in a nitrogen gas stream at 100(2) K using ϕ and ϖ scans. Crystal-to-detector distance was 60 mm using variable exposure time (60s-240s) depending on θ with a scan width of 2.0°. Data collection was 99.5% complete to 23.256° in θ (0.90Å). A total of 49757 reflections were collected covering the indices, -20<=h<=20, -17<=k<=17, -20<=l<=20. 7971 reflections were found to be symmetry independent, with a R_{int} of 0.0908. Indexing and unit cell refinement indicated a primitive, monoclinic lattice. The space group was found to be $P2_1/c$. The data were integrated using the Bruker SAINT software program and scaled using the SADABS software program. Solution by direct methods (SHELXT) produced a complete phasing model for refinement.

All nonhydrogen atoms were refined anisotropically by full-matrix least-squares (SHELXL-2014). All hydrogen atoms were placed using a riding model. Their positions were constrained relative to their parent atom using the appropriate HFIX command in SHELXL-2014. Crystallographic data are summarized in Table 1.

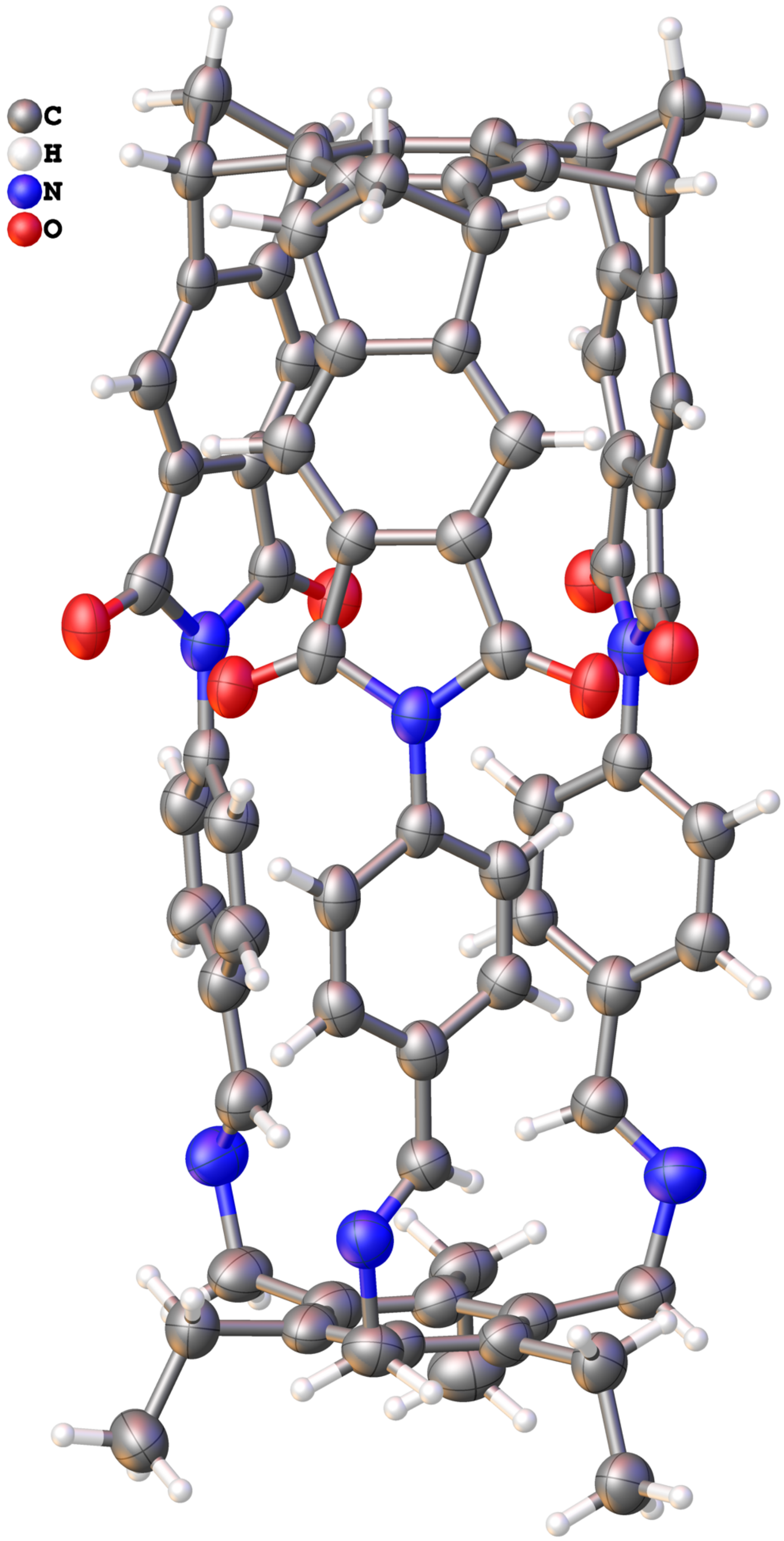
C
H
N
O

Table 1. Crystal data and structure refinement for [1+1] capsule **6**.

Report date	2022-01-23	
Identification code	VagSmallCage	
Empirical formula	C75 H54 N6 O6	
Molecular formula	C75 H54 N6 O6	
Formula weight	1135.24	
Temperature	100.0 K	
Wavelength	0.71073 Å	
Crystal system	Monoclinic	
Space group	P 1 21/c 1	
Unit cell dimensions	a = 18.730(4) Å	α= 90°.
	b = 15.970(3) Å	β= 92.721(4)°.
	c = 18.661(4) Å	γ = 90°.
Volume	5575.6(18) $Å^3$	
Z	4	
Density (calculated)	1.352 Mg/m^3	
Absorption coefficient	0.087 mm^{-1}	
F(000)	2376	
Crystal size	0.063 x 0.047 x 0.028 mm^3	
Crystal color, habit	Colorless Block	
Theta range for data collection	1.973 to 23.256°.	
Index ranges	-20<=h<=20, -17<=k<=17, -20<=l<=20	
Reflections collected	49757	
Independent reflections	7971 [R(int) = 0.0908, R(sigma) = 0.0702]	
Completeness to theta = 23.256°	99.5 %	

Absorption correction	Semi-empirical from equivalents
Max. and min. transmission	0.2565 and 0.2001
Refinement method	Full-matrix least-squares on F^2
Data / restraints / parameters	7971 / 0 / 787
Goodness-of-fit on F^2	1.075
Final R indices [I>2sigma(I)]	R1 = 0.0767, wR2 = 0.1953
R indices (all data)	R1 = 0.1345, wR2 = 0.2291
Extinction coefficient	n/a
Largest diff. peak and hole	0.285 and -0.296 e.Å^{-3}

Table 2. Atomic coordinates (x 10^4) and equivalent isotropic displacement parameters ($Å^2$x 10^3) for [1+1] capsule **6**. U(eq) is defined as one third of the trace of the orthogonalized U^{ij} tensor.

	x	y	z	U(eq)
O(1)	3851(2)	5912(2)	2341(2)	51(1)
O(2)	3955(2)	8687(2)	1695(2)	51(1)
O(3)	3878(2)	9525(2)	3122(2)	50(1)
O(4)	4162(2)	8964(2)	5527(2)	50(1)
O(5)	4107(2)	7204(2)	5339(2)	52(1)
O(6)	4053(2)	4950(2)	3817(2)	51(1)
N(1)	3707(2)	7303(3)	1985(2)	46(1)
N(2)	247(3)	6978(3)	2004(2)	62(1)
N(3)	3821(2)	9205(3)	4330(2)	43(1)
N(4)	357(3)	9511(3)	4155(2)	55(1)
N(5)	3879(2)	6069(3)	4586(2)	45(1)
N(6)	434(3)	6109(3)	4980(3)	57(1)
C(1)	7105(3)	6898(3)	3035(3)	42(1)
C(2)	7102(3)	7764(3)	2876(3)	42(1)
C(3)	7120(3)	8337(3)	3425(3)	42(1)
C(4)	7178(3)	8058(3)	4147(3)	41(1)
C(5)	7196(3)	7217(3)	4294(3)	43(1)
C(6)	7165(3)	6624(3)	3735(3)	42(1)
C(7)	6888(3)	6457(3)	2332(2)	45(1)
C(8)	7274(3)	7025(3)	1813(3)	48(1)
C(9)	6924(3)	7844(3)	2073(3)	45(1)

C(10)	6144(3)	7601(3)	2006(2)	42(1)
C(11)	6122(3)	6731(3)	2177(2)	42(1)
C(12)	5476(3)	6336(3)	2235(2)	44(1)
C(13)	4863(3)	6800(3)	2112(2)	41(1)
C(14)	4894(3)	7651(3)	1935(2)	42(1)
C(15)	5534(3)	8065(4)	1885(3)	46(1)
C(16)	4104(3)	6575(4)	2159(3)	47(1)
C(17)	4162(3)	7976(4)	1847(3)	46(1)
C(18)	2954(3)	7345(3)	2027(3)	44(1)
C(19)	2668(3)	7953(4)	2474(3)	52(2)
C(20)	1939(3)	7956(4)	2554(3)	53(2)
C(21)	1492(3)	7374(4)	2206(3)	54(2)
C(22)	1794(3)	6798(4)	1749(3)	57(2)
C(23)	2515(3)	6780(4)	1664(3)	55(2)
C(24)	732(3)	7378(4)	2358(3)	60(2)
C(25)	-494(3)	7056(4)	2220(3)	64(2)
C(26)	-573(3)	7303(4)	2999(3)	57(2)
C(27)	-562(3)	8149(4)	3191(3)	54(2)
C(28)	-545(3)	8831(4)	2625(3)	60(2)
C(29)	-1291(3)	9140(4)	2378(3)	73(2)
C(30)	6935(3)	9266(3)	3459(3)	44(1)
C(31)	7392(3)	9518(3)	4141(3)	45(1)
C(32)	7063(3)	8821(3)	4611(3)	43(1)
C(33)	6275(3)	9007(3)	4491(2)	40(1)
C(34)	6188(3)	9265(3)	3759(3)	43(1)

C(35)	5518(3)	9391(3)	3443(3)	44(1)
C(36)	4950(3)	9292(3)	3879(3)	43(1)
C(37)	5031(3)	9085(3)	4604(3)	43(1)
C(38)	5700(3)	8932(3)	4921(3)	46(1)
C(39)	4177(3)	9361(3)	3696(3)	46(1)
C(40)	4321(3)	9058(3)	4915(3)	45(1)
C(41)	3064(3)	9169(3)	4359(3)	41(1)
C(42)	2749(3)	8507(3)	4702(3)	46(1)
C(43)	2010(3)	8463(3)	4697(3)	47(1)
C(44)	1583(3)	9071(3)	4364(3)	48(1)
C(45)	1910(3)	9743(3)	4030(3)	47(1)
C(46)	2645(3)	9793(3)	4025(3)	49(1)
C(47)	811(3)	8947(4)	4338(3)	51(1)
C(48)	-415(3)	9276(4)	4128(3)	55(2)
C(49)	-543(3)	8368(4)	3925(3)	51(1)
C(50)	-518(3)	7737(4)	4448(3)	52(2)
C(51)	-515(3)	7980(4)	5237(3)	58(2)
C(52)	-1269(3)	8068(4)	5475(3)	63(2)
C(53)	7077(3)	6724(3)	4978(3)	45(1)
C(54)	7485(3)	5913(3)	4807(3)	48(1)
C(55)	7062(3)	5768(3)	4081(3)	46(1)
C(56)	6306(3)	5814(3)	4330(3)	44(1)
C(57)	6317(3)	6445(3)	4872(3)	45(1)
C(58)	5687(3)	6752(3)	5123(3)	43(1)
C(59)	5060(3)	6402(3)	4857(3)	44(1)

C(60)	5051(3)	5746(3)	4353(3)	45(1)
C(61)	5670(3)	5457(3)	4078(3)	45(1)
C(62)	4319(3)	6622(3)	4984(3)	44(1)
C(63)	4291(3)	5509(3)	4193(3)	42(1)
C(64)	3124(3)	6100(3)	4572(3)	45(1)
C(65)	2734(3)	6068(3)	3919(3)	48(1)
C(66)	2002(3)	6119(3)	3906(3)	49(1)
C(67)	1641(3)	6191(3)	4543(3)	50(1)
C(68)	2035(3)	6214(3)	5192(3)	49(1)
C(69)	2767(3)	6186(3)	5211(3)	46(1)
C(70)	860(3)	6268(4)	4485(3)	54(2)
C(71)	-344(3)	6225(4)	4823(3)	57(2)
C(72)	-515(3)	6887(4)	4249(3)	52(2)
C(73)	-538(3)	6670(4)	3522(3)	53(2)
C(74)	-494(3)	5753(4)	3300(3)	65(2)
C(75)	-1230(3)	5343(4)	3165(4)	75(2)

Table 3. Bond lengths [Å] and angles [°] for [1+1] capsule **6**.

O(1)-C(16)	1.214(6)	C(1)-C(7)	1.527(7)
O(2)-C(17)	1.229(6)	C(2)-C(3)	1.373(7)
O(3)-C(39)	1.213(6)	C(2)-C(9)	1.525(7)
O(4)-C(40)	1.203(6)	C(3)-C(4)	1.419(7)
O(5)-C(62)	1.219(6)	C(3)-C(30)	1.525(7)
O(6)-C(63)	1.207(6)	C(4)-C(5)	1.370(7)
N(1)-C(16)	1.410(7)	C(4)-C(32)	1.517(7)
N(1)-C(17)	1.402(7)	C(5)-C(6)	1.408(7)
N(1)-C(18)	1.419(7)	C(5)-C(53)	1.526(7)
N(2)-C(24)	1.270(7)	C(6)-C(55)	1.528(7)
N(2)-C(25)	1.469(7)	C(7)-H(7)	1.0000
N(3)-C(39)	1.407(6)	C(7)-C(8)	1.534(7)
N(3)-C(40)	1.423(7)	C(7)-C(11)	1.514(7)
N(3)-C(41)	1.423(6)	C(8)-H(8A)	0.9900
N(4)-C(47)	1.274(7)	C(8)-H(8B)	0.9900
N(4)-C(48)	1.492(7)	C(8)-C(9)	1.551(7)
N(5)-C(62)	1.398(7)	C(9)-H(9)	1.0000
N(5)-C(63)	1.410(6)	C(9)-C(10)	1.511(7)
N(5)-C(64)	1.414(7)	C(10)-C(11)	1.428(7)
N(6)-C(70)	1.275(7)	C(10)-C(15)	1.371(7)
N(6)-C(71)	1.485(7)	C(11)-C(12)	1.372(7)
C(1)-C(2)	1.415(7)	C(12)-H(12)	0.9500
C(1)-C(6)	1.376(7)	C(12)-C(13)	1.377(7)

C(13)-C(14)	1.401(7)	C(28)-H(28B)	0.9900
C(13)-C(16)	1.473(8)	C(28)-C(29)	1.532(8)
C(14)-C(15)	1.376(7)	C(29)-H(29A)	0.9800
C(14)-C(17)	1.467(8)	C(29)-H(29B)	0.9800
C(15)-H(15)	0.9500	C(29)-H(29C)	0.9800
C(18)-C(19)	1.402(7)	C(30)-H(30)	1.0000
C(18)-C(23)	1.377(7)	C(30)-C(31)	1.552(7)
C(19)-H(19)	0.9500	C(30)-C(34)	1.531(7)
C(19)-C(20)	1.380(8)	C(31)-H(31A)	0.9900
C(20)-H(20)	0.9500	C(31)-H(31B)	0.9900
C(20)-C(21)	1.390(8)	C(31)-C(32)	1.561(7)
C(21)-C(22)	1.393(8)	C(32)-H(32)	1.0000
C(21)-C(24)	1.465(8)	C(32)-C(33)	1.512(7)
C(22)-H(22)	0.9500	C(33)-C(34)	1.429(7)
C(22)-C(23)	1.369(8)	C(33)-C(38)	1.379(7)
C(23)-H(23)	0.9500	C(34)-C(35)	1.377(7)
C(24)-H(24)	0.9500	C(35)-H(35)	0.9500
C(25)-H(25A)	0.9900	C(35)-C(36)	1.378(7)
C(25)-H(25B)	0.9900	C(36)-C(37)	1.394(7)
C(25)-C(26)	1.520(8)	C(36)-C(39)	1.476(8)
C(26)-C(27)	1.398(8)	C(37)-C(38)	1.383(7)
C(26)-C(73)	1.404(8)	C(37)-C(40)	1.476(7)
C(27)-C(28)	1.519(8)	C(38)-H(38)	0.9500
C(27)-C(49)	1.412(8)	C(41)-C(42)	1.381(7)
C(28)-H(28A)	0.9900	C(41)-C(46)	1.396(7)

C(42)-H(42)	0.9500	C(54)-H(54A)	0.9900
C(42)-C(43)	1.387(7)	C(54)-H(54B)	0.9900
C(43)-H(43)	0.9500	C(54)-C(55)	1.554(7)
C(43)-C(44)	1.385(7)	C(55)-H(55)	1.0000
C(44)-C(45)	1.396(7)	C(55)-C(56)	1.512(7)
C(44)-C(47)	1.458(8)	C(56)-C(57)	1.427(7)
C(45)-H(45)	0.9500	C(56)-C(61)	1.382(7)
C(45)-C(46)	1.379(7)	C(57)-C(58)	1.380(7)
C(46)-H(46)	0.9500	C(58)-H(58)	0.9500
C(47)-H(47)	0.9500	C(58)-C(59)	1.372(7)
C(48)-H(48A)	0.9900	C(59)-C(60)	1.408(7)
C(48)-H(48B)	0.9900	C(59)-C(62)	1.461(7)
C(48)-C(49)	1.515(8)	C(60)-C(61)	1.370(7)
C(49)-C(50)	1.403(8)	C(60)-C(63)	1.489(7)
C(50)-C(51)	1.522(7)	C(61)-H(61)	0.9500
C(50)-C(72)	1.407(8)	C(64)-C(65)	1.392(7)
C(51)-H(51A)	0.9900	C(64)-C(69)	1.401(7)
C(51)-H(51B)	0.9900	C(65)-H(65)	0.9500
C(51)-C(52)	1.507(8)	C(65)-C(66)	1.371(7)
C(52)-H(52A)	0.9800	C(66)-H(66)	0.9500
C(52)-H(52B)	0.9800	C(66)-C(67)	1.400(7)
C(52)-H(52C)	0.9800	C(67)-C(68)	1.389(7)
C(53)-H(53)	1.0000	C(67)-C(70)	1.466(8)
C(53)-C(54)	1.544(7)	C(68)-H(68)	0.9500
C(53)-C(57)	1.498(7)	C(68)-C(69)	1.370(7)

C(69)-H(69)	0.9500	C(70)-N(6)-C(71)	118.3(5)
C(70)-H(70)	0.9500	C(2)-C(1)-C(7)	105.8(4)
C(71)-H(71A)	0.9900	C(6)-C(1)-C(2)	120.6(5)
C(71)-H(71B)	0.9900	C(6)-C(1)-C(7)	132.4(5)
C(71)-C(72)	1.529(8)	C(1)-C(2)-C(9)	106.7(4)
C(72)-C(73)	1.399(8)	C(3)-C(2)-C(1)	119.6(5)
C(73)-C(74)	1.525(8)	C(3)-C(2)-C(9)	132.4(5)
C(74)-H(74A)	0.9900	C(2)-C(3)-C(4)	119.9(5)
C(74)-H(74B)	0.9900	C(2)-C(3)-C(30)	133.0(5)
C(74)-C(75)	1.537(8)	C(4)-C(3)-C(30)	105.9(4)
C(75)-H(75A)	0.9800	C(3)-C(4)-C(32)	106.4(4)
C(75)-H(75B)	0.9800	C(5)-C(4)-C(3)	119.8(5)
C(75)-H(75C)	0.9800	C(5)-C(4)-C(32)	132.6(5)
		C(4)-C(5)-C(6)	120.8(5)
C(16)-N(1)-C(18)	122.7(4)	C(4)-C(5)-C(53)	132.0(5)
C(17)-N(1)-C(16)	110.9(5)	C(6)-C(5)-C(53)	105.7(4)
C(17)-N(1)-C(18)	126.0(5)	C(1)-C(6)-C(5)	119.2(5)
C(24)-N(2)-C(25)	118.4(5)	C(1)-C(6)-C(55)	132.8(5)
C(39)-N(3)-C(40)	110.7(4)	C(5)-C(6)-C(55)	106.8(4)
C(39)-N(3)-C(41)	123.5(4)	C(1)-C(7)-H(7)	116.9
C(41)-N(3)-C(40)	125.8(4)	C(1)-C(7)-C(8)	98.9(4)
C(47)-N(4)-C(48)	117.7(5)	C(8)-C(7)-H(7)	116.9
C(62)-N(5)-C(63)	110.7(5)	C(11)-C(7)-C(1)	104.1(4)
C(62)-N(5)-C(64)	123.5(4)	C(11)-C(7)-H(7)	116.9
C(63)-N(5)-C(64)	125.7(4)	C(11)-C(7)-C(8)	100.3(4)

C(7)-C(8)-H(8A)	112.8	C(15)-C(14)-C(13)	121.8(5)
C(7)-C(8)-H(8B)	112.8	C(15)-C(14)-C(17)	129.5(5)
C(7)-C(8)-C(9)	94.7(4)	C(10)-C(15)-C(14)	116.9(5)
H(8A)-C(8)-H(8B)	110.2	C(10)-C(15)-H(15)	121.6
C(9)-C(8)-H(8A)	112.8	C(14)-C(15)-H(15)	121.6
C(9)-C(8)-H(8B)	112.8	O(1)-C(16)-N(1)	125.1(5)
C(2)-C(9)-C(8)	99.3(4)	O(1)-C(16)-C(13)	128.3(5)
C(2)-C(9)-H(9)	117.1	N(1)-C(16)-C(13)	106.5(5)
C(8)-C(9)-H(9)	117.1	O(2)-C(17)-N(1)	124.2(5)
C(10)-C(9)-C(2)	103.0(4)	O(2)-C(17)-C(14)	129.4(5)
C(10)-C(9)-C(8)	100.3(4)	N(1)-C(17)-C(14)	106.3(5)
C(10)-C(9)-H(9)	117.1	C(19)-C(18)-N(1)	118.3(5)
C(11)-C(10)-C(9)	105.7(4)	C(23)-C(18)-N(1)	120.9(5)
C(15)-C(10)-C(9)	132.1(5)	C(23)-C(18)-C(19)	120.7(5)
C(15)-C(10)-C(11)	122.0(5)	C(18)-C(19)-H(19)	120.9
C(10)-C(11)-C(7)	106.6(4)	C(20)-C(19)-C(18)	118.3(5)
C(12)-C(11)-C(7)	133.1(5)	C(20)-C(19)-H(19)	120.9
C(12)-C(11)-C(10)	120.0(5)	C(19)-C(20)-H(20)	119.2
C(11)-C(12)-H(12)	120.9	C(19)-C(20)-C(21)	121.6(5)
C(11)-C(12)-C(13)	118.2(5)	C(21)-C(20)-H(20)	119.2
C(13)-C(12)-H(12)	120.9	C(20)-C(21)-C(22)	118.3(6)
C(12)-C(13)-C(14)	121.2(5)	C(20)-C(21)-C(24)	118.5(5)
C(12)-C(13)-C(16)	131.2(5)	C(22)-C(21)-C(24)	123.2(5)
C(14)-C(13)-C(16)	107.6(5)	C(21)-C(22)-H(22)	119.5
C(13)-C(14)-C(17)	108.7(5)	C(23)-C(22)-C(21)	121.1(5)

C(23)-C(22)-H(22)	119.5	C(28)-C(29)-H(29A)	109.5
C(18)-C(23)-H(23)	120.1	C(28)-C(29)-H(29B)	109.5
C(22)-C(23)-C(18)	119.9(5)	C(28)-C(29)-H(29C)	109.5
C(22)-C(23)-H(23)	120.1	H(29A)-C(29)-H(29B)	109.5
N(2)-C(24)-C(21)	125.1(6)	H(29A)-C(29)-H(29C)	109.5
N(2)-C(24)-H(24)	117.4	H(29B)-C(29)-H(29C)	109.5
C(21)-C(24)-H(24)	117.4	C(3)-C(30)-H(30)	116.9
N(2)-C(25)-H(25A)	108.5	C(3)-C(30)-C(31)	99.7(4)
N(2)-C(25)-H(25B)	108.5	C(3)-C(30)-C(34)	103.2(4)
N(2)-C(25)-C(26)	114.9(5)	C(31)-C(30)-H(30)	116.9
H(25A)-C(25)-H(25B)	107.5	C(34)-C(30)-H(30)	116.9
C(26)-C(25)-H(25A)	108.5	C(34)-C(30)-C(31)	100.3(4)
C(26)-C(25)-H(25B)	108.5	C(30)-C(31)-H(31A)	113.0
C(27)-C(26)-C(25)	119.7(6)	C(30)-C(31)-H(31B)	113.0
C(27)-C(26)-C(73)	121.2(5)	C(30)-C(31)-C(32)	93.4(4)
C(73)-C(26)-C(25)	118.3(6)	H(31A)-C(31)-H(31B)	110.4
C(26)-C(27)-C(28)	121.0(5)	C(32)-C(31)-H(31A)	113.0
C(26)-C(27)-C(49)	119.2(5)	C(32)-C(31)-H(31B)	113.0
C(49)-C(27)-C(28)	119.8(6)	C(4)-C(32)-C(31)	100.5(4)
C(27)-C(28)-H(28A)	109.0	C(4)-C(32)-H(32)	116.5
C(27)-C(28)-H(28B)	109.0	C(31)-C(32)-H(32)	116.5
C(27)-C(28)-C(29)	113.0(5)	C(33)-C(32)-C(4)	103.7(4)
H(28A)-C(28)-H(28B)	107.8	C(33)-C(32)-C(31)	100.7(4)
C(29)-C(28)-H(28A)	109.0	C(33)-C(32)-H(32)	116.5
C(29)-C(28)-H(28B)	109.0	C(34)-C(33)-C(32)	105.3(4)

C(38)-C(33)-C(32)	132.9(5)	C(46)-C(41)-N(3)	119.6(5)
C(38)-C(33)-C(34)	121.7(5)	C(41)-C(42)-H(42)	120.5
C(33)-C(34)-C(30)	106.6(4)	C(41)-C(42)-C(43)	118.9(5)
C(35)-C(34)-C(30)	132.4(5)	C(43)-C(42)-H(42)	120.5
C(35)-C(34)-C(33)	120.8(5)	C(42)-C(43)-H(43)	119.3
C(34)-C(35)-H(35)	121.8	C(44)-C(43)-C(42)	121.5(5)
C(34)-C(35)-C(36)	116.4(5)	C(44)-C(43)-H(43)	119.3
C(36)-C(35)-H(35)	121.8	C(43)-C(44)-C(45)	118.9(5)
C(35)-C(36)-C(37)	123.2(5)	C(43)-C(44)-C(47)	118.1(5)
C(35)-C(36)-C(39)	129.2(5)	C(45)-C(44)-C(47)	123.0(5)
C(37)-C(36)-C(39)	107.6(4)	C(44)-C(45)-H(45)	119.8
C(36)-C(37)-C(40)	109.3(5)	C(46)-C(45)-C(44)	120.4(5)
C(38)-C(37)-C(36)	120.8(5)	C(46)-C(45)-H(45)	119.8
C(38)-C(37)-C(40)	129.9(5)	C(41)-C(46)-H(46)	120.1
C(33)-C(38)-C(37)	116.9(5)	C(45)-C(46)-C(41)	119.8(5)
C(33)-C(38)-H(38)	121.6	C(45)-C(46)-H(46)	120.1
C(37)-C(38)-H(38)	121.6	N(4)-C(47)-C(44)	124.2(6)
O(3)-C(39)-N(3)	124.3(5)	N(4)-C(47)-H(47)	117.9
O(3)-C(39)-C(36)	128.7(5)	C(44)-C(47)-H(47)	117.9
N(3)-C(39)-C(36)	106.9(4)	N(4)-C(48)-H(48A)	109.0
O(4)-C(40)-N(3)	124.3(5)	N(4)-C(48)-H(48B)	109.0
O(4)-C(40)-C(37)	130.2(5)	N(4)-C(48)-C(49)	113.0(4)
N(3)-C(40)-C(37)	105.4(4)	H(48A)-C(48)-H(48B)	107.8
C(42)-C(41)-N(3)	119.7(5)	C(49)-C(48)-H(48A)	109.0
C(42)-C(41)-C(46)	120.6(5)	C(49)-C(48)-H(48B)	109.0

C(27)-C(49)-C(48)	118.4(5)
C(50)-C(49)-C(27)	119.7(5)
C(50)-C(49)-C(48)	121.0(5)
C(49)-C(50)-C(51)	119.2(5)
C(49)-C(50)-C(72)	120.6(5)
C(72)-C(50)-C(51)	120.1(5)
C(50)-C(51)-H(51A)	109.6
C(50)-C(51)-H(51B)	109.6
H(51A)-C(51)-H(51B)	108.1
C(52)-C(51)-C(50)	110.3(5)
C(52)-C(51)-H(51A)	109.6
C(52)-C(51)-H(51B)	109.6
C(51)-C(52)-H(52A)	109.5
C(51)-C(52)-H(52B)	109.5
C(51)-C(52)-H(52C)	109.5
H(52A)-C(52)-H(52B)	109.5
H(52A)-C(52)-H(52C)	109.5
H(52B)-C(52)-H(52C)	109.5
C(5)-C(53)-H(53)	116.8
C(5)-C(53)-C(54)	99.6(4)
C(54)-C(53)-H(53)	116.8
C(57)-C(53)-C(5)	102.6(4)
C(57)-C(53)-H(53)	116.8
C(57)-C(53)-C(54)	101.5(4)
C(53)-C(54)-H(54A)	112.9
C(53)-C(54)-H(54B)	112.9
C(53)-C(54)-C(55)	94.0(4)
H(54A)-C(54)-H(54B)	110.3
C(55)-C(54)-H(54A)	112.9
C(55)-C(54)-H(54B)	112.9
C(6)-C(55)-C(54)	99.5(4)
C(6)-C(55)-H(55)	117.1
C(54)-C(55)-H(55)	117.1
C(56)-C(55)-C(6)	103.1(4)
C(56)-C(55)-C(54)	99.8(4)
C(56)-C(55)-H(55)	117.1
C(57)-C(56)-C(55)	105.7(4)
C(61)-C(56)-C(55)	132.8(5)
C(61)-C(56)-C(57)	121.0(5)
C(56)-C(57)-C(53)	106.6(5)
C(58)-C(57)-C(53)	132.2(5)
C(58)-C(57)-C(56)	120.6(5)
C(57)-C(58)-H(58)	121.2
C(59)-C(58)-C(57)	117.6(5)
C(59)-C(58)-H(58)	121.2
C(58)-C(59)-C(60)	121.8(5)
C(58)-C(59)-C(62)	130.3(5)
C(60)-C(59)-C(62)	107.8(5)
C(59)-C(60)-C(63)	107.7(5)
C(61)-C(60)-C(59)	121.1(5)

C(61)-C(60)-C(63)	131.1(5)	C(64)-C(69)-H(69)	120.1
C(56)-C(61)-H(61)	121.1	C(68)-C(69)-C(64)	119.8(5)
C(60)-C(61)-C(56)	117.7(5)	C(68)-C(69)-H(69)	120.1
C(60)-C(61)-H(61)	121.1	N(6)-C(70)-C(67)	125.8(5)
O(5)-C(62)-N(5)	124.8(5)	N(6)-C(70)-H(70)	117.1
O(5)-C(62)-C(59)	127.5(5)	C(67)-C(70)-H(70)	117.1
N(5)-C(62)-C(59)	107.6(5)	N(6)-C(71)-H(71A)	108.9
O(6)-C(63)-N(5)	125.1(5)	N(6)-C(71)-H(71B)	108.9
O(6)-C(63)-C(60)	128.9(5)	N(6)-C(71)-C(72)	113.3(4)
N(5)-C(63)-C(60)	106.0(5)	H(71A)-C(71)-H(71B)	107.7
C(65)-C(64)-N(5)	119.9(5)	C(72)-C(71)-H(71A)	108.9
C(65)-C(64)-C(69)	119.8(5)	C(72)-C(71)-H(71B)	108.9
C(69)-C(64)-N(5)	120.3(5)	C(50)-C(72)-C(71)	119.0(5)
C(64)-C(65)-H(65)	120.2	C(73)-C(72)-C(50)	119.7(5)
C(66)-C(65)-C(64)	119.7(5)	C(73)-C(72)-C(71)	120.3(5)
C(66)-C(65)-H(65)	120.2	C(26)-C(73)-C(74)	120.3(5)
C(65)-C(66)-H(66)	119.5	C(72)-C(73)-C(26)	119.6(6)
C(65)-C(66)-C(67)	120.9(5)	C(72)-C(73)-C(74)	120.1(5)
C(67)-C(66)-H(66)	119.5	C(73)-C(74)-H(74A)	108.9
C(66)-C(67)-C(70)	117.7(5)	C(73)-C(74)-H(74B)	108.9
C(68)-C(67)-C(66)	118.8(5)	C(73)-C(74)-C(75)	113.2(5)
C(68)-C(67)-C(70)	123.4(5)	H(74A)-C(74)-H(74B)	107.7
C(67)-C(68)-H(68)	119.6	C(75)-C(74)-H(74A)	108.9
C(69)-C(68)-C(67)	120.9(5)	C(75)-C(74)-H(74B)	108.9
C(69)-C(68)-H(68)	119.6	C(74)-C(75)-H(75A)	109.5

C(74)-C(75)-H(75B)	109.5
C(74)-C(75)-H(75C)	109.5
H(75A)-C(75)-H(75B)	109.5
H(75A)-C(75)-H(75C)	109.5
H(75B)-C(75)-H(75C)	109.5

Table 4. Anisotropic displacement parameters (Å^{2}x 10^3) for [1+1] capsule **6**. The anisotropic displacement factor exponent takes the form: $-2\pi^2[h^2a^{*2}U^{11} + ... + 2h\ k\ a^*b^*U^{12}]$

	U^{11}	U^{22}	U^{33}	U^{23}	U^{13}	U^{12}
O(1)	63(3)	41(2)	48(2)	0(2)	5(2)	-4(2)
O(2)	71(3)	41(2)	42(2)	5(2)	2(2)	2(2)
O(3)	62(2)	52(2)	38(2)	-1(2)	2(2)	-4(2)
O(4)	70(3)	43(2)	37(2)	0(2)	6(2)	-2(2)
O(5)	66(3)	45(2)	47(2)	-2(2)	5(2)	3(2)
O(6)	65(3)	40(2)	48(2)	-5(2)	1(2)	0(2)
N(1)	60(3)	42(3)	37(2)	-1(2)	4(2)	-3(2)
N(2)	55(3)	84(4)	48(3)	-10(3)	0(2)	-9(3)
N(3)	54(3)	39(3)	37(2)	-2(2)	3(2)	-2(2)
N(4)	57(3)	56(3)	51(3)	0(2)	2(2)	4(3)
N(5)	57(3)	34(3)	43(2)	0(2)	2(2)	1(2)
N(6)	54(3)	55(3)	62(3)	-1(2)	5(2)	1(2)
C(1)	44(3)	39(3)	42(3)	1(2)	8(2)	1(2)
C(2)	49(3)	37(3)	40(3)	4(2)	4(2)	-2(2)
C(3)	44(3)	42(3)	42(3)	-2(3)	7(2)	-2(2)
C(4)	44(3)	41(3)	39(3)	-5(2)	2(2)	2(2)
C(5)	45(3)	42(3)	43(3)	1(3)	4(2)	0(3)
C(6)	52(3)	39(3)	36(3)	2(2)	5(2)	2(3)
C(7)	63(4)	40(3)	32(3)	-4(2)	10(2)	0(3)
C(8)	69(4)	42(3)	33(3)	-4(2)	7(2)	-1(3)

C(9)	59(4)	43(3)	34(3)	2(2)	6(2)	-1(3)
C(10)	56(4)	47(3)	23(3)	1(2)	4(2)	0(3)
C(11)	53(4)	43(3)	29(3)	-6(2)	4(2)	-2(3)
C(12)	58(4)	44(3)	31(3)	-6(2)	5(2)	-1(3)
C(13)	54(4)	41(3)	29(3)	-4(2)	6(2)	-6(3)
C(14)	51(4)	48(4)	26(3)	-2(2)	3(2)	1(3)
C(15)	59(4)	49(3)	31(3)	-5(2)	3(2)	0(3)
C(16)	70(4)	41(4)	30(3)	-3(3)	5(3)	2(3)
C(17)	62(4)	48(4)	29(3)	-8(3)	5(2)	-9(3)
C(18)	58(4)	39(3)	35(3)	3(2)	3(2)	-3(3)
C(19)	62(4)	51(4)	44(3)	-4(3)	1(3)	-4(3)
C(20)	59(4)	54(4)	46(3)	-8(3)	2(3)	1(3)
C(21)	62(4)	58(4)	42(3)	-2(3)	5(3)	-2(3)
C(22)	57(4)	66(4)	47(3)	-8(3)	3(3)	-11(3)
C(23)	66(4)	56(4)	42(3)	-6(3)	4(3)	-6(3)
C(24)	63(4)	73(4)	46(3)	-1(3)	3(3)	-3(3)
C(25)	47(4)	90(5)	54(4)	-9(3)	0(3)	-6(3)
C(26)	47(4)	73(5)	51(4)	-9(3)	1(3)	1(3)
C(27)	38(3)	74(5)	49(4)	1(3)	0(2)	-5(3)
C(28)	63(4)	66(4)	50(3)	7(3)	4(3)	1(3)
C(29)	62(4)	88(5)	68(4)	19(4)	-4(3)	2(4)
C(30)	58(4)	31(3)	45(3)	-1(2)	5(2)	-3(3)
C(31)	53(3)	38(3)	46(3)	-2(2)	5(2)	-1(3)
C(32)	57(4)	40(3)	33(3)	-3(2)	3(2)	-1(3)
C(33)	52(4)	35(3)	35(3)	-4(2)	6(2)	-2(2)

C(34)	56(4)	25(3)	48(3)	-4(2)	5(3)	0(2)
C(35)	59(4)	31(3)	44(3)	-4(2)	5(3)	0(3)
C(36)	52(4)	34(3)	43(3)	-2(2)	7(3)	-3(3)
C(37)	48(4)	34(3)	48(3)	-7(2)	5(3)	-2(2)
C(38)	55(4)	37(3)	46(3)	-6(2)	5(3)	-4(3)
C(39)	72(4)	32(3)	35(3)	-5(2)	7(3)	-4(3)
C(40)	56(4)	36(3)	42(3)	-2(3)	-1(3)	-1(3)
C(41)	54(4)	36(3)	34(3)	-4(2)	4(2)	-2(3)
C(42)	59(4)	38(3)	41(3)	-1(2)	1(3)	4(3)
C(43)	54(4)	40(3)	48(3)	-1(3)	6(3)	-2(3)
C(44)	60(4)	45(4)	40(3)	-4(3)	1(3)	3(3)
C(45)	57(4)	41(3)	42(3)	-1(3)	-2(3)	6(3)
C(46)	73(5)	40(3)	33(3)	-1(2)	1(3)	0(3)
C(47)	49(4)	55(4)	48(3)	3(3)	2(3)	1(3)
C(48)	48(4)	58(4)	61(4)	-3(3)	4(3)	5(3)
C(49)	45(3)	60(4)	49(3)	1(3)	6(3)	4(3)
C(50)	46(3)	64(4)	45(3)	-4(3)	5(2)	4(3)
C(51)	62(4)	65(4)	46(3)	-1(3)	3(3)	2(3)
C(52)	64(4)	75(5)	49(3)	-3(3)	2(3)	5(3)
C(53)	58(4)	44(3)	34(3)	0(2)	4(2)	1(3)
C(54)	60(4)	44(3)	39(3)	4(3)	3(2)	5(3)
C(55)	55(4)	37(3)	46(3)	-4(2)	3(2)	2(3)
C(56)	56(4)	37(3)	40(3)	7(2)	4(2)	-3(3)
C(57)	60(4)	40(3)	35(3)	12(2)	1(2)	1(3)
C(58)	54(4)	42(3)	33(3)	8(2)	2(2)	1(3)

C(59)	56(4)	38(3)	37(3)	9(2)	3(2)	1(3)
C(60)	57(4)	38(3)	42(3)	12(3)	10(3)	-1(3)
C(61)	59(4)	37(3)	41(3)	11(2)	7(3)	5(3)
C(62)	56(4)	40(3)	38(3)	3(3)	1(3)	2(3)
C(63)	55(4)	35(3)	37(3)	5(3)	6(2)	4(3)
C(64)	60(4)	35(3)	41(3)	1(2)	5(3)	0(3)
C(65)	63(4)	42(3)	41(3)	1(3)	7(3)	-4(3)
C(66)	56(4)	49(4)	41(3)	-1(3)	-2(3)	-1(3)
C(67)	59(4)	44(3)	47(3)	-1(3)	3(3)	-3(3)
C(68)	62(4)	40(3)	46(3)	-3(3)	7(3)	3(3)
C(69)	55(4)	41(3)	43(3)	-2(2)	-2(3)	5(3)
C(70)	57(4)	55(4)	51(4)	0(3)	5(3)	0(3)
C(71)	49(4)	62(4)	60(4)	0(3)	8(3)	-3(3)
C(72)	41(3)	64(4)	52(4)	-4(3)	4(2)	-4(3)
C(73)	45(4)	61(4)	54(4)	-4(3)	7(3)	-2(3)
C(74)	55(4)	80(5)	59(4)	-15(3)	10(3)	0(3)
C(75)	63(5)	79(5)	85(5)	-17(4)	8(3)	-7(4)

Table 5. Hydrogen coordinates (x 10^4) and isotropic displacement parameters ($Å^2$x 10^3) for [1+1] capsule **6**.

	x	y	z	U(eq)
H(7)	6988	5843	2305	54
H(8A)	7147	6899	1303	57
H(8B)	7799	7020	1899	57
H(9)	7064	8377	1836	54
H(12)	5453	5760	2356	53
H(15)	5552	8644	1772	56
H(19)	2968	8353	2716	62
H(20)	1738	8365	2854	63
H(22)	1493	6411	1492	68
H(23)	2713	6378	1356	66
H(24)	590	7706	2752	73
H(25A)	-739	6513	2133	77
H(25B)	-740	7479	1910	77
H(28A)	-300	8613	2205	72
H(28B)	-263	9310	2821	72
H(29A)	-1248	9567	2005	109
H(29B)	-1528	9381	2786	109
H(29C)	-1573	8669	2183	109
H(30)	7000	9603	3016	53
H(31A)	7288	10091	4310	55

H(31B)	7911	9446	4086	55
H(32)	7249	8786	5121	52
H(35)	5450	9538	2951	53
H(38)	5761	8781	5413	55
H(42)	3035	8089	4936	55
H(43)	1791	8007	4927	57
H(45)	1624	10168	3805	56
H(46)	2865	10249	3795	58
H(47)	636	8412	4464	61
H(48A)	-677	9639	3774	66
H(48B)	-610	9382	4603	66
H(51A)	-261	7546	5530	69
H(51B)	-257	8517	5311	69
H(52A)	-1530	8468	5162	94
H(52B)	-1259	8272	5971	94
H(52C)	-1508	7523	5447	94
H(53)	7213	7008	5443	54
H(54A)	7412	5461	5159	57
H(54B)	8001	6007	4750	57
H(55)	7191	5265	3796	55
H(58)	5688	7190	5467	51
H(61)	5663	5026	3728	55
H(65)	2973	6011	3484	58
H(66)	1738	6106	3460	58
H(68)	1795	6250	5628	59

H(69)	3032	6225	5657	56
H(70)	653	6452	4037	65
H(71A)	-571	6389	5271	68
H(71B)	-554	5684	4663	68
H(74A)	-225	5438	3682	78
H(74B)	-224	5713	2858	78
H(75A)	-1497	5644	2780	113
H(75B)	-1496	5365	3604	113
H(75C)	-1168	4757	3023	113

References

(1) Zhang, Z.; Li, Y.; Song, B.; Zhang, Y.; Jiang, X.; Wang, M.; Tumbleson, R.; Liu, C.; Wang, P.; Hao, X.-Q.; Rojas, T.; Ngo, A. T.; Sessler, J. L.; Newkome, G. R.; Hla, S. W.; Li, X. Intra- and Intermolecular Self-Assembly of a 20-Nm-Wide Supramolecular Hexagonal Grid. *Nat. Chem.* **2020**, *12* (5), 468–474.

(2) Lei, Z.; Gunther, M. J.; Liyana Gunawardana, V. W.; Pavlović, R. Z.; Xie, H.; Zhu, X.; Keenan, M.; Riggs, A.; Badjić, J. D. A Highly Diastereoselective Synthesis of Deep Molecular Baskets. *Chem. Commun.* **2020**, *56* (70), 10243–10246.

(3) Border, S. E.; Pavlović, R. Z.; Zhiquan, L.; Badjić, J. D. Removal of Nerve Agent Simulants from Water Using Light-Responsive Molecular Baskets. *J. Am. Chem. Soc.* **2017**, *139* (51), 18496–18499.

(4) Gunther, M. J.; Pavlović, R. Z.; Fernandez, J. P.; Zhiquan, L.; Gallucci, J.; Hadad, C. M.; Badjić, J. D. Stereo- and Regioselective Synthesis of Molecular Baskets. *J. Org. Chem.* **2019**, *84* (7), 4392–4401.

(5) Maslak, V.; Yan, Z.; Xia, S.; Gallucci, J.; Hadad, C. M.; Badjić, J. D. Design, Synthesis, and Conformational Dynamics of a Gated Molecular Basket. *J. Am. Chem. Soc.* **2006**, *128* (17), 5887–5894.

(6) Yuen, A. K. L.; Jolliffe, K. A.; Hutton, C. A. Preparation of the Central Tryptophan Moiety of the Celogentin/Moroidin Family of Anti-Mitotic Cyclic Peptides. *Aust. J. Chem.* **2006**, *59* (11), 819–826.

(7) Du, Y.-R.; Xu, B.-H.; Pan, J.-S.; Wu, Y.-W.; Peng, X.-M.; Wang, Y.-F.; Zhang, S.-J. Confinement of Brønsted Acidic Ionic Liquids into Covalent Organic Frameworks as a Catalyst for Dehydrative Formation of Isosorbide from Sorbitol. *Green Chem.* **2019**, *21* (17), 4792–4799.

(8) Nguyen, Q. P. B.; Le, T. N.; Kim, T. H. 2-Aminothiazolinium Based Tripodal Receptors: Synthesis and Recognition of Oxoanions. *Bull. Korean Chem. Soc.* **2009**, *30* (8), 1743–1748.
